# Tangency and multiple factors of violence against lecturer: nuances of the experience in pedagogical practices in health education

**DOI:** 10.1590/0034-7167-2021-0865

**Published:** 2022-12-16

**Authors:** Angela Gilda Alves, Flaviane Cristina Rocha César, Maria Alves Barbosa, Lizete Malagoni de Almeida Cavalcante Oliveira, Dolors Rodríguez-Martín, Edinamar Aparecida Santos da Silva, Johnatan Martins Sousa, Sara Oliveira Souza

**Affiliations:** I Faculdade Sul Americana. Goiânia, Goiás, Brazil; II Universidade Federal de Goiás. Goiânia, Goiás, Brazil; III Universidade de Barcelona. Barcelona, Catalunya, Spain

**Keywords:** Violence, Faculty, Health, Universities, Workplace Violence, Violencia, Docentes, Salud, Universidades, Violencia Laboral, Violência, Docentes, Saúde, Universidades, Violência no Trabalho

## Abstract

**Objective:**

to identify factors that lead the teacher to experience violence in their pedagogical practice in health education.

**Method:**

research with a qualitative approach, based on the Grounded Theory, conducted with 11 professors of the nursing course of a public university in the central region of Brazil in 2020 and 2021. Online semi-structured interviews were analyzed partially in the light of the Constructivist Grounded Theory.

**Results:**

factors that lead lecturer to experience violence are characterized by institutional culture, gender, professor’s perception of violence, and the triggers that drive students to commit violence. Social status and inequalities lead to positions of domination and, consequently, create a fertile ground for violence.

**Final Considerations:**

analyzing violence under Bourdieu’s theory, it is clear that student violence towards lecturer and the reports contained in this study deserve pedagogical reflection. However, it is necessary to include these discussions as a background in teaching environments.

## RESUMO

**Objetivo:**

identificar fatores que levam o professor a experienciar violência na prática pedagógica em saúde.

**Método:**

pesquisa de abordagem qualitativa ancorada no referencial da Teoria Fundamentada nos Dados com 11 docentes do curso de Enfermagem de uma universidade pública da região central do Brasil em 2020 e 2021. Entrevistas semiestruturadas *online* foram analisadas parcialmente à luz da Teoria Fundamentada nos Dados Construtivista.

**Resultados:**

fatores que levam os docentes a experienciar a violência são caracterizados por cultura institucional, gênero, percepção de violência pelo docente e gatilhos que ensejam os estudantes a praticarem violência. Origem social convertida em desigualdades desencadeia posições de dominação e, consequentemente, constitui-se em terreno fértil para a violência.

**Considerações finais:**

analisando a violência sob os fundamentos de Pierre Bourdieu, percebe-se que a violência de alunos contra professores merece pedagogicamente reflexões sobre os relatos contidos nas entrevistas. Contudo, faz-se necessário perpassar tais discussões como pano de fundo nos palcos de ensino.

## RESUMEN

**Objetivo:**

identificar los factores que hacen que el profesor experimente violencia en la práctica pedagógica en salud.

**Método:**

es una investigación de enfoque cualitativo realizada de 2020 a 2021 entre 11 docentes del curso de Enfermería de una universidad pública de la región central de Brasil, mediante entrevistas semiestructuradas en línea que fueron analizadas parcialmente a la luz de la Teoría Fundamentada en Datos Constructivista.

**Resultados:**

Los factores que llevan a los profesores a sufrir violencia están caracterizados por la cultura institucional, el género, la percepción de la violencia por parte de los profesores y los desencadenantes que incitan a los alumnos a practicar tal violencia. El origen social convertido en desigualdades desencadena posiciones de dominación y, en consecuencia, se constituye en un terreno fértil para la violencia.

**Consideraciones finales:**

Al analizar la violencia bajo los fundamentos de Pierre Bourdieu, es posible percibir que la violencia de los alumnos contra los profesores merece una reflexión desde el punto de vista pedagógico sobre los informes contenidos en las entrevistas. Sin embargo, es necesario insertar estos debates en las diferentes etapas de la enseñanza.

## INTRODUCTION

There are different categories of violence, but there is still no consensus on the type, causes, and characteristics of violence against lecturer. The World Health Organization (WHO) defines violence as the intentional use of physical force, threatened or actual, against a person, group, or community, that results in physical or psychological harm^(1)^, which often occurs to lecturer.

Teaching is an inherently complex practice, as it involves theoretical and practical aspects, but is not limited to them. Being a teacher requires committing to the formation of subjects and, critically speaking, contributing to the emancipation of thought.

In this context, the academic organization and the activities developed by lecturer demonstrate aspects inherent to teaching. However, institutions are currently threatening places to work, due to the increase in violence and its various nuances^(2)^.

A study that aimed to identify the association between socio-demographic, work, and school environment factors and the occurrence of physical violence against lecturer in the school environment in the South region of Brazil revealed that, of the 789 lecturer interviewed, 7.9% reported attempted or actual physical violence, 0.8% mentioned bladed weapons, and 0.5% referred to firearms in the school environment^(3)^.

In the context of higher education, professors have never had an easy task and, lately, the complexity of their work has increased, which can lead to suffering^(3)^. Dissatisfaction, lack of partnership and collaboration, competition between colleagues, verbal abuse, excessive demands, and injustice are possible triggers to suffering at work and are caused by power relationships that can affect the health of workers and contribute to the decay of the teaching profession^(4)^.

In this context, a literature review aimed at analyzing the teaching activity and its constituent elements in Brazilian public universities and relating it to mental illness found that precarious work conditions and work overload, flexibilization of labor relations, financing shortage, excess of institutional control, poor infrastructure and violence are factors that affect the mental health of lecturer, who can develop Burnout Syndrome and Common Mental Disorders^(5)^.

The increase in violence in the form of blaming for the environment in which students live and for the problems they face seems to veil devastating causes and effects. This characterizes the phenomenon as symbolic violence, which is not perceived by the lecturer. The concept of symbolic violence proposed by Pierre Bourdieu^(6)^ is defined as “subtle violence”, in which the victim does not perceive the aggression and sometimes acts condescendingly. Therefore, the literature^(7)^ points to the urgent need to give visibility to the phenomenon of symbolic violence in pedagogical relationships in higher education.

In this context, considering there is a lack of research on violence against lecturer^(8)^ and a need to elucidate the factors associated with episodes of violence in the context of universities, in order to contribute to the development of prevention actions^(9)^, the present study aims to answer the following question: What are the factors that lead the teacher to experience violence in their pedagogical practice in health education?

## OBJECTIVE

To identify factors that lead the teacher to experience violence in their pedagogical practice in health education.

## METHODS

### Ethical aspects

The research was approved by the Research Ethics Committee with Human Beings of the Universidade Federal de Goiás. Participants signed the Informed Consent Form (ICF) electronically, according to the recommendations of resolution 466/2012. The identity of the professors who participated in the study was preserved through codes composed of the initial “E”, followed by a number referring to the order of the interviews.

### Type of study and theoretical and methodological framework

This study had a qualitative approach based on the theoretical and methodological framework of Grounded Theory (GT), a method that aims to construct a theory based on a phenomenon that emerges from the concomitant collection and analysis of data for the understanding of experiences and meanings^(10)^. The recommended steps for the dissemination of qualitative studies were followed, according to the *Consolidated criteria for reporting qualitative research* (COREQ)^(11)^.

### Study setting

The research was carried out in the Nursing School of a public university in the central region of Brazil.

### Data source

Eleven permanent professors of the nursing course of a public university in the central region of Brazil participated in the study. The participants were selected by non-probabilistic convenience sampling. The inclusion criteria were: professors aged 18 or over who were willing to participate, and who had at least three months of experience in the position/function. The exclusion criteria were being on vacation, work leave, and/or absent from work during the data collection period^(12-13)^. The main researcher invited 40 professors to participate in the study, through an e-mail that explained the objectives, methodology, and ethical aspects of the research. After a lack of response to the invitation after three contact attempts, 11 professors accepted.

### Data collection and organization

Data were collected from September 2020 to February 2021, through a semi-structured interview with questions recorded in the Google Forms platform and applied and recorded by the researcher via Google Meet, on a date and time scheduled at the convenience of the interviewee, due to the COVID-19 pandemic declared by the World Health Organization (WHO) in January 2020.

The interview script was based on a theoretical review of interviews^(14-15)^ and a theoretical framework on the dimensions of violence^(2-3,7,16-17)^ associated with the objective of the study and consisted of 16 open and closed-ended questions addressing socio-demographic data and the experiences of each participant with violence.

The latter theme was addressed in questions about the concept and triggering factors of violence; violence experienced in pedagogical practice; approach to the theme in pedagogical projects in professional training; if the teacher develops experiences aimed at respecting the entire academic community; and their opinions on: what was most impressive about violence against lecturer, what they consider as violence, what motivates the student to use violence against the teacher, and their vision on possible coping strategies for students’ violence against the teacher.

### Data analysis

In the preparation for data analysis, the recorded interviews were transcribed and the data was organized by NVivo, a qualitative analysis software that optimizes the interpretation of unstructured information by allowing the analysis and presentation of results with structural matrices, coding, auto coding, classification in a database and elaboration of maps and figures^(11)^.

The Constructivist Grounded Theory (TFDC) was used^(2)^ up until Charmaz’s initial and focused coding stages^(18)^. It is important to highlight that this method of analysis was not used to produce a theory based on the data, but to employ the analytical order proposed by the author, which divides the analysis process into two moments - initial (open) and focused. These moments unfold in different phases^(10)^.

The initial coding gave rise to preliminary codes for ordering the preparation of the analysis with the software NVivo. In the next phase, phrases and words are valued and all findings found in the raw data are considered.

The first exploration of the raw data seems to be a simple analysis, but not a simplistic one. In the initial coding, after fragmentation and while revisiting the codes, the researcher can employ attentive observation to find highlights during the comparisons, differences, and similarities.

In this study, the initial coding generated 502 primary codes. After an exhaustive analysis by fragmenting words and grouping them into properties and dimensions^(19)^, these codes were divided into seven subcategories. The codes were incorporated into others because they are not separate things, but properties. The method of looking for more properties helped in the development of the focused code.

In the focused coding phase, certain codes contained in the interviewees’ words are highlighted and organized into categories generated according to the understanding and inferences of the researcher. The initial review of the properties and dimensions of the emerging codes was intertwined with the theoretical concepts of Bordieu and grouped into three categories. The category constructs were saturated by depth and tangentially associated, generating the central category.

## RESULTS

### Socio-demographic characteristics of study participants

All of the 11 professors participating in the study, work in the *lato* and *strictu* sensu undergraduate and graduate programs in nursing. Five (45.4%) of these professors also work in other graduate courses at the institution, such as teaching in health, collective health, and health sciences. Women were predominant, with eight female participants (72.7%), the mean age was 38.9 years, six (54.5%) participants had more than fifteen years in the profession and all had a doctorate (100%) and worked exclusively in the institution.

### Categorization

**Figure 1:**
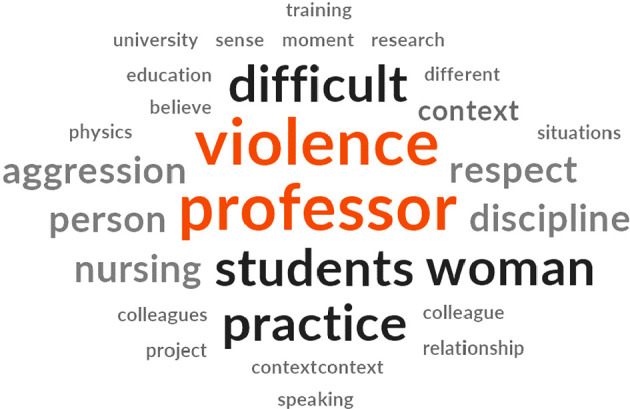
Word cloud of contextualization of violence against lecturer generated by the software Nvivo, Goiania, Goiás, Brazil, 2020-2021

The words that stood out during the first use of Nvivo for the construction of the raw data for the phenomenon studied were the ones with the highest centrality, which is corroborated by the word cloud. Violence, teacher, students, work, and aggression are directly linked. The words located on the edges were: nursing, respect, discipline, difficult, practice, woman, and context. These peripheral elements contextualize the dimensions of violence against lecturer, resulting in the description of the central category, shown in figure 1.

Conceptually, tangency is the general approach to a given topic, without delving into the discussion of the thematic axis^(20)^. In this study, after identifying multiple types and forms of the phenomenon, the intuitive difference between its various elements gave rise to the central category “Tangency and multiple factors of violence against lecturer and the nuances of the experience in pedagogical practices in health education” and to the three subcategories, with their respective focused codings, as described in Chart 1. The representation speeches are highlighted in the description of the subcategories.

**Chart 1:** Synthesis of the expanded categorization, Tangency, and multiple factors of violence against lecturer: nuances of the experience in pedagogical practices in health education, Goiânia, Goiás, Brazil, 2020-2021

Central category	Subcategories	Focused coding
Tangency and multiple factors of violence against lecturer: nuances of the experience in pedagogical practices in health education	Action and reaction: violence does not happen alone	Disrespect as an expression of violence and its relationship with hierarchy (E1, E2, E3, E4, E5, E6, E8, E10, E11).
		ICT as a current and veiled language of violence (E4, E5, E7, E8, E9, E10).
		Need for teacher training to deal with violence in the academic environment (E1, E2, E3, E4, E5, E6, E7, E8, E9, E10, E11).
		Repression and intimidation as an expression of fear and limitation to confrontations (E2; E4; E6; E7; E9; E11).
	Institutional culture and gender	Gender violence related to cultural history (man-woman). Nursing is a female profession (E4, E7, E9, E10, E11).
		Institutional violence, symbolic or not, expressed by issues of domain, rules, regulations, and values (E1, E2, E3, E4, E6, E7, E8, E9, E10, E11).
	Perception of violence against the teacher	The nuances of physical, mental, cultural, and social aggression (E9, E10, E11).
		The fragility of interpersonal relationships and the teacher’s noticeable illness process (E2, E3, E4, E5, E7, E9, E11).

### Action and reaction: violence does not happen alone

Although in their veiled speech they cry out for respect and appreciation, disrespect is the word expressed by the interviewees, revealing that violence is increasingly common in the relationships between lecturer and students, sometimes even seen as trivial, as expressed in the speeches:

[…] *not that the teacher is superior to the student, but there must be respect for the teacher* […] *the concept of violence is very broad* […] *it includes physical violence, emotional violence* […]. (E11)

[…] *when you are questioned from a theoretical, methodological, or pedagogical point of view in a disrespectful way* […]. (E2)

[…] *when they have an opportunity to confront a leader, which, in this case, is the teacher or someone from the school management, they act with violence because they have already experienced some type of violence before* […]. (E8)

*Violence is any external aggression* […] *about respect, about academic violence, both upwards and downwards* […]. (E5)

It is worth noting that the lecturer reported that ICTs were a predominant tool for violence, used by students, professional colleagues, and managers. In the absence of dialogue, digital tools were identified as a trigger to incite lecturer, hindering pedagogical practice and conviviality within the institution.

[…] *the relationships mediated by the quick information provided in apps, in short sentences, have generated some communication problems; sometimes, I receive messages and e-mails from students that make me feel disrespected* […]. (E8)

[…] *I see manifestations of explicit violence, you know, on social networks, whatever they are, WhatsApp groups, Whatsapp groups, and it’s a terrible thing* […]. (E10)

[…] *I think that these issues of slander and defamation, exposure on social networks, you know, this breach of trust, like when we previously agree that we will not record and you are recording* […]. (E11)

It is relevant to emphasize that, regardless of the technological tool used by students to violently attack lecturer, this study revealed that violence against lecturer arouses serious concerns. Emotions and feelings can be expressed in different ways and perceived by the other by the nuances of the language in the speeches:

[…] *because they get very nervous, they don’t know if they will get the job done, so, you know, we have our fears, our difficulties* […]. (E2)

[…] *to not pick up the fight, I get very reflective* […] *but I know that there are factors today that are much broader than just the act of physically hurting someone* […] *speaking, mentally speaking, or even the issue of moral harassment, psychological pressure that the person experiences, we feel psychologically hurt* […]. (E4)

[…] *regarding the student, I felt so intimidated that I didn’t do anything, I was just really scared* […]. (E6)

[…] *I’ll tell you the truth, I’m scared all the time because everything, everything I say, everything I do, it seems that I’m committing abuse for some reason; unfortunately, they do not talk about violence against men* […] *men in nursing seek psychological support because the pressure is very high, I think, the pressure from society* […]. (E11)

[…] *what is more noticeable is that lecturer are getting sick, I can’t even say if it is Burnout Syndrome, but it’s that disbelief, that discouragement you feel when you know you have to teach a very aggressive class, with aggressive students, that keeps you from feeling pleasure in your work* […]. (E9)

### Institutional culture and gender

The universe of the participants’ speeches unveils the gender violence within the institutional culture, both between peers and between students and lecturer, as shown in the speeches:

[…] *which is a violence from management* […] *unfortunately, women are still submissive to men in several aspects, right, due to our* […] *it is very sexist, patriarchal, masculine, so that makes women susceptible to situations of violence* […]. (E9)

[…] *violence that is institutional, and not only in teacher/student relationships* […]. (E2)

[…] *at the university, I am being violated much more by public policies* […]. (E4)

[…] *there was a very consolidated group* […] *I felt attacked several times, specifically by a colleague* […]. (E10)

[…] *you are a man, you can’t be in your office alone with a woman* […] *do not hug the students* […] *the nursing profession has to know that there are men, and that they are a minority, and as a minority, they experience all types of violence* […] *the issue of situatedness is extremely violent; when I hear this in nursing I panic because I can never say anything, because it is not my place to say, as I am a man and most here are women.* (E11 )

### Perception of violence against the lecturer

Participants expressed that violence against lecturer is associated with cultural, social, and current historical issues that differ from the time when they were students. They also reported that violence can manifest itself in physical, verbal, or emotional forms, and is associated with aspects related to students, such as previous experiences of violence and bullying by the student towards the teacher, as revealed in the speeches:

[…] *so I think that the risk factors for violence have a very strong connection with the culture* […] *I think that if you are a very strict, very inaccessible, very God-like teacher in your class, you know, if what you say it’s right and that’s it, it’s over, if you are not open to learning, to do things again, to reformulate, then* […]*, this is what triggers these aggressions, you know.* (E9 )

[…] *sometimes we experience violence every day and we do not know it is violence, so it is difficult to conceptualize* […] *external factors are the biggest triggers of violence, especially social conditions, inequalities, and injustice* […]. (E4)

[…] *I believe that the students we work with today are very different students, very different from the students we were somewhere in the past, so I believe that this is a generational issue* […]. (E3)

*I understand violence as any type of aggression, not necessarily physical, but physical as the greatest expression of violence* […]. (E7)

*I understand violence as any act, whether expressed in words, in attitudes* […] *physical attacks, you can emotionally attack someone, and violence for me is synonymous with aggression* […]. (E10)

*I think that we can’t disregard the life story, the student’s life context, you know, sometimes they already have an experience of a very violent relationship, either with their parents or with their colleagues, or in the educational institutions where they studied, so I think that their previous experiences affect this* […]. (E2)

*Look, sometimes there is bullying from students towards the teacher, with verbal aggression, violence against the teacher* […]. (E6)

## DISCUSSION

Violence is multifactorial and, despite being a broad topic, some of its dimensions are tangent to its concept^(8,17)^. During the data analysis process, it was possible to come to know the phenomena associated with violence against the interviewed professors during their professional practice in health education, whether in an undergraduate course or in a *lato* or *strictu sensu* graduate course. These phenomena include social, cultural, institutional, and technological aspects and gender relations, which gave rise to the central category *Tangency and multiple factors of violence against lecturer: nuances of the experience in pedagogical practices in health education* and the subcategories, which will be discussed in the light of Bourdieu’s theory^(6)^, subdivided into Habitus, Field, and Capital, as it can reach the breadth of the theme.

Faced with violence or threatened violence presented so far, people develop individual and/or collective protective measures, due to an innate sense of self-preservation or defense of cultural and social heritage. That is what is addressed in the subcategory *Action and reaction: violence does not happen alone*, which is based on the perspective of the lecturer interviewed, who reinforce, in their speeches, that information technology and respect are the basis for a culture of peace in the university environment.

Bourdieu^(21)^ suggests that cultural differences between students and lecturer from different social classes would be less evident in the academic environment, as students from middle and lower classes who reach this level of the education system would have already gone through a natural selection process, in which those who least distanced themselves from school culture would have survived.

In this sense, the lecturer interviewed consider disrespect as a seminal moment to characterize the violence they experience. Although in their veiled speech they cry out for respect and appreciation, disrespect is the word expressed by the interviewees, revealing that violence is increasingly common in the relationships between lecturer and students, sometimes even seen as trivial.

Disrespectful behavior between students and lecturer and between students themselves not only hinders interpersonal relationships, but also has consequences for the teaching-learning process, reinforcing students’ indiscipline and overloading lecturer^(22)^.

On the other hand, although Information and Communication Technologies (ICTs) can complement the teaching and learning process, offering activities in digital format according to the needs of each student, they can also be used as weapons for the practice of violence against lecturer, according to the participants.

It is worth noting that the professors reported that ICTs were a predominant tool for violence, used by students, professional colleagues, and managers. In the absence of dialogue, digital tools were identified as a trigger to incite lecturer, hindering pedagogical practice and conviviality within the institution.

A study carried out with 1,534 students from six schools in the states of São Paulo, Ceará, Paraná, and Minas Gerais showed that 37% of the participants were involved with cyberbullying, with 23% as victims, 3% as perpetrators, and 11% in both situations, as victims and as perpetrators^(23)^. This result confirms that technological tools have been being used as a tool for violence in a virtual environment.

During the interviews, the professors expressed that fear is a constant feeling in their pedagogical practice. The same result was found in another study^(24)^, in which at least one-third of the lecturer had already felt fear in the work environment. Those who have already experienced violence are more likely to feel fear at school, which was also found in a study carried out with 25 lecturer in the North region of Brazil, which pointed out that lecturer work in an environment marked by fear and insecurity, triggered by school violence^(25)^.

According to AL-Omari; Choo^(26)^, a work environment is any place where an individual acts effectively or temporarily, performing a task, and where management and interpersonal relationships have a direct impact on violence and on the productivity and emotional state of employees.

Therefore, *Institutional culture and gender* is the subcategory that addresses violence from the perspective of institutional culture, considering that public or private institutions have characteristics associated with the behavior of employees and with how internal (workers) and external customers are treated. Institutional culture is composed of a complex set of values, beliefs, habits, principles, and actions shared within the institution or company, which are part of society’s culture and shaped by history and by everything we learn during social interaction with certain groups^(27)^.

A violent workplace presents conflict between peers and psychological aggression, which influence the types of violence that will occur, especially when employees stay in a stressful environment and work for excessive hours^(26)^. In this study, the characteristics of the institutional culture of the public service are addressed, as it is the workplace of the group of lecturer participating in the research.

The subcategory *Institutional culture and gender* reveals that institutional violence was reported by the professors because it occurs, in many cases, inside the walls of the educational institution, and is sometimes naturalized, symbolic or invisible. It is manifested in a variety of behaviors in the workplace, in the form of discrimination, indifference, criminality, social violence, moral harassment, and even abandonment of teaching and learning. These long-standing violent behaviors can be generically described by the technical term Mobbing, a type of Bullying that occurs in relationships between adults in the work environment^(16,28)^.

Although this type of violence is more visible and discussed today, when analyzing the subject, it is possible to observe that institutional violence is not a contemporary problem, as its characteristics have been historically constructed according to social relationships and practices.

Violence is also manifested as power over the other, as part of the exercise of institutional authority, and can originate in the process of conquering that power. In this sense, Bourdieu^(6)^ brings to light the submission of humans to the socialization process that turns them into social beings that are transformed over time and constituted by learning elements that transform their perception and way of acting depending on their life experiences.

Vertical or horizontal violence is expressed in the speeches of the interviewed lecturer by reports that show domination from one class, for example, the managers, over the other, the lecturer, or vice-versa, as explained in Bourdieu’s theory^(21)^. A study by Abramovay^(29)^ corroborates these findings by reporting that vertical power strengthens violence between peers and/or groups, restricts individual autonomy when used to search for standardized conduct, and ignores or silences collective subjects of the academic community.

Some researchers unanimously agree that the teaching-learning process is imbued with affectivity and feelings of satisfaction with the teaching-learning relationship, as well as frustration, low self-esteem, and even sadness. These feelings are common to human beings’ experiences and thoughts^(30-31)^.

However, institutional violence can kill the dreams of those who seek to get, through the teaching profession, social ascension, decent work, and a peaceful and comfortable life. The lack of this perspective can lead to illness and death^(6,19,21,31-34)^. Therefore, violence in the workplace should not be accepted. Managers must develop prevention strategies and promote good institutional relationships^(26,35)^.

In this context, the universe of the participants’ speeches unveils the gender violence within the institutional culture, both between peers and between students and lecturer. However, female professors are more afraid of experiencing violence^(24)^. In this sense, male lecturer are more frequently the target of obscene comments and gestures and threats with and/or without weapons when compared to women, and are less likely to report when these acts occur^(36)^.

When the interviewees were asked about the relationship between gender and the work environment, some speeches showed a perceived disrespect against male lecturer in a predominantly female profession. The subcategory *Perception of violence against the teacher* addresses the perception of violence against the teacher. A study carried out to demonstrate the relationship between negative working conditions in the psychosocial domain, violent situations and behaviors and bullying at work, and self-perceived mental health of professors of medical and nursing courses revealed that professors of nursing are more exposed to all types of violence and psychological harassment than professors of medicine^(37)^.

Aggression: triggers for violence is a category focused on speeches that address the reasons that drive students to commit violence against the teacher. Being a teacher today is stressful, exhausting, and makes you a target for violence^(38)^, seeing that 80% of these professionals have already experienced a violent situation in the teaching environment^(39)^.

The profile of today’s students is different from past generations. Young people are more intolerant and violence against lecturer has become a global phenomenon that can be harmful to the physical and psychological well-being of professionals^(36)^. The violent behavior of students towards lecturer is related to their social environment, external community, and domestic life^(38)^, a perception that was shared by the study participants.

Students are not the only ones that are violent with lecturer. There are cases in which parents and co-workers are as aggressive or more aggressive than students. This type of violence is common, and previous history shows it is a predictive factor for the recurrence of this negative experience, which can occur every four to six months^(38-41)^.

Each teacher experiences violence in a different way. However, a study^(38)^ showed that obscene remarks were the most common type of violence, representing 34.36% of the identified episodes of teacher-directed violence.

It is important to emphasize that lecturer are not always aware that the way they are treated by students can be considered violence. This is categorized by Bourdieu as symbolic violence, a type of violence that is present in several social institutions, often as a subtle and recurrent phenomenon, commonly used by the ruling class to legitimize beliefs, behaviors, or even traditions^(32)^. Perhaps for this reason some of the lecturer interviewed believe that the triggers that drive student violence are the teacher’s own actions.

A study proposed by Moon and McClusey^(36)^ showed that lecturer who do not establish a friendly relationship with students are more likely to experience violence. For a good educational system, lecturer must be protected by the institution where they work^(38)^, because the more violence they experience, the greater the chances of emotional suffering in the future^(40-41)^.

### Limitations of the Study

One of the limitations of the study is the low adherence of professors to participate and share their experiences with violence, possibly because it is a sensitive topic. A total of 40 professionals were invited, and only 11 participated. In addition, the inclusion of other important social actors in this context, such as students and college administrators, would provide a deeper understanding of the theme, which shows the need for future research.

### Contributions to the area of health and nursing

By revealing the phenomena that are involved in and that trigger violence against lecturer during their professional practice, this study brings contributions that can support the development of strategies for preventing and/or coping with this problem at an individual level, aiming to change the behavior of lecturer and students, or in the sphere of management of educational institutions and even public policies, which can embrace these demands that need to be discussed and solved.

## FINAL CONSIDERATIONS

The factors that lead professors who work in undergraduate nursing courses and *lato* and *strictu sensu* postgraduate programs in health sciences, public health, and health education to experience violence in Higher Education Institutions in Brazil are characterized by institutional culture, gender, professors’ perception of violence and the triggers that drive students to commit violence.

Analyzing violence under Bourdieu’s theory, it is clear that student violence towards lecturer and the reports contained in this study deserve pedagogical reflection, understanding that social status and inequalities lead to positions of domination and, consequently, create a fertile ground for violence. However, in addition to the multiple forms of violence experienced and reported, it is necessary to include these discussions as a background in teaching environments, even though this does not guarantee that violence will not occur.

Promoting moments of reflection and discussing the theme with the managers of training institutions are strategies that can help professors of health courses and others to develop actions that will promote skills and attitudes to deal with violence, recognizing it and intervening accordingly, in order to prevent aggressive behavior from spreading, causing illness, and weakening interpersonal relationships in the academic environment.

In addition, the study showed that violence against professors in higher education institutions is directly associated with several aspects related to the students. In this direction, new studies should be carried out to investigate this theme from the perspective of students, aiming to understand this phenomenon and elucidate the meanings of the practice of violence against university professors.

Future studies should be carried out with participants other than nursing course professors, such as students and professionals from technical administration, general services, coordination, and management of universities, aiming to investigate the topic of violence from the perspective of these other important actors in the academic environment and obtain a richer and more detailed discussion and understanding about this phenomenon from different perspectives. The exploration of the theme in other courses in the area of health sciences, human sciences, applied, social sciences, and exact and biological sciences is also important for the analysis of the manifestation of violence in different contexts.

## COMPLEMENTARY MATERIAL

https://doi.org/10.48331/scielodata.VDMIT6

0034-7167-reben-76-01-e20210865-sup01
