# Supplementary material for: Tangency and multiple factors of violence against lecturer: nuances of the experience in pedagogical practices in health education
Source: Rev Bras Enferm. 2022 Dec 16;76(1):e20210865. doi: 10.1590/0034-7167-2021-0865 (PMC9749771; doi:10.1590/0034-7167-2021-0865)

BANCO DE DADOS ÂNGELA

PARTICIPANTE 1

Tempo de gravação: 00:14:41

Entrevistadora: Hoje são dia 12/08, as dez horas da manhã. Professora a senhora concorda, aceita participar da entrevista, a gente pode começar?

Entrevistada: Sim, concordo, podemos começar.

Entrevistadora: Hum, hum. Professora, se a senhora quiser ligar a câmera pode ficar à vontade, tá, a senhora preferir. Bom, eu vou começar aqui então. Professora, quais as iniciais do seu nome, por favor?

Entrevistada: Iniciais?

Entrevistadora: Isso, quais as iniciais do seu nome? É o L de Lucimeire.

Entrevistada: É LF, LFL.

Entrevistadora: LFL, obrigada. Qual que é a idade da senhora professora em anos?

Entrevistada: Quatro sete.

Entrevistadora: Quatro sete, sexo feminino. Nacionalidade da **Entrevistada:** Casada.

Entrevistadora: Qual que é a maior titulação da senhora professora, a senhora tem o senhora professora?

Entrevistada: Brasileira.

Entrevistadora: Brasileira. O seu estado civil?

doutorado, o doutorado?

Entrevistada: Sim.

Entrevistadora: Há quantos anos a senhora ensina no ensino superior professora?

Entrevistada: Desde de 98.

Entrevistadora: Desde de 98. Professora, para a senhora qual que é o conceito de violência, a palavra "violência", como que você me define ela?

Entrevistada: Violência, violência é você fazer algo com alguém sem que isso seja permitido.

Entrevistadora: Ok. Para você professora o que leva alguém a cometer um ato de violência?

Entrevistada: Um ato de violência, eu acho que são vários fatores, eu acho que um ato de violência ele pode ser cometido pela questão da certeza da impunidade, pela pessoa não obedecer aos critérios éticos e morais que existem, né, um acordo da sociedade,

porque cada sociedade tem os seus acordos éticos morais, né, que torna legal ou não, então a não observância desses acordos que é preestabelecido pela sociedade implica em um ato de violência, eu acredito que seja isso, na verdade, são vários fatores.

Entrevistadora: Entendi, professora. Alguma vez professora enquanto docente no ensino superior a senhora já vivenciou algum tipo de violência?

Entrevistada: Não que eu me recorde, não, eu acho que não.

Entrevistadora: No entendimento da senhora professora durante a formação dos profissionais da saúde você acha que o tema violência contra o professor ele tem sido trabalhado?

Entrevistada: Não, nunca foi trabalhado, pelo menos na minha experiência profissional a gente nunca trabalhou esse tipo de tema, né, relacionado à docência, né, violência na docência, nunca vivenciei essa experiência.

Entrevistadora: Ok. E na opinião da senhora professora, o que você considera violência contra o professor, você me falou antes o conceito de violência, né, a palavra isolada, e agora eu queria saber para você o que seria a violência contra o professor?

Entrevistada: Eu acho que assim, igual eu te falei, né, na questão dos acordos, então em sala de aula também existem acordos que são feitos, né, que podem ser feitos verbais, né, acordos verbais entre aluno e professor, ou mesmo que é considerado normais sociais, então eu acredito assim, que o não, não cumprimento dessas normas, dessas leis que são explicitas ou implícitas que seria uma violência, por exemplo, o professor está falando, está explicando, está dando a aula e o aluno ele não permite que o professor dê a aula, fica só conversando, atrapalhando os outros professores, no meu modo de pensar esse é um tipo de violência, não precisa ele agredir ou xingar o professor para ser considerado violência, eu acho que isso uma falta de educação em relação a figura do professor que está fazendo seu trabalho que já pode ser considerado uma situação de violência, porque esse aluno ele desrespeitou as normas que são impostas para aquele ambiente, então eu acredito que possa sim ser considerado um modo de violência.

Entrevistadora: Certo. Professora, enquanto docente alguma vez assim, na sua prática enquanto professora você trabalhou uma temática que visasse o respeito de toda a comunidade acadêmica, respeito aos professores, aos servidores, essa temática já foi abordada enquanto docente?

Entrevistada: Especificamente essa temática assim **não foi abordada**, mas eu já trabalhei com a disciplina de ética, e na disciplina de ética a gente fala um pouco essas questões do comportamento moral, do que é esperado do indivíduo na sociedade, então do posicionamento do indivíduo, um comportamento ético, político, um comportamento que o enfermeiro deve ter perante a sociedade, o que eu falo é formação do enfermeiro, né, então comportamentos éticos, não especificamente voltado pela questão assim da violência em sala de aula ou na universidade, né, então a gente trabalha a questão do que é esperado, qual que é o comportamento que o profissional deve ter relacionado a questão da ética e a questão da ética profissional.

Entrevistadora: Ok, professora. A gente já está finalizando, falta quatro perguntas. E eu queria saber para você professora o que você acha que leva um aluno a cometer a violência contra o professor, quais são os motivos assim, o que você acha que leva ele a cometer essa violência contra o docente?

Entrevistada: Não sei viu Sara.

Entrevistadora: Os motivos a senhora não consegue imaginar nenhum motivo que leve o aluno...

Entrevistada: Às vezes eles já traz essa violência de casa, às vezes ele passa por situações que são violentas e ele precisa descarregar, digamos assim, descarregar em alguém, então às vezes ele já passa por situações de violência no seu cotidiano, no seu dia a dia, né, sei lá, no transporte, no seu dia a dia, em casa, situações que possam ser consideradas violentas e ele nada mais do que reproduz esse comportamento, e acaba também agindo com violência não só o professor, mas às vezes com colegas também, eu acredito que talvez possa ser isso.

Entrevistadora: Ok, professora. Alguma vez a senhora já presenciou algum ato de violência de aluno contra professor?

Entrevistada: Violência de aluno contra professor?

Entrevistadora: É, a senhora já presenciou alguma vez?

Entrevistada: Não.

Entrevistadora: Não, ok. E porque a pergunta que eu tinha aqui, é o que mais te impressionou, então se a senhora não vivenciou a senhora não consegue recordar de nada que possa ter te impressionado em relação a essa temática?

Entrevistada: Espera só um pouquinho, deixa eu pensar aqui.

Entrevistadora: [Riso] Sem problemas professora.

Entrevistada: Eu acho que não, pelo menos assim eu não me recordo, Sara, pode até ser que tenha acontecido, mas que assim, no momento eu não me recordo.

Entrevistadora: Certo, professora. Professora quais... Para você quais seriam as possíveis estratégias para que fosse possível enfrentar essa questão de violência do aluno contra o professor, quais são as estratégias de enfrentamento para que não acontecesse essa violência e aluno contra o docente?

Entrevistada: Acredito que deva ser trabalhado isso de maneira institucional, que a gente, né, enquanto docentes a gente tenha o apoio da instituição, instituição não só da unidade acadêmica, mas dá própria universidade que seja trabalhado essa questão, esse conceito e essa temática, que seja transversal, e que a gente possa ter mais discussões sobre o tema, que através então dessa discussão e o apoio da instituição que a gente em conjunto possa descobrir maneiras de se trabalhar então preventivamente essa questão da violência.

Entrevistadora: Ok, professora. E por último, eu gostaria de saber se a senhora tem mais alguma coisa para comentar sobre essa temática, se gostaria de falar mais alguma coisa sobre a violência contra o professor?

Entrevistada: Ah, eu acho assim que a principal violência contra o professor ela acontece a partir do momento em que o professor ele não e valorizado no seu ambiente de trabalho, não só em relação à questão aluno/professor, mas até mesmo em instâncias superiores, pela não valorização, né, na questão salarial, a questão de falta de recursos didáticos que a gente enfrenta, o apoio do próprio órgão máximo que seria o Ministério da Educação que apoiasse e valorizasse esses professores e instituísse políticas que fossem mais efetivas, e que já fosse assim implantada essa questão, né, da valorização e não violência em relação ao professor, porque o que a gente ver aí é assim, quando a gente deseja alguma coisa a gente precisar fazer greve, parar as atividades prejudicando muitas vezes a nós mesmos e aos alunos, né, porque nós não temos um salário que seja digno para que possamos manter as nossas famílias, então muitas vezes a gente precisa de ter mais de um emprego, e isso aí é muito cansativo, porque assim, a gente não tem aquele tempo para poder dedicar da forma adequada como deveria ser, e isso na universidade federal é um pouco diferente do que nas instituições particulares, porque na universidade federal a gente ainda tem a figura do professor que tem uma dedicação exclusiva, e essa dedicação não é remunerada, né, para que ele possa então se dedicar exclusivamente a isso, mas mesmo assim eu não considero que essa dedicação exclusiva que ela seja devidamente remunerada de acordo para que o professor consiga se manter com um vínculo só, isso comparado com outras profissões, tá, que a gente observa que às vezes o professor ele tem um investimento muito maior, que ele tem que estar sempre estudando, sempre se atualizando, e diferente de juízes, por exemplo, que tem o auxílio livro, né, para ele está se capacitando e estar lendo sempre, e se atualizando, o professor ele não tem, então toda a capacitação que ele tem, ele tem que tirar do bolso, ele tem que fazer um investimento próprio, recursos didáticos, recursos materiais se ele quiser uma coisa diferente ele que tem que investir, não há um investimento do Governo Federal para que isso aconteça, então eu acho que é muito isso, sabe. As iniciativas elas são próprias, elas são individuais, não existe da parte do Governo Federal e da instituição esse tipo de envolvimento, então eu acredito que seja isso, a questão principal é da valorização dos profissionais da docência, tá, que são os profissionais que vão capacitar e vão formar outros profissionais, médico, juízes, né, todos passam por um banco acadêmico para poder se formar, e depois a gente ver é uma dispare de tratar então, né, pessoas que tem o mesmo tipo de titularização e de maneira tão diferente, né, recebem os seus salários de maneira tão diversa e tão diferente, porque eu acredito que isso demostra a questão da valorização do profissional, né, então eu acho que é isso viu Sara, que a principal questão é essa.

Entrevistadora: Certinho professora, muito obrigada novamente pela senhora ter disponibilizado o seu tempo, desculpa a minha voz, hoje eu acordei bem roca, muito obrigada por estar participando, né, é um estudo muito relevante. Eu vou encerrar a nossa gravação agora e uma boa semana para a senhora e um bom restinho da manhã, muito obrigada.

Entrevistada: Obrigada também.

00:14:41

PARTICIPANTE 2

Tempo de gravação: 00:28:41

Entrevistada: Tenho o Pedro, mas eu também tenho o Miguel que tem três anos e aí divido o cuidado deles com meu marido, mas meu marido também está no home office, então está uma loucura aqui, sabe [Risos], desculpa a demora, eu costumo sempre colaborar, porque eu sei o tanto que esse trem é difícil.

Entrevistadora: Difícil. Então, professora Natália, deve ter lido já lá o TCLE, né, você chegou e ver no Google Forms?

Entrevistada: Eu queria até saber se é preciso preencher lá e te encaminhar?

Entrevistadora: Então, não, assim, aqui a gente pode alinhar só você dizendo para mim que você concorda, porque nesse momento pandêmico não há necessidade de você devolver escrito porque a gente grava e aí eu preciso só que você concorde, isso é o suficiente.

Entrevistada: Concordo. Estava indecisa, assim, se precisava preencher ali também, mas tudo bem, eu concordo.

Entrevistadora: Ok. Então, professora, vamos iniciar, eu não vou tomar muito o seu tempo, ela é uma entrevista que vai levar em torno... É uma entrevista semiestruturada, tem uma previsão de quinze a vinte minutos, com a temática de Violência no Ensino, assim, a Eurides que intermediou, então eu já agradeço a sua disponibilidade, e aí gostaria de iniciar com as perguntas fechadas daquelas que a gente já trabalha há algum tempo, qual é a sua idade em anos, professora Natália?

Entrevistada: Em anos trinta e três.

Entrevistadora: E a sua nacionalidade?

Entrevistada: Brasileira.

Entrevistadora: O estado civil?

Entrevistada: Casada.

Entrevistadora: A maior graduação de escolaridade?

Entrevistada: Doutorado.

Entrevistadora: Doutorado. E agora, há quanto tempo você exerce à docência, é professora?

Entrevistada: Quanto tempo eu sou professora, deixa eu ver, desde dois mil e doze, dois mil e doze, dois mil e doze, a gente está em dois mil e vinte, oito anos.

Entrevistadora: Oito anos, muito bem. Professora, qual que é o seu entendimento ou conceito de violência?

Entrevistada: Nossa, [Risos] que pergunta, aí, aí. Bom, eu acho que para mim violência remete a agressão, né, agressão, e aí... Aos meus valores eu acho que tudo que me desrespeita, eu acho tem a ver com desrespeito, com agressão, com desrespeito, para mim violência tem a ver com isso.

Entrevistadora: Ok. E você consegue elencar alguns fatores que desencadeiam a violência, que são desencadeadores da violência?

Entrevistada: Olha, eu acho, a partir disso que eu estava falando eu acho que o desrespeito eu acho que é um fator, quando não respeita os valores do outro, né, ou quando não tem... também um outro fator a falta de escuta, independente de... para tentar compreender a pessoa ou a situação, bom, essas duas coisas me vêm à cabeça agora, a falta de uma escuta e consequentemente o desrespeito.

Entrevistadora: Ok. Você já sofreu algum tipo de violência durante a sua prática pedagógica desencadeada por alunos? Sim, e que tipo?

Entrevistada: Eu já sofri numa época que eu trabalhava numa instituição particular, e para mim foi uma violência muito grande porque sempre ministrei disciplinas que são disciplinas, vamos dizer que são disciplinas que não tem muita reprovação, mas um aluno por falta, por desempenho, essas coisas, eu acabei reprovando um aluno na disciplina, e quando eu retornei no semestre passado esse aluno estava na minha disciplina e eu falei, mas era pré-requisito, eu reprovei e tal, depois eu fui descobrir que a coordenação aprovou o aluno, também não sei, eu acho que para a lei devia ser até um crime, né, mas eu me senti muito violentada, muito violentada mesmo, nesse aspecto, e aí a justificativa era que, porque olha, a gente... era pouco que faltava e tal, a gente aprovou essa pessoa, até porque a gente precisa ter uma certa quantidade de alunos, do ponto de vista financeiro mesmo da instituição, sabe, de permanência dos alunos, então foi isso, então fiquei bem chateada com isso, e aí encontrei o aluno do semestre passado e ainda tinha um deboche, reprova, mas estou aqui de volta, né, então acho que essa foi a mais grave nesse ponto... Desculpa, é que meu marido passou aqui, eu acho que essa foi a principal situação relacionada a instituição. Dos alunos eu... sinceramente eu nunca me senti desrespeitada, né, acho que não, não me vem na cabeça agora, Gilda... É Ângela Gilda, né?

Entrevistadora: Ângela Gilda, é, tudo bem.

Entrevistada: Se eu lembrar de novo eu falo contigo.

Entrevistadora: E a sua atitude diante dessa violência foi como?

Entrevistada: Bom, eu questionei a coordenação e a direção do curso, eles tentaram justificar e aí eu não levei à frente mais além da questão e da chateação que eu tive. Bom, eu acho que eu era bem imatura, foi meu primeiro ano enquanto docente, estava aprendendo muitas coisas, talvez em outro momento eu teria tentado verificar outras questões porque eu entendi que, para além, de uma violência foi um crime mesmo, me senti completamente desrespeitada, então eu não segui para a frente com isso, até porque tinha vinculação, era meu emprego, eu tinha [...] e aí era aquela história, olha, não tem, para mim, assim, você, se não tiver, a gente arruma outro professor e está tudo certo e aí você fica sem esse emprego, hoje isso me causa uma ojeriza que você

não tem noção, mas foi imaturidade e tinha essa dependência também do emprego, e não levei a frente.

Entrevistadora: Em seu entendimento, professora Natália, durante a formação dos profissionais da saúde essa temática violência contra professores está contemplada nos conteúdos mínimos nos projetos pedagógicos dos cursos?

Entrevistada: Não, eu entendo que não, a questão da violência contra o professor, eu não... eu confesso que eu não sou uma estudiosa do projeto pedagógico, mas sempre tive o cuidado de ler o projeto pedagógico em todas as instituições que eu passei, e eu nunca percebi isso, inclusive quando você foi me fazer, eu vi o tema eu falei, nossa, eu fiquei curiosa, será que pergunta que vem, né, será que eu vou conseguir responder? Mas eu não vi contemplado, eu já vi esse tipo de discussão, quando eu entrei na UFG e aí a UFG tem um curso, a gente brinca que é o curso de noivos, para casar com a UFG, mas que não é necessariamente um curso de professores, a gente precisa fazer esse curso para passar o estágio probatório, e essa temática foi abordada, mas não do ponto de vista do cuidado com o profissional, mas, assim, quais são as possibilidades, o que e que está contido institucionalmente para isso, como é que a gente pode evitar problemas com relação a aluno em função da violência, muito ampassã, mas isso foi abordado, lá nesse curso, mas não me lembro de verificar isso no projeto pedagógico.

Entrevistadora: Perfeito. Na sua opinião o que você considera violência contra o professor?

Entrevistada: Eu acho que é quando... Existem várias formas, com relação a aluno eu acho que a própria, uma atitude agressiva na forma de ser questionado, né, porque eu acho que o aluno tem o direito de questionar o professor, claro, mas a depender de como essa forma é feita ela pode ser muito violenta, eu não falo nem de uma questão de agressão física, mas de uma questão de agressão com relação à proposta pedagógica da disciplina mesmo, né, vai depender de como a forma que esse aluno faz que eu acho que isso é considerado uma violência. A violência física nem se fala, eu acho que isso pode vir a acontecer e a gente vê em outros espaços, na universidade nem tanto mais isso acontecer, algumas questões com relação aos princípios mesmo do professor e aí tem a ver com... como fala? Desculpa, minha cabeça ainda está de grávida, [Risos]. Bom, a gente em várias áreas, em especial a minha, a gente tem correntes teóricas, muitas vezes divergentes, elas são contraditórias até, e a depender da formação do professor ele pode escolher uma corrente ou outra, e aí isso pode ser o questionamento de muitas vezes os alunos isso pode ser de forma bem violenta, eu já passei por alguma coisa, lembrei disso agora, o aluno questiona a partir de uma outra corrente teórica e aí eu valido, olha, isso e um dado, né, isso existe, mas, a princípio a gente defende isso por causa disso e disso, e aí eu fui muito... fui questionada de uma forma bem agressiva e aí o aluno utilizou algumas palavras, como, ah, essas pessoas idealistas demais, isso é coisa de lunático, eu lembro de ele dizer isso, coisa de lunático, deixa eu ver... Você perguntou as formas, né, de violência?

Entrevistadora: Isso. O que você considera.

Entrevistada: O que eu considero. Estou pensando aqui. Ângela, 12 eu acho que assim, para mim acho que eu resumi isso, né, quando você é questionado o ponto de vista teórico, metodológico, pedagógico de uma forma desrespeitosa, eu não sou

contrária ao questionamento, mas isso é de uma forma desrespeitosa, tenho sempre o costume de apresentar e discutir, gastar um tempo enorme no primeiro dia de aula da proposta de disciplina, da proposta metodológica, teórica e tal, e deixar bem aberto para os alunos, aí lá no meio do semestre alguém quer questionar, beleza, eu ainda dou um espaço, mas a forma com que isso é feito às vezes pode ser de forma violenta.

Entrevistadora: Professora Natalia, nesse sentido e assim linkando aí a pergunta, você desenvolve alguma experiência na prática docente que vise o respeito a toda a comunidade *acadêmica?*

***Entrevistada**: Sim, sim*, eu ministro na faculdade de enfermagem hoje, e sempre eu ministrei, tá, na prática docente, e isso independente da disciplina, mas sempre ministrei também essa disciplina que é saúde mental, então eu sempre trouxe, tenho como o princípio as relações humanas, né, e a relação interpessoal humanizada, escuta qualificada, e aí sempre discuto que isso deve perpassar em qualquer tipo de relação, seja enfermeira ou paciente, seja com a comunidade, seja com populações vulneráveis, né, porque às vezes a gente tem um desrespeito muito grande, com isso seja na relação professor/aluno, porque eu também percebo que a violência ela pode acontecer, e aí a gente já viu isso tudo daqui para lá, né, da minha parte enquanto professor docente para com o aluno, mas ao discutir isso eu também discuto que eu exijo, né, e aí me coloco isso que o respeito também tem que vir dele, e às vezes os alunos eles têm uma dificuldade, não sei o que está acontecendo, mas os alunos chegam com uma dificuldade de fazer isso, né, de como é que eu faço um questionamento, uma preposição, alguma coisa de uma forma que não seja pessoal ou... Eu até brinco, deselegante com eles, né, e aí fala tudo bem, você pode dizer isso, mas vamos fazer isso de outra forma, né, eu te dou oportunidade de você rever a forma como você está falando comigo, né, e aí todo mundo respira, né, isso aconteceu várias vezes, eu não entendi naquele momento necessariamente como uma violência, mas como uma imaturidade mesmo que a pessoa teve, ou que o aluno teve para estabelecer as relações, inclusive comigo enquanto docente. Mas essa questão das relações, do respeito nas relações de uma certa forma ela está de forma transversal em todas as minhas disciplinas.

Entrevistadora: Ótimo. E o que você acredita ou observa que leve um aluno a usar de violência contra o professor durante a prática pedagógica dele? Não necessariamente na sua, mas assim, que entendimento você tem que esse aluno, né, leve esse aluno a cometer violência, a praticar?

Entrevistada: Eu acho que tem, a gente não pode desconsiderar a história de vida, o contexto de vida do aluno, né, às vezes ele já tem na sua história uma vivência de uma relação muito violenta, seja com os pais, seja com os colegas, seja nas instituições de ensino por onde ele passou, então eu acho que uma experiência anterior acaba influenciando muito e fazendo com que ele acaba reproduzindo inconscientemente aquela violência, né, eu acho que isso é uma questão. Acho que tem a questão também da maturidade, porque esses meninos estão chegando muito novos assim, às vezes eu entro para discutir algumas coisas, eles estão com carinha de, de... aí espera aí, de adolescentes assim, carinhas, meninos tudo novo e tal, e às vezes a gente vai discutir questões tão importantes, profundas, que exige uma vivência, uma maturidade, e é desafiadora, então eu acho que a maturidade deles também é complicada, e eu também

acredito que exista uma... às vezes pode ser uma reação a própria atitude do professor, tá, então, assim, eu não quero, mas assim, a gente sabe que tem alguns colegas que tem uma atitude violenta também, né, com relação ao aluno, e a reação dele não justifica, mas a reação dele também pode ser violenta, a falta de diálogo, a falta de espaço para que esse aluno questione, se posicione, a postura às vezes do professor de estar, de que eu sei tudo, e aí fica na sua que você precisa aprender, às vezes é complicado, não estou querendo justificar a violência do aluno, acho que isso não justifica, mas às vezes eu percebo isso não só na minha prática, mas próximo a outros colegas, né, a falta de conhecimento também de estratégias para lidar com alguma situação conflituosa na relação com o professor, e aí desde de estratégias de manejos de relação interpessoal até institucionais mesmo, né, eles às vezes desconhecem isso, eu já vi, já acompanhei uma situação não na unidade que eu estou, mas uma reação muito violenta do aluno, tinha algumas, mas não justificava, mas a reação violenta, e aí desconhecia os mecanismos para denúncia ou para acompanhamento da situação, por exemplo, ouvidoria, sabe, coisas do tipo, mas eu acho que tem tudo isso, assim, eu acho que deve ser bem complexo, né, depois eu quero descobrir aí com o seu trabalho [Risos].

Entrevistadora: Então professora Natalia, você citou aí agora que você já acompanhou, é a minha próxima pergunta, né, e você assim, sem saber linkou aqui com a pergunta, né. Que é, até hoje durante a sua prática ou não, né, o que mais te impressionou sobre violência contra o professor? Aí você disse, olha, eu já tive oportunidade e acompanhar, aí que eu vi o linke, que eu notei o linke, você gostaria de comentar?

Entrevistada: Eu gostaria, então, isso foi uma situação numa outra unidade, o aluno questionou alguma coisa do... a uma postura do professor, professor foi bem irredutível com a situação, bom, o meu julgamento não tem jeito de eu julgar, o meu julgamento eu acho que ele foi irredutível, que poderia ter revisto a situação, mas ele estava no direito dele também, ele não estava fazendo nada de errado, a situação foi olha, você não vai entrar na sua sala agora porque você está vinte minutos atrasado, fechou a porta não entra mais, bom, ele tem o direito de fazer isso, a gente sabe, o professor tem essa autonomia ele tem o direito de fazer isso, mas para que, tudo bem, a aluna ficou muito revoltada, e aí em função disso entrou na sala, fez uma agressão verbal em importante assim, né, outros alunos fizeram corro com relação ao professor e tal, então eu não sei até que ponto esses alunos, essa turma já vinha sendo violentada pelo professor, mas a situação foi que eles fizeram e o professor ficou bem acuado e isso foi parar na coordenação e tal, e a coordenação do curso, pelo menos a instituição que eu tenho não tem que lidar com esse tipo de coisa, tem tantas outras coisas, não é atribuição, e aí dessa turma eu acompanhei, tinha um completo desconhecimento na situação de ouvidoria, dos mecanismos para poder fazer os questionamentos, dos processos avaliativos, tanto do docente, inclusive, e tal, e o professor se percebeu também numa situação completamente desprotegido, né, porque ele, de certa forma tem o direito de fazer isso, mas quando fez qual que é o respaldo institucional que ele tem para determinadas situações? Então, assim, acho que foi uma violência, eu percebi, de duas partes, então isso, fiquei muito impressionada, né...

Entrevistadora: Ótimo. E o que você, assim, vê como possíveis estratégias de enfrentamento a violência dos alunos contra o professor?

Entrevistada: Acho que uma das principais estratégias é a gente ter oportunidade de em algum momento do curso discutir isso, não com esse tema, mas eu acho que das relações professor/aluno, e aí eu estou dizendo isso é uma via de mão dupla, assim, tanto de... como do professor para com o aluno, como do aluno para com o professor. Acho que essa... e isso não estou dizendo de uma palestra, e tal, talvez alguma estratégia didática, criativa, para proporcionar uma vivência de respeito mútuo, eu até vi ontem, ontem vi uma charge, assim, uma charge não, uma fala, um tweeter, que uma pessoa postou, falou, olha, colegas, tenham paciência com o seu professor nesse momento de aula remota, porque o meu pai é professor e ele me liga todos os dias ansioso tentando testar, preparando as aulas e testando o aplicativo para ver se vai funcionar, porque ele fica muito nervoso, não sabe se vai dar conta, então, assim, a gente tem os nossos medos, as nossas dificuldades, assim como o aluno tem, mas eu não sei se é porque isso foi colocado e o professor suprassumo e tal, e aí os alunos têm essa dificuldade de chegar e às vezes quando chega é de forma tão violenta, ou se a gente ainda fica assumindo esse lugar, eu não gosto de ficar assumindo esse lugar, de que eu sei tudo, e é isso, de que não erram e é isso mesmo, então acho que fugiu um pouco, mas o que eu queria dizer é que, assim, essa relação interpessoal ela precisa ser cuidada, os lugares, como é que a gente... eu acredito que a hierarquia nesse aspecto ela é importante, mas como que a gente faz isso de uma forma que nos seja desrespeitosa, né? Que a gente considere as habilidades um do outro e as dificuldades, também, um do outro, como uma possibilidade de construção e não de diminuição da habilidade ou da capacidade do professor, né, algumas coisas assim. Então, eu acho que isso devia ter em algum momento do curso uma atenção para isso, seja de forma transversal, seja em alguma disciplina específica, e quando você me traz esse tema eu fico bem curiosa, acho que isso deveria ser abordado inclusive em saúde mental, né, acho que teria que estudar um pouco o que você está estudando aí para entender.

Entrevistadora: Então, Natália, eu estou na última pergunta da nossa entrevista e eu deixo sempre, nesse momento, caso o professor que eu estou entrevistando queira falar algo sobre a violência contra professor, então, nesse momento, eu deixo você à vontade, às vezes algo que a pergunta não está tão redondinha ou que você gostaria de complementar. Então, a minha pergunta é essa, você gostaria de complementar essa temática, violência contra o professor?

Entrevistada: Eu acho que eu queria complementar, na verdade, só ressaltar um pouco do que eu comecei falando que é a questão da violência que ela ocorre institucionalmente, e não por parte só das relações professor/aluno, sabe? Muitas vezes essa violência ela parte do colega coordenador, do colega de disciplina, eu acho que essa questão de violência institucional ela é adoecedora, e eu confesso que eu percebo isso muito mais, na minha prática profissional muito mais do que na relação professor/aluno, numa violência institucional, sabe? Eu exemplifiquei uma situação em uma unidade, num lugar, mas eu percebo que às vezes as demandas, a falta de apoio institucional, inclusive no contexto das universidades federais também, né, porque a gente não está só na sala de aula, a gente faz pesquisa, a gente faz extensão, e nesses espaços também é violentado, eu queria exemplificar, por exemplo, a situação de um professor pesquisador agora nesse contexto da pandemia que a pesquisa dele estava sendo utilizada pela secretaria de saúde de estado e de repente teve a cara, a imagem, o currículo circulando nas redes sociais e o pessoal, as pessoas criticando, né, que é o

Rangel, o Tiago Rangel, e aí eu fiquei pensando, cara, ele está assim, sofrendo, a moral dele está circulando aí nas redes sociais, e como é que a instituição apoia, ou cuida, ou dá um respaldo para as coisas que a gente faz, eu acho que isso também, o acúmulo de atividades acho que às vezes vem como uma violência institucional também, dependendo da forma como isso é feito, e percebo, também, assédio moral, institucionalmente a gente, muitas vezes é assediado, tem um assédio moral mesmo na instituição. Eu acho que é um pouco disso, eu não sei se entra na sua discussão, mas para além da relação professor/aluno é uma violência institucional importante que precisa ser questionada.

Entrevistadora: Então, Natália, eu sou doutoranda lá da FEN, com o eu citei no início, a minha temática é a "Violência Contra o Professor na Prática Pedagógica em Saúde", uma abordagem intercultural, é uma temática ainda iniciante nessa coleta, é muito importante ter aqui a sua entrevista e contribuição, à medida que eu finalizar o trabalho vou ter muito prazer em fazer essa devolutiva uma vez em que instigou em você essa curiosidade, e isso para um pesquisador é, assim, um bálsamo. Então, quero agradecer, Natália, muito obrigada, viu, muito obrigada de verdade.

Entrevistada: Eu espero ter contribuído, porque é tão diferente, entendeu, olha, eu não sei, não sei se eu estou contribuindo, se eu estou falando alhos com bugalhos, mas espero ter contribuído, mas eu fiquei muito curiosa, assim, para ver o que é que tem rolado por aí sobre essas questões.

Entrevistadora: Ok, então, obrigada viu.

Entrevistada: Não só professor, na prática na área de saúde, né?

Entrevistadora: Na prática na área de saúde.

Entrevistada: [...] Talvez você precisasse de alguém, mas ele é das exatas, então... [Risos].

Entrevistadora: Na prática em saúde, mesmo, a gente analisou por aqui.

Entrevistada: Bacana, bacana.

Entrevistadora: Um, beijo para você, um beijo para o Pedro, viu? Para o outro bebê, muito obrigada mesmo, tá bem, um grande beijo professora.

Entrevistada: Um beijo, obrigadão, bom trabalho aí Ângela.

Entrevistadora: Obrigada, obrigada, tchauzinho.

00:28:41

PARTICIPANTE 3

Tempo de gravação: 00:28:12

Entrevistadora: Então muito obrigada, viu professor. E aí assim, nesse momento aqui, eu não vou me estender muito, ela é uma entrevista, ela vai durar aproximadamente entre quinze a vinte minutos.

Entrevistado: Hum, hum.

Entrevistadora: Para que a gente possa, né, estar nesse diálogo, e aí eu gostaria primeiro de perguntar assim, as iniciais do seu nome, a ideia em número, e a graduação, a titulação maior, por favor, professor.

Entrevistado: Eu sou Enfermeiro, nasci em 15/08/68, as letras do nome DJN.

Entrevistadora: Ok. E quanto tempo que você é professor?

Entrevistado: Nossa Ângela já tem tempo viu.

Entrevistadora: É mesmo [Risos].

Entrevistado: Eu comecei na verdade aulas quando eu fazia faculdade ainda, então quando eu fazia faculdade eu já comecei a dar aula no ensino técnico, naquela época o conceito não era tão efetivo em relação a cobrança de títulos, não era graduado, mas recebi o convite e fui dar aula no Oswaldo Cruz que é uma escola que até hoje existe, era 1994 mais ou menos, então já tem um chão já, né.

Entrevistadora: Ok. Professor, passou desapercebido da minha leitura. Qual é o seu estado civil, por favor.

Entrevistado: Casado.

Entrevistadora: Casado, ok. Então, professor, qual é o conceito que você faz sobre violência, você tem um conceito formado, o que você fala para mim se fosse para conceituar a violência?

Entrevistado: Oh, se fosse para conceituar a violência, violência é tudo aquilo que te agride, né, ou seja, que fere os seus princípios, que te humilha, que te joga para baixo, que causa desconforto, então eu acredito que violência é isso, né, algo que invade o seu espaço como ser humano e provoca algum tipo de desconforto, pode ser físico, pode ser psíquico, moral, ou seja, uma violência que pode chegar de diversas formas, eu acredito que a violência tem mais ou menos esse caráter.

Entrevistadora: Ok. Para você quais são os fatores que podem desencadear a violência, tem fatores que podem?

Entrevistado: Olha, a violência no contexto acadêmico, a violência de uma forma geral?

Entrevistadora: Então, no contexto acadêmico é o que tem a titulação, mas à princípio agora no contexto geral?

Entrevistado: Eu acho que no contexto geral o que faz com que a violência aconteça, né, e o que faz com que todos nós fiquemos vulneráveis a essa violência eu acho que é a desigualdade que existe em nosso país, então a desigualdade em relação à possibilidade de educação, a possibilidade de bens de consumo, possibilidade de ser reconhecido como cidadão, eu acho que tudo isso promove violência, então a questão de não termos também uma divisão em relação à economia, né, ou seja, recursos, ou seja, no Brasil a desigualdade social é uma coisa que incomoda, ou seja, é um grupo muito pequeno tem muito, e um grupo gigantesco que vive de migalhas, então eu acho

que tudo isso gera revolta, promove violência, então... O investimento precário em educação, em saúde, em segurança pública, é uma segurança pública extremamente hostil, agressiva, inadequada, principalmente em relação a parte da população que é mais vulnerável que são os pobres e negros, então eu acho que tudo isso gera violência, são geradores de violência, então são vários fatores que precisam ser trabalhados, e que infelizmente eles... Passa governo, entra governo e sai governo, a situação só piora, então infelizmente, eu acredito que tudo isso é gerador de violência.

Entrevistadora: Ok, ok. Você já sofreu professor algum tipo de violência durante a sua prática pedagógica, professor Douglas?

Entrevistado: Sim, em relação à violência o que eu observo na minha prática pedagógica, eu acredito que o aluno com qual nós trabalhamos hoje é um aluno muito diferente, muito diferente do aluno que nós fomos em algum lugar do passado, então eu acredito que essa questão geracional e esses conflitos em sala de aula ou em diferentes espaços eles acontecem, há também por questões que tem uma relação direta com essas diferenças geracionais, então... E a violência em sala de aula ela pode ser percebida de diferentes formas, aí então nós temos desde de a violência do aluno que é desrespeitoso em relação a não estar atento, mas não só não estar atento, tá, mas também perturbar o que, perturbar o momento da aula com o uso de celular, por exemplo, tá então toda vez que o aluno é inadequado também em relação entrar e sair da sala a todo o momento, então são situações que a gente percebe como uma certa hostilidade, visto que, alguns contratos são firmados no início do semestre, e quando o aluno cumpre, quando o aluno é muito inadequado em relação a sua postura em sala de aula chegando atrapalhar esse processo de ensino/aprendizagem, eu percebo isso como uma violência.

Entrevistadora: Então, você diria que sim, você já sofreu um tipo de interferência [...] entre sim e não?

Entrevistado: Ângela nesse sentido sim, nesse sentido eu acho que essa inadequação e todo esse conflito que existe em algumas situações, né, eu acredito que isso é um pouco violento, inadequado, e isso incomoda, incomoda não só o professor, mas às vezes incomoda o restante do grupo também, então, por exemplo, quando você pede um aluno no ensino superior que raramente isso é bem improvável, mas infelizmente acontece ainda, quando o aluno incomoda a ponto de você pedir para o aluno sair da sala, isso gera um conflito muito grande entre professor e o aluno que estão envolvidos na situação, e você acaba dividindo o grupo também, porque vai ter aluno que concorda com atitude, vai ter aluno que discorda, e isso promove o que, promove um ambiente muito ruim, né, muito inadequado, então eu acredito que de certa forma nós também temos responsabilidade nesse contexto de violência, né, dependendo da forma como nós trabalhamos com esse aluno, então eu acho que a violência é uma via de mão dupla. Assim, não conheço o seu trabalho, mas a violência ela tem essa característica também, ou seja, o professor no contexto em sala de aula tanto ele pode sofrer violência como ele pode praticar a violência de uma forma muito sutil, né.

Entrevistadora: Está ok. E fazendo esse link quando você diz olha, nesse sentido Ângela eu entendo que sim, né, seria física, moral, verbal, sexual, psicológica, emocional?

Entrevistado: Não eu acho que a violência, né, ela tem um aspecto não verbal muito forte, né, do aluno, então o aluno às vezes ele é muito sutil na sua violência, ele é muito sutil na sua postura corporal, que pode indicar, o que pode indicar descontentamento com alguma coisa nessa inter-relação, tá, mas pode indicar também o desejo de te agredir, o desejo de medir forças, então eu acredito que isso é muito comum, aí quando eu olho para os nossos alunos, em especial os alunos da FEN, né, eu acredito que a forma que eles são recebidos, né, e a forma que todo esse processo de ensino acontece ao longo dos anos, talvez dê para eles uma autonomia, tá e uma postura às vezes um pouco inadequado em relação ao professor, porque, um dia desses eu estava discutindo com um amigo essa questão do aluno nosso e do aluno de outros cursos da área da saúde e da UFG, né, a gente observa que, a gente observa que em outros cursos a relação do aluno com o professor é de muito mais cordialidade, de respeito no trato, né, então... Na FEN parece que isso se perde em algum momento da caminhada, então eu acredito que a gente precisa rever isso, porque até que ponto, quando você não se posiciona e quando você não coloca um limite, até que ponto isso é bom para o aluno, né, porque às vezes parece que fica, parece que é chato, é inadequado você falar, aqui a relação em algum momento precisa ser vertical, em algum momento ela precisa sim, ela precisa ser um pouco vertical, dá para ter uma relação horizontal com os alunos sem nenhuma dúvida, eu tenho feito muito isso na minha carreira, mas em algum momento o aluno precisa perceber que essa relação de respeito, essa hierarquia ela está presente na vida dele desde do nascimento, e vai estar presente até o fim dos dias dele, ou seja, na universidade a gente tem essa hierarquia, na família a gente tem essa hierarquia, no trabalho a gente tem essa hierarquia, e quando você não permite que o aluno perceba isso e respeite, da mesma forma que ele é respeitado em relação a esse aspecto, o aluno se perde durante a caminhada, ele se perde, e a culpa eu acredito que dessa violência em especial nos últimos anos de faculdade, e desse embate, né, que às vezes acontece entre professor e aluno, eu acredito que a FEN ou a faculdade de enfermagem em si tem muita culpa em tudo isso, né, por não se posicionar de forma adequada em relação a essa convivência, né, deixando claro que nós temos o que, nós temos uma hierarquia e tanto professor e aluno precisam ser respeitado dentro desses aspectos, então eu acredito que nós não criamos um ambiente muito saudável no aspecto relacional durante a caminhada pelo curso, então o aluno dependendo da forma que for trabalhado essas questões da inter-relação desde do princípio, que ele tenha uma chance de aprender muito e de aprender a se relacionar em diferentes espaços eu acho que nós estamos promovendo um desserviço em alguns aspectos em relação a essa convivência, e isso provoca, não deixa de provocar uma violência, uma violência que acontece no transcorrer do curso, e em especial quando o aluno está no último ano, então eu tenho observado isso, e isso é muito ruim.

Entrevistadora: E você tem trabalhado, na sua formação profissional assim essa temática violência com os seus alunos, em algum momento na sua disciplina trabalha?

Entrevistado: Oh, eu trabalho com esse tema com a frequência um pouco maior, porque eu trabalho na área de psiquiatria e na área de saúde mental, então é um tema que, por exemplo, na saúde mental ele brota, ele surge em várias discussões, por exemplo, a saúde mental é uma disciplina onde discussões ligadas ao aspecto relacional de aluno com aluno, de aluno com professor, de aluno com outros profissionais da área da saúde que ele tem contato especial no Hospital das Clínicas ou

em outros espaços, então essa questão da violência ela está muito presente nessas discussões, e a violência parece em muitos momentos, aí em muitos momentos o aluno ele se posiciona, ele reclama, ele se queixa de comportamentos violentos verbais ou não verbais, então isso infelizmente é muito comum, e como eu trabalho também com a questão de dependência química a violência, infelizmente, ela faz parte desse tecido, aí ligado a que, ligado a esse tema que é a dependência química, então a violência também ela aparece muito, está nas discussões quando eu trabalho com política de redução de danos e progressão, daí, então, a violência está sempre muito presente, então é um tema que surge muito, eu acredito também pela área, pela área que eu trabalho, então a violência é algo que está sempre muito presente.

Entrevistadora: E ela se estende além, essa forma de você trabalhar ela se estende além da sala de aula? Você trabalha essa temática em algum momento no meio acadêmico extra sala de aula?

Entrevistado: Não, Ângela...

Entrevistadora: Por exemplo, na sala dos professores, ou num grupo de pesquisa, na extensão?

Entrevistado: O tema violência ligado à saúde mental acaba sendo um tema muito transversal, então aqui em discussões em diferentes espaços pode aparecer, mas não é, o que, não é algo planejado, ah, vou trabalhar em determinado contexto com os professores em outro espaço ou em outra unidade acadêmica com esse tema, ah, mas como é algo que parece está muito presente no nosso dia a dia, no dia a dia das outras unidades acadêmicas, essa temática ela acaba aparecendo, ela acaba aparecendo, às vezes de uma forma muito sutil, às vezes de uma forma um pouco mais aberta, mas é um tema que está sempre muito presente, mas não de forma intencional, ou seja, não tem um projeto que fala sobre violência em diferentes espaços.

Entrevistadora: E a que você atribui que leve um aluno a cometer violência contra o professor?

Entrevistado: Eu acho que a violência impressa pelo aluno contra o professor o que leva é um conjunto de situações, eu acredito que a violência, em relação ao porque que ela surge é devido a vários fatores, ou seja, é multifatorial, às vezes a violência que chega ao professor ela não tem o professor como foco, mas de repente quem estava no caminho de uma pessoa que já está muito fragilizada, que já sofreu também uma infinidade de violências ao longo da caminhada, quem estava no caminho, de repente, num momento de explosão, num momento de explosão verbal ou de uma explosão tá que pode chegar até a via física mesmo é o professor, então a violência, Ângela, eu percebo a violência como algo muito ruim, mas eu percebo a violência também em algumas situações como um pedido de socorro, como um grito que de repente sai de forma atravessada, grosseira, ríspida, tal, mas que também chama muito a atenção, de repente está tudo de ruim que vem acontecendo nos últimos tempos e que ninguém percebeu ainda, então eu acho que perceber a violência só como algo ruim ligado a ausência de uma educação mais refinada parece que isso é tão elitista, né, assim, eu não concordo muito com isso, eu acho que quando você sofre alguma violência, independente de quem a pratica, mas pensando no aluno e tal, você precisa chamar esse aluno, precisa de conversar, precisa de entender, menos de julgar, e menos de

julgar, o que está acontecendo, ou seja, por que essa explosão aconteceu, é comigo mesmo? Deixa eu entender, é comigo mesmo, o que está acontecendo? Se for comigo o que a gente pode fazer para melhorar essa relação, então eu acho que a gente precisa ter uma escuta cuidadosa, terapêutica, refinada, para tentar entender o porquê dessa violência, então eu acho que passa por aí também.

Entrevistadora: Então, na sua fala você completou e respondeu, inclusive, a minha próxima pergunta que seria como são as possíveis estratégias de enfrentamento dessa violência que o aluno comete contra o professor. E teve algum momento, Douglas, que você sentiu, assim, Doutor Douglas, pessoa, você sentiu, assim, algum tipo de violência que te chamou a atenção que te chocasse, não com você, mas em contexto educacional?

Entrevistado: Eu acho que no contexto educacional eu sinceramente no dia a dia da minha pratica eu não lembro de nada, assim, tão marcante, mas, assim, eu lembro quando eu dava aula na UNIP teve uma aluna que chegou atrasado numa prova, era uma senhora já, e ela chegou atrasada e ela tinha um comportamento mais histriônico mesmo, assim, a gente percebia, tá, e aí a prova já tinha começado, tinha tempo já que a prova tinha começado, muitos alunos já tinham saído da sala, e a norma da UNIP, na época, era que, saiu, não entra mais alunos, e aí eu conversei com ela, já expliquei que ela deveria pedir segunda chamada, aí ela ficou extremamente agressiva, não comigo, de forma direta de [...], mas ela começou a reclamar, começou a xingar, que a vida estava muito ruim, que tudo era um inferno, e que ela ia cometer suicídio, e ela se jogou no parapeito, estava no corredor ela se jogou no parapeito como se ela fosse projetar o corpo fora do parapeito, a gente estava eu acho que no segundo andar, ela ia cair em cima dos carros, lá embaixo, e foi uma situação muito maluca, de extrema violência no sentido verbal e comportamental também, então, e a coordenadora que estava na outra sala que estava tendo prova, também, correu, tá, foi agredida, porque ela tentou pular, a coordenadora segurou, e ela foi agredida com chutes e uma agitação psicomotora que acabou levando a alguns ferimentos, que a gente percebeu depois, então foi uma situação muito complicada, muito difícil e muito traumática, muito traumática, e a aluna, eu fiquei um tempo lá, depois saí, tive outras oportunidades, mas são coisas que marcam, são coisas que você fica, por que isso está acontecendo, o que eu vou fazer para melhorar uma situação como essa, para fazer com que a pessoa consiga refletir sobre tudo isso, depois eu descobri que ela fazia uso de algumas medicações, estava fazendo tratamento com psiquiatra, mas, assim, mas tudo isso faz com que a gente fique muito preocupado, na época eu fiquei muito impactado com essa situação, com essa ação de extrema violência, então, mas eu acho que, assim, eu lembro dessa situação, especificamente foi essa.

Entrevistadora: Muito bem, obrigada. Professor, nós... Eu estou na minha última pergunta da entrevista e essa pergunta, dentro da metodologia qualitativa da abordagem eu deixei para que o entrevistado, o professor, pudesse complementar caso ele sinta necessidade de falar sobre essa temática, violência no ensino superior em saúde. Você gostaria de complementar, acha que vale a pena reforçar algum...?

Entrevistado: Eu acho essa temática muito interessante, eu acho que você e a professora Maria Alves estão sendo muito felizes em ter escolhido essa temática para discutir, eu acho que nós precisamos falar sobre a violência sim, está no contexto da

sala de aula, no contexto da prática clínica, do estágio, porque infelizmente a violência ela está muito impregnada no nosso dia a dia, e a gente precisa de tentar entender um pouco mais por que ela acontece, por que ela é tão frequente, e o porquê principalmente de nos adaptarmos a ela, de repente é algo tão dentro do contexto que fica... Torna-se algo mais natural, mais corriqueiro, e as pequenas violências não são percebidas como problema, você lembra das grandes violências, das impactantes, mas as pequenas violências do dia a dia de repente elas passam tão, de forma tão despercebidas que nós deixamos o que, de falar sobre elas, de discutir, né, e de tentar criar um ambiente mais saudável, tantos para os nossos alunos quanto para os professores e técnicos, então eu acredito que dá para ser... dá para ser mais feliz, discutir esses temas trabalhando com eles de uma forma que a gente possa se antecipar a uma situação mais agressiva, mais violenta, então eu acredito que a gente pode cuidar do outro, para que o contexto de violência, as situações de violência em si não estejam tão presentes no dia a dia, então eu acredito muito nisso. Eu acredito muito, também, que a violência é uma via de duas mãos, ou seja, a violência nunca acontece sozinha, ou seja, tem uma relação muito forte com ação e reação, então na universidade não é diferente, então acredito que as pequenas agressões verbais ou não verbais que acontece em sala de aula e nos estágios elas vão gerando o que, elas vão gerando um ambiente muito ruim para o aluno, então na FEN mesmo tem uma disciplina, tem até uma atividade no sexto período que é a simulação realística que a última simulação realística do ano, que teve ano passado ainda, os alunos do sexto período eles comentaram que para eles a faculdade estava começando no sexto período, porque antes do sexto período a vida deles eram muito difícil, era muito sofrida, sabe, todo tipo de violência aparecia, na fala, então principalmente no aspecto relacional, gente, nós precisamos mudar isso, né, ou seja, o ambiente ele tem que... Ele não só tem, mas ele precisa ser mais harmonioso, para o aluno que chegar no meio do curso com essa carga de sofrimento gigantesca, e eu acredito que nós temos muita culpa nisso, aí a gente precisa de melhorar, então acho que é mais nesse sentido.

Entrevistadora: Professor Douglas, olha, a sua contribuição é muito importante para a nossa pesquisa.

Entrevistado: Obrigado.

Entrevistadora: Nós nos comprometemos em fazer essa devolutiva assim que nós tivermos, já, para o ano que vem nessa conclusão da tese, a gente faz questão para os nossos participantes fazer essa devolutiva, que tem, assim, uma importância muito grande, quando o participante desloca o seu tempo, reserva um espaço para estar conosco, então eu reitero, aqui, mais uma vez o meu agradecimento e dizer que fico muito feliz, me desculpe pelas vezes a insistência, mas você sabe...

Entrevistado: Não, é assim mesmo, está tudo certo. Ângela, e, assim, eu vejo que você e Maria tem um grande desafio pela frente porque num momento de pandemia, como a gente está, as coisas ficam muito mais complicadas, eu participei de uma qualificação semana passada com a doutoranda de federal de Jataí e ela trabalha com idosos, e ela trabalha com a cartilha do idoso, e aí ela começou a aplicar a cartilha em janeiro, fevereiro, em março fechou tudo, e o trabalho dela é um trabalho que ela faz Quali, mas boa parte do trabalho é quantitativo, o ene dele ficou pequeno, por que, porque ela não

conseguiu aplicar mais, porque idoso é grupo vulnerável, e aí eu fiquei pensando, falei caramba, doutorado nesse momento, doutorado, mestrado, é um curso...

Entrevistadora: É um desafio viu, professor? [Risos].

Entrevistado: É um desafio gigantesco, eu acho que isso é um dado que você precisa colocar na sua tese.

Entrevistadora: Com certeza.

Entrevistado: Porque não é fácil, parabéns pela iniciativa, pelo tema, estou à disposição no que você precisar.

Entrevistadora: Está ok, muito obrigada, viu, professor? Muito obrigada mesmo e até logo, tchau.

Entrevistado: Até lá, um abraço.

Entrevistadora: Tchau.

Entrevistado: Tchau, tchau.

00:28:12

PARTICIPANTE 4
Tempo de gravação: 00:23:32

Entrevistadora: Então ok. Então, Bárbara, bom dia, né.

Entrevistada: Bom dia.

Entrevistadora: Eu sou Ângela, doutoranda da Faculdade de Enfermagem do Programa de Pós-Graduação de Enfermagem. De antemão eu já quero agradecer aqui a sua disponibilidade para esse momento da entrevista. Essa entrevista ela vai perdurar de vinte, de quinze a vinte minutos, e eu gostaria que você nesse momento falasse, permitisse o seu consentimento para que a gente possa prosseguir com a entrevista.

Entrevistada: Tá, Ângela, o meu nome é Bárbara Sousa Rocha, hoje é dia 15 de setembro de 2020, e eu permito a gravação sim da entrevista.

Entrevistadora: Ok, obrigada. Então, vamos começar com as questões fechadas Bárbara porque são muito importantes para nós nesse momento, né. A sua nacionalidade, por favor?

Entrevistada: Brasileira.

Entrevistadora: Estado civil?

Entrevistada: Solteira.

Entrevistadora: Qual a sua maior titularidade?

Entrevistada: Eu sou doutora.

Entrevistadora: Há quanto tempo você é professora Bárbara?

Entrevistada: Há doze anos.

Entrevistadora: Então, agora me diga assim, para você conceituar violência como que você conceituaria, né, o que você, o termo "violência" no conceito te remete?

Entrevistada: Ah, hoje eu penso violência é uma coisa bem mais abrangente, antigamente a gente tinha uma ideia de que violência ela precisaria vir acompanhada de uma agressão física, hoje eu entendo que violência ela é muito mais do que isso, é agressão física, é agressão psicológica, é agressão contra aquilo que a pessoa é, ou aquilo que ela, é, é... Ou qualquer limitador assim que afete a pessoa naquilo que ela gosta de ser, da maneira como ela é, ou aquilo que ela traz de raiz, de origem, eu estou falando muito assim nesse sentido do preconceito, né, do racismo, isso tudo entra em violência, eu não sei conceituar muito bem, né, mas eu sei que existem fatores hoje muito mais abrangentes do que apenas só o ato de ferir alguém fisicamente, então hoje a gente se sente ferido psicologicamente falando, mentalmente falando, ou até mesmo a questão do assédio moral, alguma pressão psicológica que a pessoa vive, porque assim, não é só dizer para a pessoa assim, ah, você é feio ou bonito, isso é uma violência, mas, por exemplo, numa reunião de trabalho eu me sinto às vezes muito afetada pelos assédios, do tipo, ah, dando a impressão de você não está fazendo ou que você não está cumprindo o seu papel, isso para mim é uma violência, eu me sinto afetada com isso.

Entrevistadora: Ok. Para você quais fatores desencadeiam a violência?

Entrevistada: Eu acho que, assim, eu vou começar do começo, na minha opinião a primeira é um desiquilíbrio da pessoa que promove a violência contra a outra, é o primeiro, eu acho que essa pessoa está afetada mentalmente, psicologicamente falando, quimicamente falando, ou tem alguma patologia que leva essa pessoa a desencadear uma violência contra a outra. Forante isso, eu também acho que a intolerância, a impaciência, o estresse ou até mesmo as vaidades e as ambições levam a uma pessoa a violentar a outra.

Entrevistadora: Ok. Você já sofreu algum tipo de violência durante a sua prática pedagógica?

Entrevistada: Em sala de aula alguma vezes, mas, assim, não que tivesse me afetado tanto assim, é uma violência psicológica às vezes, mas eu não tenho um fato para te contar assim, eu não me recordo, mas no trabalho tem demais, nas relações de trabalho.

Entrevistadora: Ok. Não assim, porque talvez fosse física, moral, verbal, que algum aluno desencadeasse na sua prática, se você já havia sofrido?

Entrevistada: Não assim, não que eu me recorde.

Entrevistadora: Mas no trabalho você refere que sim, né, nas relações de trabalho?

Entrevistada: Nas relações de trabalho que para mim eu acho que assim, a prática pedagógica é tudo, né, então, desde...

Entrevistadora: É tudo.

Entrevistada: Do momento que eu estou planejamento uma disciplina, que eu estou participando de um projeto de pesquisa, que envolve também a participação dos alunos e outras pessoas, eu acho que ali é uma prática pedagógica, e nesse sentido eu já sofri, muito assédio, né.

Entrevistadora: Isso. E diante dessa situação qual é a sua atitude Bárbara?

Entrevistada: Primeiro de tudo a perplexidade, né, que eu fico pensando assim, às vezes, por exemplo, eu falo do... Vamos começar do aluno, né, o aluno quando às vezes eles se remetem a gente de uma forma que parece que ele não considera que você é o professor, não que eu ache que a gente tem que estar numa posição superior, né, que isso não existe mais, a gente é facilitador do processo e tudo mais, mas às vezes não diz respeito assim do tipo, aquele momento que o aluno não está nem aí para o que está acontecendo, aquilo é um desrespeito. Eu me perdi Ângela, o que você tinha perguntado? Desculpa.

Entrevistadora: Eu perguntei qual a sua atitude, né?

Entrevistada: Ah, a minha atitude, então, assim, primeiro eu fico perplexa, né, aí primeiro eu fico perplexa, eu falo meu Deus, porque quando eu era aluna os meus professores eu tratava assim como... Aquela coisa, né, aquela redoma, não é, não vejo isso mais, então eu fico perplexa. Em reuniões de trabalho também a primeira atitude é ficar perplexa, não ir para o embate, eu fico muito reflexiva, eu preciso pensar sobre a violência que eu sofri, digerir para depois fazer alguma coisa...

Entrevistadora: Entendi.

Entrevistada: Então, às vezes eu retomo para a pessoa um dia depois, ou eu ligo para a pessoa, eu tento retratar aquele movimento, assim, para saber qual foi a intencionalidade da pessoa em fazer aquilo, né, falo assim, o que está acontecendo, né, principalmente se for colega de trabalho eu falo, tem alguma coisa errada entre a gente, então eu tento tirar, ver se aquela situação se resolve mesmo sendo contra mim.

Entrevistadora: Entendi.

Entrevistada: Mas nunca fui para vias de fato, vamos supor, nunca fui para vias de fato assim, tipo, mover um processo contra alguém, sabe, não, eu nunca...

Entrevistadora: Compreendi. E durante a formação dos profissionais de saúde você nessa frente como professora, essa temática violência você contempla nos seus conteúdos, você discuti, contempla, ver, nos currículos mínimos sempre está pontuado quando se reúnem aí para o PPC, para planejar, a temática violência ela fica inserida nas suas disciplinas, está inserida?

Entrevistada: Não muito, é muito sui generis, assim, é muito ampassã, a gente fala de violência, por exemplo, na disciplina de saúde coletiva, a gente aprofunda mais na questão assim das classes sociais, da injustiça social, das desigualdades, do racismo. E assim, na disciplina de promoção da saúde a gente trabalha a cultura da paz na escola, então a gente... Essa tem uma temática sobre violência, então quando a gente chega na escola e a escola propõe essa temática para a gente trabalhar, e a gente trabalha, porque é um eixo da política nacional de promoção da saúde, combate à

violência e favorecimento da cultura da paz. Agora não que ela seja um conteúdo a disciplina, entendeu? Eu não tenho assim tipo, três horas para falar de violência, eu não tenho em nenhuma das duas disciplinas. E no estágio supervisionado eu trabalho a violência conforme ela vai aparecendo, então às vezes o aluno ele se ver numa situação de violência ou de assédio dentro das relações, ele vem e conversa com a gente, aí eu tenho trabalhar, mas também não é uma temática especifica.

Entrevistadora: Fazendo o link para você, se você conceituaria a violência, como que você conceituaria. Agora como eu você considera violência contra o professor?

Entrevistada: Eu acho que... É tão difícil, né [Risos], a gente sente na pele, mas na hora de dizer o que é, né, porque às vezes a gente é violentado diariamente e não sabe que aquilo é uma violência, então é difícil conceituar, eu acho que sei lá, vai muito para a linha do desrespeito, da desconsideração, assim, sabe, parece que ... Aí muito difícil, aí [Risos], às vezes você sente, mas não sabe dizer o que é, então, eu não quero ser aquela pessoa que fala, ah, porque eu sou professora o aluno tem que chamar, por exemplo, de professora senhora, né, mas acaba que o aluno que não faz isso ou aluno que não te coloca numa redomazinha aquilo você se entristece, mas talvez aquilo não seja violência, né, então eu acho que a gente tem muito que desmistificar também do papel do professor na relação, relação aluno/professor, sair de um pedestal, eu falo por mim, né, e entender o que de fato seria violência nesse processo, por que a gente... Eu sei que tem colegas que vivenciam muitas situações de violência, mas na universidade eu estou sendo muito mais violentada pelas políticas públicas, né?

Entrevistadora: [Risos].

Entrevistada: Pela falta de recursos do que necessariamente pela nossa relação professor/aluno. Eu estou me lembrando aqui de uma situação que eu tive com uma aluna, mas que a gente também se resolveu, sabe, eu acho que era uma situação muito mais de incompatibilidade de gênios assim, assim, de ela ser uma menina de gênio muito forte e eu também, mas foi muito mais nessa situação. E difícil essa pergunta.

Entrevistadora: [Risos] É sim. Eu perguntei sobre, né, a sala de aula e o que você considera violência contra o aluno, contra o professor, violência do aluno contra o professor. E se você desenvolvia alguma prática, nesse momento você desenvolve alguma prática enquanto professora para academia, para toda a academia, porque você já citou, né, a condição institucional, as relações de trabalho, em algum momento você desenvolve na sua prática ações voltadas?

Entrevistada: Você fala assim, para dirimir essa situação de violência?

Entrevistadora: Exatamente.

Entrevistada: Então, eu sou uma professora muito ligada as relações, né, então eu tento favorecer muito a convivência em grupo, para que essa relação nossa fique cada vez mais saudável, então eu sou uma professora assim, por exemplo, que no remoto, né, mesmo nas atividades remotas, eu começo a aula com música, ali você já quebra um pouco, qualquer intolerância, impaciência você já coloca a pessoa para refletir, eu sou aquela professora que gosto do acolhimento, de apresentar, eu acho que tudo isso é criar um ambiente saudável que desfavorece a violência de alguma forma, tanto para

mim quanto para eles. Eu também tento me policiar na utilização de termos que possam ser preconceituosos, que possam ser também termos que instigam a violência, né, eu tento muito fazer isso, então eu acho que talvez essa fosse a minha principal tarefa. Na FEN mesmo, por exemplo, com os colegas eu sou a professora da agregação sabe, eu sou a professora da integração, ah, vai fazer uma festinha é a Bárbara, porque eu gosto muito disso, inclusive eu estou sofrendo muito com essa relação virtual porque eu sou do abraço, eu sou do chegar, do conversar, então eu acho que talvez o meu próprio jeito de ser ele ajuda, ele favorece esse processo de não violência, eu acho que é isso.

Entrevistadora: Ok. E assim Bárbara, especificamente faz com que ele cometa violência?

Entrevistada: Deu uma cortadinha Ângela, você repete, por favor.

Entrevistadora: Repito. Qual fator ou assim qual atitude, qual ação que leva o seu aluno a cometer violência, você acha que existe um fator específico ou alguns fatores que um aluno, que favoreça um aluno a cometer violência?

Entrevistada: Eu acho que sim, os fatores externos são os maiores fomentadores da violência, principalmente as condições sociais, as desigualdades, a injustiça, eu acho que o quanto esse aluno às vezes ele tem que se esforçar para chegar onde ele tá naquele momento, aí de repente ele chega e sei lá se decepciona com algum conteúdo ou alguma forma que está sendo abordada, ele fica intolerante, ele fica impaciente, ele cobra mais, né, ele se posiciona mais, até no sentido assim de que ele sempre foi um lutador da sua vida, porque nada para ele foi fácil, então isso facilita com que ele seja essa pessoa aguerrida demais que eu estou sempre em alerta, em situação de guerra, estão sempre contra a mim, eu vejo muito isso na UFG porque a maioria dos nossos alunos estão nessa condição, eles são alunos que precisam lutar muito para estar onde estão, e por isso eles ficam muito na defensiva, então eu acredito que fatores sociais, econômicos, políticos, eles influenciam na postura de violência das pessoas sim, principalmente dos nossos alunos.

Entrevistadora: Certinho. Então, assim, agora você já vivenciou algum momento de violência que te chocasse. Você pode falar com você se foi ou que você vivenciou, com outro colega, na academia, entre alunos, que te chamasse atenção professora Bárbara?

Entrevistada: Então, igual eu te falei, com aluno especificamente eu não tenho nenhuma experiência que eu consiga me lembrar, eu acho que as coisas foram se resolvendo, são coisas muito talvez comuns assim, né, mesmo sendo violência. Mas com colegas de trabalho a gente ver muito assédio, por exemplo, recentemente eu faço parte de um grande projeto que eu sou a vice-coordenadora, e o coordenador ele é um assediador, ele é uma pessoa que ele faz as coisas, ele é muito competente, mas ele coloca as pessoas, ele nivela as pessoas a ele e não valoriza nada do que as pessoas fazem, as pessoas que estão no projeto, e isso é uma violência. Então, ontem mesmo, por exemplo, eu estava numa reunião e claramente foi uma reunião violenta, sabe, foi uma reunião de cobrança velada, não é aquela cobrança assim, olha, gente temos isso, vamos fazer isso, vamos, não é assim, eu espero que esteja pronto, eu espero que tenha sido feito, sabe. Então, assim, esse ano mesmo com toda essa parte assim da pandemia eu me senti muito violentada em vários projetos, né, na FEN mesmo a gente tem um grande projeto, e aí também a gestão desse projeto ela é uma gestão

assediadora, eu me senti violentada em muitos momentos, então é mais nesse sentido, sabe, de aluno mesmo diretamente não consigo me lembrar, mas eu não sou muito boa de memória também não, sabe, esse é o meu defeito.

Entrevistadora: [Risos] Ok. E Bárbara como que você vê ou se é possível alguma estratégia na sua visão, assim, colocando esse óculos aí, enxergando aí. Quais estratégias de enfrentamento a violência você ver assim para, não reducionismo, mas assim, minimizar essa violência dos alunos contra o professor?

Entrevistada: Aí não sei, eu acho que deveria haver mudanças profundas na sociedade, essa é uma, porque eu também não quero culpabiliza a vítima, eu fico sempre com esse pensamento, porque às vezes o violentador, o aluno ele também é uma vítima social, então eu acho que tinha que haver políticas públicas profundas de mudança cultural, da sociedade, dessa desigualdade toda que existe, e internamente eu vejo políticas corretivas como Bolsa Inclusão, a Casa do Estudante, por exemplo, agora a gente tá com essa política de acesso as ferramentas de informática para o retorno das aulas, eu vejo que a UFG é muito preocupada com isso, né. E eu acho que também deveria haver internamente, por exemplo, na FEN que é o nosso caso, deveria haver não sei, uma sensibilização dos professores para que eles consigam ter mais tolerância com as situações postas, então eu vejo que tem professor que tipo, não tem essa noção de que o aluno veio no ônibus, que ele não tinha o passe de ônibus, aí ele chega lá e o professor fala, olha, não vai ter aula, aí o aluno fica puto, fica com raiva, e o professor com raiva também ali já acontece um processo violento de ambas as partes. Então, eu acho que talvez uma sensibilização da consciência do todo, de como lidar com essas situações, de como também a gente pode ser mais sei lá respeitoso com a situação do aluno e vice e versa, para a gente ter o respeito, eu acredito que as escolas de ensino fundamental e médio eles sofrem muito mais a violência direta e física, o professor, na universidade eu sei que existe, mas eu acho que precisa de uma análise mais profunda assim do porquê ela está existindo, na minha ideia, né.

Entrevistadora: Entendi. Eu vou para minha última pergunta, e nesse momento eu deixo assim livre para que você caso queira complementar algum momento que ao ficou claro, retomar sobre essa questão de violência contra o professor. Então, assim, se você quiser complementar ou pontuar, ou algo que ficou, que você gostaria de complementar fique à vontade?

Entrevistada: Eu só queria dizer se eu não me lembro do que aconteceu comigo e porquê de fato não me feriu tanto, mas isso não significa que não seja violência, né, eu sei que eu já tive momentos de me sentir chateada com uma ou com outra situação dentro de sala de aula, mas que provavelmente eu tenha resolvido depois com esse aluno, né, eu desconheço um aluno que tenha se formado ou saído sem a gente conversar, a gente... Tem situações de que o aluno às vezes nós constrange pela forma como ele exige o direito dele, isso também é uma violência, às vezes por e-mail, por mensagem, né. Recentemente agora eu estou lembrando de uma situação, não foi diretamente comigo, mas o aluno gravou um vídeo e postou nas redes sociais falando do comportamento dos professores em geral sobre o estágio, e foi até num tom ameaçador, né, então a gente teve que fazer todo um cuidado com esse aluno, e com aquilo que ele estava reverberando, mas coisas que a gente consegue resolver apesar de nos afetar. Quero dizer que as suas perguntas foram muito difíceis, principalmente

porque são questões que a gente não para pensar, a gente não para conceituar, a gente muitas vezes vai vivendo essa situação e não entende que ela nos afeta no final do mês, no final do ano, nas situações da docência. E te dar parabéns por essa pesquisa, e eu quero saber o resultado dela [Risos].

Entrevistadora: Bárbara olha, [...] por ter aceitado, muito importante as suas contribuições, assim fiquei muito feliz com o encadeamento que foi desenvolvendo a entrevista, porque o pesquisador qualitativo tem isso mesmo, ele fica tocado aqui, né [Risos] com essas respostas.

Entrevistada: A gente mal consegue se conter de falar, e aí gente.

Entrevistadora: Isso, exatamente. Então, muito obrigada mesmo, viu, e com certeza nós vamos estar devolvendo sim esse resultado, né, e quem sabe, né, até mesmo algum produto interessante para ser desenvolvido aí no futuro próximo, tá bem. Olha, um grande beijo, muito obrigada viu, Bárbara, tchau.

Entrevistada: Boa sorte e bom trabalho, tá.

Entrevistadora: Obrigada, tchauzinho.

Entrevistada: Tchau, tchau querida.

00:23:32

PARTICIPANTE 5

Tempo de gravação: 00:13:36

Rafael: E autorizo a gravação.

Entrevistadora: E a data de hoje, professor, por favor.

Rafael: Dezessete/09/2020. Autorizo na data de 17/09/2020.

Entrevistadora: Excelente, obrigada então. Professor, eu sou doutoranda aqui da FEN, da faculdade de enfermagem, e a minha temática, o meu objeto de estudos é a violência contra o docente na prática em saúde, uma abordagem intercultural, e aí, professor, essa entrevista ela vai perdurar em torno de quinze a vinte minutos e eu... ela tem primeiro as questões fechadas, uma abordagem qualitativa, eu vou fazer as perguntas todas essas questões fechadas que tem nesse questionário, depois a semiestruturada, as questões abertas. Então, professor, qual a sua idade em anos?

Rafael: Tenho vinte e oito anos.

Entrevistadora: Sexo?

Rafael: Masculino.

Entrevistadora: Nacionalidade.

Rafael: Brasileira.

Entrevistadora: Qual o seu estado civil?

Rafael: Solteiro.

Entrevistadora: E qual seu grau [...].

Rafael: Cortou Ângela.

Entrevistadora: O seu grau de escolaridade, a maior titularidade.

Rafael: Eu tenho doutorado.

Entrevistadora: Professor Rafael, há quanto tempo que você é professor?

Rafael: Um ano.

Entrevistadora: Um ano. Então agora eu começo com as questões abertas. Doutor, qual... como você conceituaria a violência?

Rafael: Violência é qualquer agressão externa, a grosso modo, que causa algum dano à pessoa, aí pode ser externo, interpessoal, ou própria, acho que esse é o termo.

Entrevistadora: E para você quais são os fatores que podem desencadear a violência?

Rafael: Fatores? Primeiro eu, assim, eu estou pensando do ponto de vista de estudos, mas alguns fatores podem ser sociodemográficos como renda baixa, sexo, orientação sexual, grau de escolaridade são fatores sociodemográficos que influenciam na vivenciam e aí tem alguns outros fatores, que eu acho, por exemplo, o consumo de substâncias em geral tanto licitas quanto ilícitas, e também alguns cenários ambientais, ambientes em que tem violência com os pares, na mesma família, acho que seria isso, os principais.

Entrevistadora: Você já sofreu, professor Rafael, algum tipo de violência durante a sua pratica pedagógica quando você está ministrando as suas aulas?

Rafael: Independente de... eu sofri durante o projeto de extensão.

Entrevistadora: Então sim. E assim, você... qual foi a sua atitude diante desta violência?

Rafael: Nada, só fiz boletim de ocorrência. [Risos] nada, só boletim, eu fiz boletim porque a gente estava atendendo a população, foi bem agora na pandemia, sabe, tem uns dois meses, e aí a gente oferece alguns testes de Covid para a população no projeto de extensão, e aí tem os critérios, e algumas pessoas não aceitaram, tem uma mulher que não aceitou e agrediu eu e outra professora, sabe?

Entrevistadora: Foi uma agressão física.

Rafael: Foi, e aí por isso que eu falo de transtorno mental, essa mulher, depois a gente descobriu, ela, nesse projeto a gente atende profissionais, então foi uma médica que agrediu a gente, então ela sofre de transtorno bipolar, sabe, a gente até conhecia ela e tudo, mas foi durante o projeto de extensão.

Entrevistadora: Entendi. E aí foi uma agressão física ou só verbal, qual o tipo?

Rafael: Foi inicialmente verbal e depois foi... a minha colega foi física, eu não, minha colega foi mais persuasiva, e fez boletim de ocorrência por justiça.

Entrevistadora: No seu entendimento durante a formação dos profissionais da área de saúde essa temática violência contra professor ela está contemplada nos conteúdos mínimos e nos projetos pedagógicos onde você é professor?

Rafael: Não, nem no curso, assim, que você aprende no mestrado algumas... que é docente mesmo, nem nos cursos de formação para entrar na universidade, então o nosso curso, por exemplo, não tem esse conteúdo, ele tem assédio, mas assédio via professor-aluno, assédio de violência não, a gente nem sabe o que fazer nesse caso.

Entrevistadora: Considera, agora sim, fazendo um recorte, o que é violência contra o professor, o que você considera uma violência algumas ações de violência contra o professor?

Rafael: Repete, porque... Ângela.

Entrevistadora: Oi. Cortou?

Rafael: Muito chiado, espera aí. Tá, passou, pode repetir.

Entrevistadora: Ok, então, durante... a pergunta é assim, na sua opinião o que você considera uma violência contra professor? Por exemplo, o que você considera que o aluno comete uma violência contra o professor?

Rafael: Além de violência física, violência verbal, por exemplo, as formas de cometer, tem, assim, pelo menos o que alguns colegas já passaram, é um conceito novo, mas eu acho que aquela violência digital, algumas, alguns e-mails bem, assim, desaforados.

Entrevistadora: Com certeza, professor.

Rafael: Nem, assim, nem e-mail, WhatsApp, sabe, então, assim, eu considero essa pior, que às vezes aluno não fica contente e ele fala cada coisa para a gente, então, assim, o que eu mais vejo é essa violência verbal mesmo, na universidade.

Entrevistadora: E você desenvolve durante a sua experiência como professor, durante as suas aulas, prática docente, você desenvolve respeito da comunidade acadêmica, não só, assim, por exemplo, na sala de aula, mas em toda comunidade acadêmica, você desenvolve alguma ação?

Rafael: Não, ainda não realizei nenhuma ação, assim, nem informativa, nem alguma mobilização, alguma roda de conversa sobre esse tema, até porque eu não tenho formação, não tenho capacitação, eu não sei muita coisa.

Entrevistadora: E no seu entendimento o que leva um aluno e usar de violência contra o professor durante as aulas?

Rafael: Eu acho, assim, primeira coisa essa pauta, também, de ações de educação para esse aluno, então talvez um curso quando ele inicia sobre respeito, sobre questão

de violência acadêmica, tanto ascendente quanto descendente, falta de capacitação dos próprios professores, das disciplinas, então poderia ser um tema transversal em todos os períodos, eu acho que isso.

Entrevistadora: Bacana isso, tema transversal seria uma boa sugestão mesmo. E até [...] você vivenciou alguma violência de aluno contra professor, não precisa ser diretamente com você, mas que você presenciou e que te chocou, te deixou, assim, impactado?

Rafael: Eu já vivenciei violência digital, assim, existia um grupo da disciplina, um aluno não gostou de uma atividade avaliativa e ele mandou barbaridades com a professora xis lá, colega da disciplina, assim, foi muito constrangedor, foi... teve xingamento dela, para ela, então, assim, foi um absurdo, na universidade.

Entrevistadora: E você vê, assim, se fosse para você levantar algumas estratégias para esse enfrentamento de violência para desenvolver com os alunos você sugeriria algumas estratégias que poderiam minimizar esse impacto da violência do aluno contra o professor?

Rafael: Talvez algumas estratégias como, por exemplo, inserir no curso de formação inicial do professor esse conteúdo, né, de violência contra os professores e manejo, porque não é só aspectos teóricos, conceitos de como agir, né?

Entrevistadora: Exatamente.

Rafael: Outra estratégia poderia ser... e esse professor poderia replicar esse conteúdo também em forma de educação e saúde nas disciplinas, isso seria outra estratégia e uma terceira estratégia também é ter um núcleo de pesquisa em violência, que aí você poderia inserir também esse conteúdo, violência contra os professores, né?

Entrevistadora: Com certeza, é isso mesmo. Nós estamos na última pergunta, não é nem uma pergunta, nesse espaço eu sempre sugiro aos participantes de eles querem contemplar alguma fala sobre violência que não foi aqui nas minhas perguntas contempladas, se ele quer complementar alguma pergunta. Você gostaria de complementar, falar sobre essa temática, especificar algo mais?

Rafael: Não, só que, assim, o que eu quero acrescentar é que eu chão de triangulação, triangulação de formas de agir contra a violência, deve ser feita entre docentes, alunos e gestores das universidades, acho que eu resumiria isso.

Entrevistadora: Ok, ok, muito bom, muito bom mesmo, professor Rafael, olha, nós estamos um pouco mais, já, uns 80% durante essa minha coleta de dados já para conclusão e assim que nós tivermos um resultado tenha certeza que eu estarei reportando aos participantes e quero muito agradecer porque suas contribuições serão valiosas aqui para meu estudo, então muito [...] de verdade e reitero aqui, me desculpe a insistência, mas é porque você sabe, vida de doutoranda como é que é, a gente tem que ficar realmente organizando o tempo e com o processo da pandemia foi muito difícil porque eu tive que mudar o formato passando para as atividades remotas, então isso dificultou um pouco, eu quero te agradecer imensamente, professor, muito obrigada.

Rafael: Não tem nada não, é o tempo, o problema foi o tempo, mas agora deu certo e boa sorte na sua análise aí de dados.

Entrevistadora: Ok, muito obrigada, viu, tenha um bom dia, um restinho de dia, até mais professor, tchau.

00:13:36

PARTICIPANTE 6

Tempo de gravação: 00:11:19

Entrevistadora: Pode gravar, né. E quais são as iniciais do seu nome, professora?

Regiane: RASSB.

Entrevistadora: Ok. Professora, qual seu estado civil?

Regiane: Casada.

Entrevistadora: E qual a sua titularidade, a sua maior titularidade?

Regiane: Doutora.

Entrevistadora: Há quanto tempo você é professora?

Regiane: Vinte e três anos.

Entrevistadora: Então, essas são as perguntas do perfil [...] agora uma conversa para a gente trabalhar a temática violência. Para você qual é o conceito de violência, professora Regiane?

Regiane: Eu acho que... violência que você fala é violência na área docente, né? Ou geral?

Entrevistadora: Geral, por enquanto geral.

Regiane: Ah tá. É quando a pessoa é submetida a um... nossa, difícil, né, ou a uma ameaça ou a uma... difícil, você me legou numa hora difícil.

Entrevistadora: [Risos] Tudo bem, tudo bem.

Regiane: Vamos pensar então. Violência, como o próprio nome diz, é uma agressão a dignidade do ser humano por motivos qualquer que pode ser desde o motivo, desde sem motivo ou até motivado por alguns interesses, mas a questão maior é ela pode ser moral, ela pode ser física, ela pode ser emocional, psicológica, acho que é isso.

Entrevistadora: Então, partindo desse conceito amplo de violência, você acredita ou poderia pontuar ou afirmar se existem fatores que são desencadeantes da violência?

Regiane: Fatores desencadeantes da violência, olha, eu acho que na sociedade atual sim, existem fatores desencadeantes, o estresse é um fator desencadeante, alguns tipos de agressões verbais, sim, podem desencadear a violência.

Entrevistadora: Agora vamos trazer aqui para a pratica pedagógica, você já sofreu algum tipo de violência durante a sua prática pedagógica, professora Regiane?

Regiane: Olha, que eu me senti ameaçada alguns anos atrás por um aluno que não aceitava sua nota, já sofri violência, e por professores quando eu era coordenadora de curso.

Entrevistadora: Então a academia também, na instituição também é uma finte de violência.

Regiane: Com certeza.

Entrevistadora: E diante dessa violência que você especifica ter sofrido quais foram as suas atitudes relacionadas a essa situação?

Regiane: Pois é, eu tomei, assim, atitudes, no caso dos professores eu tomei atitudes administrativas, até tivemos um processo administrativo por conta disso, agora, com relação ao aluno eu me senti tão intimidada que eu não fiz nada, eu só fiquei realmente com medo.

Entrevistadora: Em seu entendimento, assim, durante a formação profissional na área da saúde essa temática violência contra professor você visualiza que ela está contemplada nos conteúdos mínimos dos projetos pedagógicos?

Regiane: Eu creio que não, não é contemplada na sua totalidade e, assim, eu acho que nem na teoria e nem na prática, pode ser que o tema seja contemplado em um currículo oculto por alguns professores quando se deparam com algumas situações na prática do dia a dia, mas isso não é contemplado estrategicamente.

Entrevistadora: Bom, agora sim, dentro dessa prática, dentro da sua prática o que você considera violência contra professor, nós começamos com conceito de violência, agora especificamente contra o professor, o que você acha que é violência contra o professor?

Regiane: Olha, às vezes acontece assédio moral de aluno para professor com agressividade verbal mesmo, violência contra o professor, física, eu nunca sofri, mas eu acho que isso pode acontecer, dependendo do caso, já vi alguns casos, já ouvi falar e já vi, agora, assédio moral é o que mais acontece.

Entrevistadora: Durante esse assédio, esse tipo de violência você desenvolve experiências na sua prática docente que vise o respeito, assim, a toda a comunidade acadêmica, não só a docentes ou discentes, mas práticas que você desenvolve.

Regiane: Infelizmente não.

Entrevistadora: Você acha que o aluno usa de violência contra o professor durante a sua prática pedagógica por quê? O aluno ele tem algo que ele desenvolve, existe uma contrapartida na sala, alguma coisa assim para ele desenvolver?

Regiane: Eu acho que o aluno hoje em dia a relação é muito mais próxima, então existem alguns mecanismos que o protegem, não eu antigamente era o contrário, mas o respeito ao professor antigamente era mais levado a sério, e hoje em dia não existe mais esse respeito, a relação é muito próxima e eles confundem a proximidade com falta de respeito.

Entrevistadora: Na nossa conversa aqui anterior você falou assim, olha, eu considero uma violência, essa violência velada, essa violência emocional que pode acontecer, até hoje, durante a sua prática o que mais te impressionou sobre violência contra professor, não necessariamente que tenha sido você, mas que você já ouviu, nossa, isso...

Regiane: Contra o professor?

Entrevistadora: Isso.

Regiane: Olha, eu, assim, ainda bem que eu sofri... eu tenho consciência de que eu tenho vivido pouco isso, acho que isso é até bom, mais, assim, a ponto de o aluno não respeitar o professor mesmo, não respeitar a nota, não respeitar a avaliação que ele fez e partir para uma agressividade verbal para com o professor, isso foi o máximo que eu sofri e que eu tenha visto, agora, as únicas coisas que eu posso dizer é violência do professor para com o aluno, que essa eu vi mais.

Entrevistadora: Entendo. E você vê possíveis estratégias de enfrentamento a violência dos alunos contra o professor?

Regiane: Essa é mais difícil no mundo atual, porque a gente tem uma passividade com relação aos alunos, a gente não consegue, o sistema não permite que a gente faça, execute algumas ações para com eles... só um minutinho [...] o sistema muitas vezes não permite que a gente faça, que a gente tenha determinadas atitudes e eu acredito que são poucas ou quase nenhuma condição de a gente fazer isso, infelizmente.

Entrevistadora: Nós estamos já finalizando, é minha última pergunta e eu sempre faço, deixo dentro da abordagem qualitativa um espaço para que você possa falar se ficou alguma coisa que você gostaria de falar sobre essa temática de violência [...].

Regiane: É interessante o tema que você está estudando, eu acho que você vai colher muitos frutos sobre isso, mas eu acho que você vai ter uma certa dificuldade na hora das estratégias mesmo, eu acho que a gente precisa trabalhar bem essas estratégias e acredito que um grupo focal talvez seria interessante para trabalhar essas estratégias porque um vai dando margem a discussão e o outro elemento vai abordando outros aspectos e seria interessante, assim, você dar um ponto, pelo menos uma meta para a gente atingir nessa sua entrevista, porque você me pegou de surpresa, eu sabia do tema, mas eu realmente fiquei... fui pega de surpresa para desenvolver algum conceito em cima do tem.

Entrevistadora: Entendi, certinho, professora, está anotado, pontuado aqui, obrigado. Professora, eu vou fazer, eu acredito que esse trabalho assim que eu tiver os produtos eu estarei divulgando, de antemão agraço você ter achado um horário na sua agenda para estar me atendendo, muito obrigada de verdade, professora.

Regiane: Imagina, de nada.

Entrevistadora: Está ok, obrigada.

00:11:19

PARTICIPANTE 7

Tempo de gravação: 00:42:38

Entrevistadora: Fazer esse momento. Internet ultimamente está... Bom, professora você pode agora já iniciar o seu consentimento por favor.

Entrevistada: O meu nome é PTS e eu autorizo a gravação de voz e imagem.

Entrevistadora: Ótimo, muito obrigada. Professora, me diga qual é [...]?

Entrevistada: Desculpa Angela, cortou, você pode repetir a pergunta.

Entrevistadora: Posso. O seu estado civil professora.

Entrevistada: Solteira.

Entrevistadora: E qual que é a sua titularidade, o maior grau de titularidade.

Entrevistada: Eu sou Doutora.

Entrevistadora: Doutora. Há quanto tempo você é professora Patricia?

Entrevistada: Desde de 2012, então nove anos.

Entrevistadora: Nove anos. Então agora depois dessas perguntas vamos levando assim nessa conversa, porque o qualitativo é sempre assim mesmo a abordagem, né. Então para você professora Patricia, qual é o conceito de violência?

Entrevistada: Acho que é difícil, né, porque a gente vai buscar o conceito teórico, eu vou tentar dizer o que está na minha cabeça sem me preocupar muito com as teorias, mas para mim violência é um conceito bastante amplo, né, é multifatorial, e eu acho que em especial ele tem níveis, não sei se níveis, mas eu acho que pensando muito nas questões de violência física, mas também existem outros tipos, né, de violência, então tem as questões também de assédio que de alguma forma, né, podem ser violentas, amplio ainda mais pensando no ensino, né, estou pensando bastante no ensino viu Angela, ou a pergunta pode ser mais aberta?

Entrevistadora: Nesse primeiro momento ela pode ser mais aberta, nós vamos afunilando...

Entrevistada: Me vem muito quando penso em violência que a questão de violência contra a mulher e contra criança, né, que pode ser uma violência sexual, uma violência moral, patrimonial, para além da psicológica, para além da violência física, mas também me vem um pouco já pensando no trabalho assim, passei por situações de trabalho que o ambiente de trabalho e as condições de trabalho de alguma forma me afetavam, né, era violento aquele volume de trabalho, então ampliando muito, ampliando o conceito

mesmo, né, não vi isso em lugar nenhum, mas eu sentia como uma violência, né, eu acho que é isso.

Entrevistadora: Então, professora, então, assim, e pegando aí esse entendimento seu de violência, você acredita que tem fatores que são desencadeantes da violência?

Entrevistada: Eu acho que sim, acho que sim, eu estou pegando no tranco Angela [Risos].

Entrevistadora: Não tudo bem, não está excelente, é isso mesmo.

Entrevistada: Eu acho que sim, pensando, por exemplo, numa situação ampla de trabalho, né, eu já sofri violência física como enfermeira, né, e o motivador da violência foi o tempo estendido de atendimento, então estava demorando muito o atendimento, e aí juntando isso como a questão era uma questão psiquiátrica mesmo da pessoa que me agrediu, ela não quis agredir o médico, resolveu me agredir, também tem essas relações de poder, eu sou enfermeira, então... e sou mulher, né, então eu acho que tem algumas coisas que desencadeiam o momento de violência em si, então, que podem ser questões do ambiente, que pode ser as frustrações que o outro tem, longe de culpabiliza quem sofre a violência, mas às vezes uma palavra que a gente encaixa errado, uma atitude. De novo, uma outra vez que eu sofri violência também aí já numa instituição de saúde mental, eu dei as costas para a pessoa, porque eu não percebi que ela estava falando comigo, e aí eu levei uma bolsada na cara [Risos], hoje eu dou risadas, mas eu chorei muito, mas por conta disso um despreparo, né, para atuação naquele setor, depois conversando com colegas, Patricia ela estava falando com você, você que não percebeu que ela estava falando com você, e você ignorou a pessoa numa demanda importante, então eu acho que o despreparo, de novo, longe de culpabilizar quem sofreu a violência, mas tem algumas coisas que se você está mais bem preparado você se protege, então eu acho que isso é um fator, os fatores do ambiente, o contexto, então nessa situação de pandemia a gente está flor da pele, está todo mundo à flor da pele, né, está todo mundo frustrado porque teve que rever os seus planos, então conversando com só estudantes ontem eles não vão se formar no prazo que eles esperavam, e estão sem perspectiva de quando vai acontecer a formatura, né, então eu como estudante eu contava os dias para a minha formatura porque eu precisava trabalhar, então eu imagino que muitos deles também estejam nessa condição. Então eu acho que tentando resumir, eu acho que tem fatores que são próprio das pessoas, né, do seu sentimento, das suas condições e etc, eu acho que tem fatores que são do contexto, então se você está num ambiente violento você meio que incorpora aquilo de alguma forma, você fica reativo, né, tem o contexto social como um todo, né, e tem o preparo mesmo das pessoas que estão em alguma posição de poder, e aí eu considero que ser professora é uma posição de poder, eu considero que ser enfermeira é uma posição de poder em relação ao paciente, em relação ao estudante, então quem está nessa posição de poder também precisa preparar para não, bem entre aspas, estimular situações de violência, então, eu acho que eu fosse resumir isso tudo que eu quis dizer eu acho que seriam esses os pontos, né, que a gente poderia olhar.

Entrevistadora: Eu acredito professora que você já responde assim a minha próxima pergunta, mas agora voltado bem para a prática pedagógica, você já sofreu algum tipo de violência durante a sua prática pedagógica?

Entrevistada: Durante a minha prática pedagógica não, como professora...

Entrevistadora: Nem física e nem moral, verbal?

Entrevistada: Eu acho que houveram tentativas, né, na minha prática, eu acho que eu vou separar em dois fatores, um da minha relação com o estudante, eu acho que houveram tentativas de desafiar essa minha posição de poder e gerar algum constrangimento, então eu gosto de contar essa, era a minha anedótica, eu pedi para os estudantes, eu dou aula de administração, de gerencial em enfermagem, entre outras coisas, mas essa situação foi nessa disciplina, e eu pedi para que eles respondessem a itens do processo de trabalho pedindo que eles escolhessem qualquer profissão, qualquer atividade de trabalho que não fosse relacionada a área da saúde, aí eu vi um grupinho de cochicho, né, e aí eu cheguei e falei, ah, tá, o que vocês escolheram, aí o estudante olhou bem para mim assim, nós queremos falar de garotas de programa, eu entendo, eu olhei e falei, vamos lá, é possível responder as perguntas, tem função social, era as perguntas, né, tem função social, tem métodos e instrumentos, e foi trabalhando isso com eles, eu não considero uma violência, mas eles queiram me desafiar e me constranger, né, porque já haviam tido questões dentro da faculdade relacionadas a costumes, né, então era, sabe, era uma provocação para ver até onde eu ia, e essas questões relacionadas a costumes pelo o que eu soube provocaram atritos, provocaram questões muito delicadas entre estudantes e professores, então eu percebi que aquilo era, ele estava me chamando para briga sutilmente, né, então eu não considero uma violência, mas eu considero que por conta da condução não foi violento, mas se eu tivesse dito, isto é um absurdo, né, ou isso não abe aqui, coisa que eu não diria porque não acredito nisso, eu acho que não tem esse tipo de tabu, né, não tenho esse tipo de tabu, mas se eu dissesse isso, né, talvez isso poderia gerar uma situação conflituosa, mas a maioria das situações que... por isso que eu digo que não, sabe, houveram chamadas para o conflito que eu não aceitei, justamente porque eu brinco que o adulto sou eu, né, então [Risos], o adulto sou, né, sou eu que preciso cuidar disso, porque muitas vezes, eles também são adultos, mas eles têm uma maturidade que é diferente, hoje eu tenho uma história de vida que é diferente, eu já fui estudante também, eu já fui provocativa, que é de mim ser provocativa, mas foram situações com os estudantes que foram disso, de chamadas para o conflito, de questionar o que eu estava dizendo, mas que eu acho que faz parte, faz parte desse, em especial na universidade, e se a gente quer ter estudantes críticos eu acho que isso faz parte, pouquíssimas situações de desrespeito, pouquíssimas, né, assédio, assédio sexual, né, não chegou a ser isso também, mas tinha um estudante que era insistente, que me chamava para sair e etc, e um dia ele fez isso em público, e aí eu dei a resposta em público cessou, né. Mas não sei se existe isso, estou pensando aqui agora Angela, se violência também é o que a gente considera ser violento não sei, porque eu não considero essas situações de violência porque não me afetaram, para mim são situações que podem gerar conflitos, mas que se minimizadas não geram. Então respondendo a tua pergunta, eu não considero que eu tenha sofrido nenhuma situação de violência na relação estudante/professor, entretanto, no começo da minha carreira em outra instituição eu considero que na relação de trabalho algumas situações foram de violência moral, então você está vendo, é o meu companheiro passando aqui atrás [Risos], sofri situações de violência moral, né, tais como, cobranças, se a minha correção de prova estava no rigor adequado, e não é no sentido pedagógico, era no

sentido de você está fazendo direito o que você fez, eu acho que tem uma distinção, né, aí do que é um aconselhamento pedagógico e do que é um assédio, né, então eu já fui assediada nesse sentido do que eram as minhas práticas pedagógicas pensando em especial em... era uma instituição privada que prezava pela suas notas externas, né, então já sabemos do que eu estou falando, né, então tipo, a sua prova é complexa ao bastante, a sua correção é criteriosa ao bastante, coisas nesse sentido, isso era bastante estressante assim. Eu fui fazer terapia, a gente fazia um check-up anual e tinha psicólogo, até a instituição que fornecia, e no meu teste de stress estava altíssimo por conta destas situações, né, e que claro isso foi uma das coisas, mas tinha salas com turmas, eu tinha 120 alunos numa turma, e várias turmas de 120 estudantes, então era um volume de trabalho alto, eu tinha uma carga horária alta, eu acho que muito nesse sentido assim de, ah, na mesma instituição, mas eram várias unidades, né, numa das unidades a coordenadora dava razão total para o estudante sem antes entender o contexto, e novo, tem situações em que o estudante tem razão, não e que não tenha que dar razão para o estudante, né, mas eu acho que isso precisa ser analisado, qual foi o contexto, qual foi o outro envolvido, tem o que é dizer sobre isso, e aí você entende o contexto e toma atitudes, mas isso enfraquece muito o docente quando o estudante que além de estar pagando sabe que se ele for a uma coordenação a razão será dele, e aí isso começa a ser uma ameaça, começa gerar ameaças, assim, não permitia, cortava na raiz, eu brincava aqui não meu querido, aqui não, comigo não, mas isso gera um cansaço, um stress, quando acontecia alguma situação conflituosa em sala de aula eu já tinha que ir correndo contar, sabe que nem quando você faz malfeito quando é criança você corre contar para a mãe, né, era quase isso, e eu acho que isso é um cerceamento da liberdade do docente, e que você sabe bem disso, é importante que o docente tenha claro que dentro das normas, do local em que você trabalha, do projeto pedagógico, claro que dentro de todo um contexto a gente precisa ter um pouco de liberdade, se sentir tranquilo para fazer o trabalho, e aí eu acho que pensando nisso foram coisas nesse sentido que eu considerava violento, eu vomitei já de stress nestas situações, né, então fina do semestre para mim era terrível assim, eu corrigia setecentas provas a cada duas semanas, né então era assim, um volume de trabalho importante, não que o volume de trabalho seja violento, mas eu acho que juntando isso com turmas grandes e com esse contexto todo receber uma chamada de atenção do seu coordenador por algo que você julga estar fazendo certo para mim, e o motivo foi assediador para mim, então eu senti como assédio, então a violência que eu sofri foi assédio moral, né.

Entrevistadora: Patricia, é qual foi a sua atitude em relação a essa situação vivenciada?

Entrevistada: Foram duas na verdade, a minha avó disse que eu não tenho muita parada, agora que eu estou parada porque eu passei no concurso público, mas o meu movimento, foram dois movimentos na verdade, um deles foi nesse ápice foi dizer, não faço isso, eu trabalho assim, não vou trabalhar dessa forma que você está me exigindo, eu vou cumprir com as minhas obrigações docente, mas o meu limite é esse, então eu consegui estabelecer isso, mas isso Angela depois de tempo, de terapia, enfim, eu acho que esse foi um movimento. Um segundo movimento e que eles alteravam a nossa carga horária de um semestre para outro sem avisar, e aí teve uma reunião que eu falei, virei para a coordenadora, falei assim, olha, vocês precisam ter o mínimo de

garantia de carga horária para a gente porque eu não sei se eu pago o meu aluguel, e aí a partir disso, né, eu fui buscar outras coisas, e eu troquei de emprego, eu pedi uma licença desse emprego e fui trabalhar em outro lugar que também tinha serias questões, mas que não era na prática docente, era com educação permanente, que é o que eu estudo, e aí fui para lá, mas eu voltei para essa instituição, eu fiquei ainda um ano e pouco, eu voltei, quando eu terminei o meu doutorado eu voltei, mas aí eu percebi que a minha postura era diferente, então eu voltei de zen, eu assumo, tal carga horária, eu assumo, disciplinas que tenham a ver com o meu campo de conhecimento, porque eu dava de A a Z Angela, eu só não dava disciplina que tinha, que fosse instrumental hospitalar, porque aí para mim era demais, né, eu não tenho.... Clínica médica, clínica cirúrgica, UTI eu não transito bem nesse aspecto , em especial porque eu estudo gerenciamento, né, o que tangia a saúde coletiva eu também abraçava, por conta da minha experiência profissional, mas eu consegui delimitar, eu dou disciplinas relacionadas a saúde coletiva e gerenciamento, então delimitei, delimitei carga horária, e aí eu consegui trabalhar com prazer, tinha dia que eu trabalhava, entrava as sete da manhã e saia dez e meia da noite, existiam esses momentos, mas eu conseguia fazer isso de uma forma mais tranquila e fiquei só na unidade que a coordenadora não era... ela fazia o diálogo com o professor para entender o contexto, né, então eu consegui fazer isso, né, eu acho que foi a minha forma, mas levou um tempo viu Angela para eu conseguir estabelecer um pouco esses limites, sabe.

Entrevistadora: Entendo. Professora, no seu entendimento essa formação profissional aí dos formandos da área de saúde, a temática “Violência Contra Professor” ela está contemplada nos conteúdos mínimos dos projetos pedagógicos?

Entrevistada: Não, eu acho que tem coisas que perpassam, mas que são muito amplas, então está no conteúdo ética, por exemplo, né, então em tese haveria alguma relação, mas violência nessa relação não está contemplada, e diria Angela que eu não sei em profundidade dentro das disciplinas desse currículo da universidade que eu estou agora, mas me parece que a violência passa muito tangencial, sabe, eu acho que na saúde da criança e da mulher meio que passa meio que obrigatoriamente, né, porque são temas que são relevantes, violência obstétrica está aí, violência contra a criança também está aí, mas eu não sei o quanto isso é aprofundado, não sei te dizer a profundidade, eu sei que existe, mas não sei a profundidade, mas eu acho que violência de modo , eu acho que violência ampla é pouquíssima coisa que é vista, talvez um pouco mais focada nessas duas, né, que e na saúde da mulher e na saúde da criança, mas eu acho que esse diálogo, né, entre... das relações professor/estudante ele não está no currículo, talvez porque não tenhamos mais licenciatura, então eu acho que na licenciatura talvez isso pudesse aparecer porque trata-se da relação, da relação pedagógica mesmo, mas algumas coisas muito, eu acho que vem muito mais das falas dos professores quando percebem situações deste tipo, então eu estou aqui me lembrando de algumas falas dos professores diante de algumas ações de estudantes que foram desrespeitosas, por exemplo, né, então... eu tenho uma amiga que fala, só um pouquinho [Risos], só um pouquinho, espera aí, né, porque eu acho que tem um pouco... eu estou fugindo um pouquinho da pergunta, mas eu acho que isso é importante, eu acho Angela que o professor, em especial na universidade tem um desafio que é, como é que a gente forma pessoas críticas, porque isso está no nosso PDC, está nas DCNs, como é que a gente forma pessoas críticas que possam ter voz,

que possam se posicionar, que possam discordar, só que a gente tem que formar o como fazer também, né, porque por vezes os estudantes confundem posicionamento com desrespeito, né, então posicionamentos fortes com ataque direto a pessoas, então a gente está um pouco nesse momento Angela, eu sou do NDE, né, eu acho que isso me identifica um pouco, mas enfim, eu sou do NDE, e a gente tem discutido isso, como é que eu estímulo que eles se posicionem, porque é isso que a gente espera que eles façam, a gente não quer ninguém subalterno, mas como que a gente estimula que eles façam isso de uma forma que não seja fofoca, movimentação e de repente taca tudo na cara assim, diálogo, subir as coisas para instâncias, outras sem trabalhar isso internamente, às vezes são coisas que uma conversa interna resolveriam, né, como é que a gente faz essa formação, né, então eu acho que a sua pesquisa vai poder nos ajudar com isso, mas eu acho que isso é um desafio importante, né, porque por vezes me dá vontade de falar tipo, chega sou eu que estou falando, eu sou autoridade aqui, ainda bem que eu nunca disse isso, porque isso seria uma marca no jeito que eu penso a postura docente, né, mas eu confesso que por dentro às vezes me, tipo, chega sabe, por dentro dá vontade de dizer chega, mas aí você vai lá, por isso que eu disse um pouco do preparo, eu fiz licenciatura, e eu acho que isso é importante, muitos professores não passam por essa etapa da licenciatura, porque isso é opcional, eu fiz opção de fazer licenciatura justamente para entender um pouco melhor o que é didática, qual é a importância do projeto político pedagógico, porque o mestrado não nos prepara para isso, por mais que a gente faça preparação docente é uma coisa que é ampassã, o mestrado é vertical, a gente está aprendendo a fazer pesquisa, e o nosso foco é fazer pesquisa, então a gente vai buscar, não é, a gente vai buscar as disciplinas que me tragam esse embasamento para a minha pesquisa ficar bacana, né, o doutorado ainda mais, porque o doutorado a gente quer ser pássaro livre, né, a gente [...] um pouquinho do orientador e também vamos buscar isso, não passa na mente que o nosso tema seja educação, não, entendeu, eu não percebo isso, no meu mestrado, eu estudo educação permanente, então eu fui para a faculdade de educação, eu fui para faculdade de psicologia, né, fazer disciplinas lá, eu fiz só as obrigatórias na unidade que eu era... que eu estava matriculada, fiz esse combinado com a minha orientadora, eu falei, eu vou fazer só as obrigatórias que foram duas disciplinas no mestrado e duas no doutorado, todo o resto da minha carga horária foi fora, foi na psicologia, foi na educação, foi na administração, porque eu queria ter um pouco mais dessa, uma visão um pouco diferente, então eu aprendi coisas, sei lá, fiz uma disciplina que fala de poder, porque eu fiz uma disciplina que chamava educação e poder, que trata dessas coisas também, das micro violências, né, dessas reações de poder, então eu acho que isso me deu um pouquinho de preparo, mas ainda sim considerando que eu tive um pouquinho de preparo por ter feito licenciatura, por ter participado de discussões relacionadas a educação, ainda sim me falta algumas coisas, né, ainda falta porque eu acho que a gente, o professor universitário ele tem que brotar professor rapidamente, ele ganha o título, ele passa no processo seletivo no concurso ele virou professor, né, quando em outras, outras profissões para você lecionar você precisa ter licenciatura, né, complicado isso, delicado, né, não sei se eu seria a favor de que todo professor fizesse licenciatura, não sei Angela, estou aqui só trazendo contextualização, mas a gente chega despreparado, né, chega despreparado, e aí trazendo mais uma pitada, a gente tem uma geração que e diferente, a gente não aprende nada sobre gerações, né, então tem coisas que a gente ler como descompromisso que não são, né, tem coisas que a gente ler como desrespeito que na cabeça da pessoa pode ser desrespeitoso,

mas para aquela outra geração aquilo não é desrespeitoso, porque ele tem um diálogo de igual para igual com os pais, com os professores desde de sempre, e aí chega na universidade ele quer manter isso, e aí isso é um pouco mais antigo você fala, como assim, não está me chamando de senhora, né [Risos].

Entrevistadora: Então professora Patricia, essa sua fala vem muito ao enfoque agora da minha pergunta que diz, olha, então o que você considera violência contra o professor? Veja, a minha primeira pergunta você falou, olha, está amplo, né, e agora a gente está delgaçando, afunilando aí. Para o professor o que literalmente você considera uma violência contra professor?

Entrevistada: Olha, eu acho que eu consigo dizer um pouco melhor da questão do processo de trabalho, então eu considero que não ter um ambiente, a gente sabe da importância do ambiente para educação, e eu acho que a gente não ter condições de trabalho, e aí estou dizendo de forma ampla, espaço físico, equipamentos, recursos, sabonete para lavar a mão no banheiro e papel higiênico, que a gente sabe que em alguns espaços nem isso existe, estou num espaço que é privilegiado, uma questão de organização do processo de trabalho, né, então na universidade gente tem que dar conta de ensino, pesquisa, extensão e questões gerenciais também, né, questões administrativas, então uma organização do processo de trabalho, a depender de como isso acontece pode ser violento, pode gerar assédios, pode gerar desgastes, então eu acho que isso é uma questão. Em relação ao estudante possibilidades de violência, né, difamação, calúnia, né, e a gente sabe que hoje tem rede sociais que fala, gravar uma aula sem autorização, gravar a minha voz sem que eu autorize, né, eu acho que são, não sei se eu posso chamar de violência sutis, porque quando a gente pensa em violência parece que o auge é levar um tapa na cara, né, mas o quanto que uma difamação pode prejudicar um professor, né, uma carreira, né, o quanto isso é uma marca na carreira que sai com menos facilidade do que o tapa na cara que você levou, né, então eu acho que pensando nisso, né, acho que essa questões de calúnia e difamação, exposição nas redes sociais, né, essa quebra de confiança, que se a gente combinou que não vai gravar e você está gravando, né, isso é sério, as pessoas não entendem que isso é sério, e não entendem que no contexto que a gente está você pode receber uma intimação, né, pode receber uma intimaçãozinha, então, assim, eu acho que passa por isso, e aí claro violência física, mas isso está muito distante do meu contexto Angela, eu acho que, eu estou num lugar que privilegiado, né, então... mas eu sei qual é descrição da educação pública, da educação infantil, eu tenho um colega que foi sequestrada, né, foi sequestrada, porque ela estava num espaço que era dominado por uma facção criminosa e eles entenderam que ela estava afrontando, eles sequestraram ela e fizeram o julgamento, ela foi para o tribunal, então, assim, não foi em Goiás, em um outro espaço, né, mas foi uma colega que eu soube que aconteceu isso, então... quando a gente pensa nesse nível de violência, né, mas só trazendo que pode acontecer, no meu contexto as coisas estão mais nestas questões morais, morais e psíquicas de violência psicológica, eu acho que elas estão mais, eu consigo enxergar melhor estas duas, porque nesse contexto que eu estou desconheço violência física.

Entrevistadora: E você desenvolve experiência na sua prática docente que visem o respeito a toda comunidade acadêmica. Porque na sua fala você fala desta violência, inclusive acadêmica, entorno da academia, né, ela está permeando por esse... alguma prática?

Entrevistada: Olha, intencional, sistemática, eu acho que a palavra é, com intencionalidade Angela de saber, está prática aqui vai ser feita para isso, não, eu acho que com a intencionalidade não, o que acontece é, tem uma situação xis a gente resgata e problematiza um pouco, mas depende, depende se aparecer, então não é uma coisa que você chame para o diálogo, né, mas eu tenho percebido que cada vez mais isso tem se tornado importante, justamente por conta daquilo que eu vinha te dizendo, a gente quer uma formação crítica, mas a gente também precisa debater o como, e o como se posicionar passa pela relação estudante/professor, né, de novo considerando isso uma relação de poder, né, então eu tenho percebido e pensado sobre isso, e eu acho que isso vai ter que ser uma discussão dentro da universidade, e eu acho que daí a importância da tua pesquisa quando me disseram do tema eu falei, pode dar o meu nome, eu vou participar, eu acho que é uma...

Entrevistadora: Que coisa bacana.

Entrevistada: Coisa bastante importante que pode trazer contribuições bastante relevantes, porque é uma coisa que como, não sei se como toda a violência, mas parece que a gente tem sempre um momento... Você está ouvindo um barulho é a minha bebê batucando coisas [Risos]. Eu acho que como toda a violência passa por um momento de fingir que ela não existe, né, deixa lá, isso não existe, porque é uma coisa que é, que se eu mexer nisso pode ser perigoso, e é o tipo de coisa que quando a gente mexe a gente precisa mudar, então, não dá para começar a discutir... Angela eu lembrei de uma coisa.

Entrevistadora: Sim.

Entrevistada: Lembrei, teve um momento que foi sistemático, que foi intencional, que foi para ouvir um pouco os estudantes em relação a coisas de evasão, evasão e retenção dentro da universidade foi um evento que a gente promoveu, não foi intencional da violência, mas apareceu como demanda, tá, lembrei disso porque eu acho que isso é legal, apareceu como demanda a questão da relação professor/estudante, né, de alguns alunos, e aí não só da nossa unidade, mas dizendo da formação total da nossa unidade, que abandonaram porque não dava, com aquele professor ou com aquela professora não dava para continuar, sabe, e aí trouxe um pouco dessa relação, e acho que isso entrou um pouco no radar, né, então lembrei isso só para dizer que isso está no radar, mas que isso ainda não virou nenhuma ação efetiva, né, algumas pequenas ações, e aí pesando mais no nível da unidade agora , espaço aberto de diálogo com os estudantes, um fluxo de comunicação dos estudantes com a direção, com a coordenação de graduação, participação dos estudantes no conselho diretor, que é um pouco para melhorar essa comunicação que pode levar uma violência, mas ainda está, sabe assim...

Entrevistadora: Sei, entendo.

Entrevistada: É o caminho.

Entrevistadora: É. Patricia olha só, você, está tão assim fluída a conversa, o diálogo, né, a entrevista que a gente faz aqui uma conversa, e você respondeu duas das minhas últimas perguntas inclusive [...] você já ouviu, e você me relatou uma assim assustadora para algumas pessoas, mas para quem estuda violência [...], mas diferente tudo, né, eu

tenho estudando. E aí completa exatamente a possível estratégia de enfrentamento de violência para os alunos contra o professor. Então é riquíssimo, amei muito aqui.

Entrevistada: Aí falo mais [...] Angela. Só localizando um pouquinho aquela situação de violência foi na educação infantil, tá, não foi no campus da universidade, mas como a gente está falando de prática docente e ampla me ocorreu isso, porque para mim aquilo é uma situação chocante e extrema, né.

Entrevistadora: Com certeza.

Entrevistada: Mas... Ah, eu gostei também Angela, gosto de falar essas coisas, a gente precisa pensar mais sobre isso, não só pensar, precisa pensar primeiro para planejar, mas eu acho que a gente precisa agir mais sobre estas questões, né, a gente precisa...

Entrevistadora: Você acha professora que ficou alguma coisa que você gostaria de complementá-la, e aí assim, talvez não foi contemplada aqui no nosso diálogo, nas perguntas?

Entrevistada: Não eu acho que, acho que conseguir entender a tua linha de raciocínio para chegar nos entendimentos, né, eu acho que só gostaria muito, eu sei que provavelmente você fará isso, mas uma divulgação aproximada dos resultados da tua pesquisa, porque eu acho que a gente tem carência de discutir sobre isso, né, a tua pesquisa é num cenário que tem colegas meus professores da nossa unidade que estão participando, então vão trazer coisas também que são da especificidade, embora isso possa ser ampliado, né, então eu acho que não sei, em algum momento no teu final da gente poder ter esse espaço de discutir, porque eu acho que o que a gente carece é de estratégias, né, estratégias para o diagnóstico, então entender se isso existe, como existe, que eu acho que é um pouco o que você está fazendo, né, e a gente precisa ter estratégias para lidar com isso, né. Eu estou estudando um pouco de comunicação não violenta, porque eu acho que isso minimiza um pouco, minimiza não, você cuida , você consegue entender quando uma conversa pode descambar para um final ruim, né, e aí você consegue, né, retomar, respirar e haja terapia, mas você consegue retomar e respirar, mas a gente precisa de estratégias que não sejam individuais, então a gente... eu acho que é isso, na verdade não é algo que faltou, é um pedido que a gente possa conhecer a tua pesquisa, e acredito que você já tenha planejado alguma coisa nesse sentido.

Entrevistadora: Verdadeiramente isso está planejado professora, essa devolutiva ela tem que acontecer, e verdadeiramente é nesse veio, caminhando aí por essa estrada, né, a estrutura de... a construção de estratégias para nos subsidiar, farei isso com muito prazer, é realmente muito importante quando a gente faz a entrevista e aí a gente vai, à medida que você foi falando ei fui fazendo todos os links, porque muito, muito bacana. Quero te agradecer imensamente, dizer que muito importante para mim você aceitar, sei que nós estamos num momento, estamos assim assoberbados de tanto trabalho, e você achar na sua agenda um minutinho para me atender, muitíssimo obrigada viu, muitíssimo obrigada.

Entrevistada: Eu agradeço a oportunidade, gostei de conversar com você, a tua condução foi leve, eu sou falante, né, que bom que eu já fui cobrindo [Risos] as perguntas que você tinha planejado, e eu acho que a gente precisa arranjar tempo

Angela, eu sou Doutora, eu considero que eu ainda sou jovem Doutora, eu fiz quatro anos de doutorado esse ano, então eu ainda lembro como é [Risos], eu ainda lembro, eu acho que eu não devo esquecer de como é isso, e do quanto é importante a gente, quanto tema é um tema super relevante para nossa prática, e a gente tem que achar espaço, né, porque também já fiz pesquisa, continuo fazendo pesquisa, e a gente sabe o quanto é difícil, em especial nas estratégias qualitativas, não é Angela de você ir um a um ainda, né, esse trabalho é artesanal que e feito, então sempre que eu puder eu vou colaborar nesse sentido, e achei o teu tema muito importante, e chegou assim, Pati tem uma pesquisa que está acontecendo que está super legal, são perguntas interessantes que são importantes para a gente refletir, posso dar o seu contato? Pode [Risos], então...

Entrevistadora: Que maravilha professora.

Entrevistada: Chegou desse jeito Angela, um feedback positivo de como foi [...] tá.

Entrevistadora: Isso é um estímulo e um gás, oxigênio para pesquisar a dor aqui, né [Risos].

Entrevistada: E eu gosto de falar também, tipo olha, está indo no caminho, num bom caminho, quem está participando está confortável em te responder as perguntas, tá bom.

Entrevistadora: Que coisa boa. Tá, obrigada professora, um beijo aí em você...

Entrevistada: Até mais, boa sorte, bom trabalho.

Entrevistadora: E obrigada, até mais.

Entrevistada: Até mais, tchau.

00:42:38

PATICIPANTE 8

Tempo de gravação: 00:18:55

Entrevistadora: Todos os professores que entram aqui fazer a gravação, o termo de consentimento livre, eu só peço que você fale as iniciais do seu nome, a data, o horário e que você aceita a gravação porque aí eu anexo, não preciso ficar lendo todo ele, tá bem?

Nathalia: Certo. Eu aceito a participação, a gravação, as minhas iniciais é NDA, a data de hoje doze de março de dois mil e vinte e um, dez horas e dez minutos.

Entrevistadora: Muito obrigada professora. Então vou iniciar aqui com as nossas perguntas, gostaria de ir levando, professora, numa conversa, porque o qualitativo é sempre esse momento para estar direcionando aqui [...] eu já arquei outras atividades aqui, comunidade, brasileira, mas qual o seu estado, esse para o perfil psicográfico, qual o seu estado civil?

Nathalia: Eu sou casada.

Entrevistadora: E o maior grau de instrução de titularidade?

Nathalia: Doutorado.

Entrevistadora: Quanto tempo, professora, você ministra aula?

Nathalia: Desde... em todos os níveis de educação ou só ensino superior?

Entrevistadora: Pode ser em todos os níveis, assim, você tem mais de quinze anos, menos.

Nathalia: Desde dois mil e nove.

Entrevistadora: Então agora as perguntas mais subjetivas, a temática é violência, então partindo da palavra violência qual que é o conceito que você poderia, colocaria nesse momento sobre violência?

Nathalia: Em geral, Ângela, ou no contexto do meu trabalho?

Entrevistadora: Em geral.

Nathalia: Em geral. Violência eu entendo como qualquer tipo de agressão não necessariamente física, mas sendo a física como a maior extrapolação de violência, então eu entendo violência como aquela que é cometida de modo verbal, quando você subjuga uma outra pessoa, quando você a diminui, quando você a desrespeita na sua integridade como ser humano e também a violência física e outras formas de violência física, que existem e a gente sabe também, violência sexual, violência de outras naturezas, mas acho que é muito importante a gente ressaltar formas de violência que não é o primeiro pensamento das pessoas, mas que é essa no relacionamento, né, na questão verbal que não chega a ter uma agressão física.

Entrevistadora: Professora, para você existem fatores que estão desencadeantes para a violência ou da violência?

Nathalia: Eu acho que... nossa, é difícil essa pergunta, Ângela, eu acho que situações de conflito nos relacionamentos elas podem desencadear a violência, agora, para que a violência de fato ocorra eu acredito que tenha um fator muito pessoal daquele que vai de fato ser violento ou ele vai se mobilizar negativamente para uma situação adversa, mas conseguir controlar internamente a sua reação de modo a não ferir o outro.

Entrevistadora: E você já sofreu algum tipo de violência durante a sua prática pedagógica?

Nathalia: Eu considero que sim e eu acredito que ela é, nesse ponto de vista do verbal, eu acho que isso acontece, tem diversos níveis, assim, não chega, muitas vezes, e não é comum que ele extrapole demais, mas ele fica na comunicação, inclusive, escrita, muitas vezes, ele acontece.

Entrevistadora: E qual foi a sua atitude, ou qual é a sua atitude nessa situação vivenciada?

Nathalia: Sempre que eu sinto uma forma de violência ou agressão por escrito ou falado eu sempre me posiciono e o meu posicionamento é sempre de ponderação, de falar mais sobre aquilo que se está querendo dizer, ou do problema que se está em pauta, tirando o foco da forma como foi feito, mas às vezes eu acho necessário falar sobre ele, quando a coisa se resolveu e as pessoas estão mais calmas é sinalizar de que aquela não é a melhor forma de conversar ou de resolver uma situação, então eu sempre tento, Ângela, focar no problema que se apresenta, no conflito que se apresenta para resolvê-lo, porque ele muito provavelmente foi o motivador da violência, e uma vez que está resolvido ponderar com as pessoas sobre a forma de se relacionar evitando que haja uma próxima violência.

Entrevistadora: E, assim, durante a formação, a sua formação profissional, durante esse período que você já ministra aula, essa temática, violência contra o professor ela foi contemplada ou ela está sendo contemplada nos conteúdos mínimos dos projetos pedagógicos?

Nathalia: Não, contra o professor não, nós falamos muito, por exemplo, na área da saúde sobre violência em relação a mulher, criança, ao idoso, as pessoas em geral, mas nós não discutimos a violência no ambiente educacional.

Entrevistadora: Então, e aí essa experiência, assim, que não está contemplada dentro dos projetos pedagógicos, mas você desenvolve como docente visando o respeito da comunidade acadêmica? Tem um braço aí, assim, uma vertente que está contemplado no projeto, mas eu enquanto professora, você desenvolve alguma...?

Nathalia: Eu creio, Ângela, que, assim, nós não temos um conteúdo que se chama violência no ambiente de ensino, mas nós vivenciamos isso em medidas diferentes e não todos os dias, não intensamente, ou às vezes para algum professor até, não sei, depende muito da posição que se ocupa, na coordenação de graduação vivencia-se mais porque você tem mais contatos com um número maior de estudantes, enfim, agora, apesar de não ter conteúdos formalmente estabelecidos no projeto pedagógico de curso nós temos esse tipo de vivência no currículo vivido, no currículo vivenciado, então esse currículo vivenciado que é permeado pelas relações é possível problematizar esse assunto e eu creio que eu tenho feito dessa forma como eu te digo, então sempre que eu percebo uma forma de violência e que não necessariamente é do estudante para o professor, é de todas as pessoas que estão envolvidas nesse ambiente é possível que seja discutido a melhor forma de se relacionar evitando violência, então ela permeia o currículo, não no currículo formal, não no currículo escrito, mas no currículo vivenciado sem sombra de dúvida.

Entrevistadora: E no seu entendimento, o que pode ou o que você já vivenciou que pode levar o aluno a usar a violência contra o professor durante a prática pedagógica?

Nathalia: O que pode levá-lo?

Entrevistadora: É, o que pode levá-lo.

Nathalia: Como eu te disse eu acho que o ambiente, a situação que pode favorecer isso é o conflito, é quando a pessoa se sente ameaçada ou não contemplada ou quando algum desfecho, algum encaminhamento vai contra aquilo que ela imaginava que

deveria ser ou como ela gostaria que fosse, então acho que o grande motivador é o conflito, eu acredito que os fatores pessoais que tem a ver com personalidade, com a formação dessa pessoa, com o contexto de vida familiar e social dela é o que vai determinar se ela vai agir com violência ou não e inclusive o nível de violência que ela vai empenhar na reação, a sua pergunta, essa eu já tinha falado, mas você fez outra nuance da pergunta, desculpa, Ângela, qual que foi?

Entrevistadora: No seu entendimento o que provoca... uma eu perguntei quais são os gatilhos da violência, agora, especifico para o aluno, o que faz com que o aluno possa usar de violência...

Nathalia: Eu queria falar uma coisa sobre isso, que me passou pela mente, eu queria fazer esse contexto primeiro, eu acho que é o conflito, mas eu acredito que nós herdamos um modelo muito tradicional de educação, então existe uma vivência, nós e os nossos estudantes ainda, que são bem mais jovens, de uma... e eu não estou falando de autoridade, estou falando de autoritarismo, então por vezes, por conta desse modelo tradicional de educação há essa relação e o estudante se sente oprimido, e quando ele tem uma oportunidade de confrontar uma liderança que no caso é a figura do professor ou da gestão da escola ela age com violência porque ele já sofreu algum tipo de violência ates, então eu acredito que isso está imbricado, muito imbricado no processo educacional pela herança que nós temos do modelo tradicional de ensino, o Paulo Freire já falava sobre o oprimido e o opressor, então o estudante que a gente tem no curso superior já na fase adulta quando ele faz esse confrontar mesmo que o professor a quem ele confronte não tem essa abordagem autoritária ele pode agir de violência por conta de um histórico que já teve, já na defensiva para tentar se sobressair de uma situação ele foi subjugado anteriormente, pelo menos eu imagino assim.

Entrevistadora: É, certinho. Até hoje você presenciou ou vivenciou alguma violência proferida de aluno contra professor que mais te impressionou, que possa ter te impressionado?

Nathalia: Deixa eu pensar um pouquinho, Ângela.

Entrevistadora: Porque você fala de violência educacional, aqui o professor inserido, alguma coisa que te chamou a atenção.

Nathalia: Nossa, Ângela, eu já vivenciei algumas, em especial na coordenação de graduação eu tenho visto isso mais intensamente, quando você tem algum papel de gestão você tem mais vivências, então você tem uma mostra maior do fenômeno, né?

Entrevistadora: [Risos] Sim.

Nathalia: Eu já tive situações, seja comigo, ou seja com colegas, em que dá um nível de ansiedade muito grande pelo nível do conflito, eu não consigo, olha eu coisa, claríssimo para te falar o que aconteceu, mas eu já vivi situações, e isso é muito complicado, quando o estudante expõe um determinado professor diante de um grande grupo de estudantes e professores, e isso é muito delicado porque geralmente o professor não está lá também para fazer a sua fala, para se defender, então cria toda uma situação que eu acho que demora muito para esclarecer e resolver e que não e

justo, não é correto, então, assim, nas relações de trabalho nós já temos isso muito claro, s tem alguma situação de conflito você antes de fazer algum tipo de mediação você faz uma abordagem direta para ver se resolveu, se o conflito é muito grande você procura um mediador, mas muitas vezes estudantes talvez não entendem os fluxos das relações de trabalho para tentar fazer isso da forma mais branda para ver se melhora, então por vezes eles usam outros espaços e outras abordagens que não sejam mais resolutivas e que consigam de uma forma mais pacífica, de toda forma a universidade ela é um ambiente muito favorável porque nós temos como meta o desenvolvimento não só de profissionais de excelência, mas formação de cidadãos, então nós problematizamos esse tipo de situação, Ângela, e eu acho isso fundamental, na minha visão isso é fundamental, para que as pessoas tenham um melhor preparo também para as relações que elas tem na sociedade de maneira geral, não só dentro da escola, né?

Entrevistadora: Professora, nós estamos caminhando já para o final da entrevista, incrivelmente eu acredito que você acabou de responder a penúltima pergunta, que seria, quais são as possíveis estratégias de enfrentamento a violência dos alunos contra professor?

Nathalia: Eu sempre defendo, Ângela, que é a problematização dessa situação e a conversa, a comunicação franca, usados espaços para que os estudantes possam ver e eu poso te afirmar já que eu vi melhoras, eu já vi muitas melhoras, eu estou na coordenação há um ano, embora pareça muito mais tempo por conta da pandemia, porque as coisas foram mais difíceis agora do que geralmente, e nós tínhamos algumas vivências, assim, que percebia-se até um desejo de confronto muito grande dos estudantes e por isso eu te disse em outra resposta que eu creio que é por conta de um histórico de vivências anteriores, e aí não importa quem está resolvendo com eles qual é o seu desejo de advogar por eles, eles gostariam de se confrontar ou de se colocar de uma forma mais impositiva, e nós temos uma situação totalmente diferente hoje, e a minha visão, e eu vejo isso como um caso de sucesso, é você problematizar e buscar resolver, não ignorar o problema, não ignorar o possível conflito, mediar o conflito e com tranquilidade, com serenidade, tentando resolver o problema, explicando as situações, as possibilidades, os rumos, trazendo isso de uma forma democrática, fazendo uma escuta ativa, Ângela, escuta ativo dos estudantes é fundamental, então, assim, essa relação que permeia a morosidade, então, assim, tem estudiosos que falam sobre isso na área da educação, então quando você fortalece a relação professor-aluno baseada no respeito, baseada da construção os efeitos são surpreendentes, são muito positivos e isso é o que eu falo quando a gente está falando de formação do cidadão, então, assim, eu consegui viver isso, e isso é, para mim, motivo de muita felicidade, e nós professores ficamos com esse intuito o tempo todo, da formação do cidadão.

Entrevistadora: Muito bom, muito bom mesmo. Professora, ficou algo que você gostaria de complementar, aqui, estou encerrando, que talvez não tenha sido contemplado e que você sentiu necessidade de colocar?

Nathalia: Obrigado por perguntar, Ângela, ficou, [Risos]. Eu já fiz muitas reflexões nesse sentido, por isso eu queria muito participar, quando você fez o convite fiquei muito feliz e achei aqui uma agenda porque eu acho esse tema importante, eu já ouvi professores meus, mais velhos, em momentos anteriores da minha vida falando sobre diferença de gerações nas relações entre professor e estudante, eu acho que hoje, mais velha, e

olhando para meus estudantes mais jovens, eu consigo entender essa fala, eu acho que ela não deve estereotipar tudo, porque tem as questões pessoais, como eu falei, mas eu acredito que as relações mediadas pela informação rápida dos aplicativos, das frases curtas, elas tem gerado alguns problemas de comunicação, então, assim, por vezes eu recebo mensagens de estudantes, e e-mails, que eu me sinto desrespeitada, pode ser que não seja a intenção deles, e eu sei que muitas vezes não é, porque os conheço em outros contextos, então se eu vejo pessoalmente a conversa que nós temos é muito respeitosa, se eu recebo uma mensagem a minha forma de leitura foi um desrespeito, só que, assim, tem vezes que é de fato e tem vezes que não, então eu acho que esse cuidado com a mensagem escrita, porque a comunicação não verbal é muito importante, e a gente não tem ela na mensagem escrita, ela é importante para evitar situações de conflitos, aqui a gente não está falando só de violência, né, mas a violência pode começar a partir de uma situação dessas, então eu acho que com o advento dos aplicativos, a informação rápida, a gente está em reunião o tempo todo, online agora, e tem mensagem o tempo todo de estudante, professor, que o cuidado no escrever a mensagem é fundamental para evitar situações de conflito que às vezes são absolutamente desnecessárias ou não era intenção da pessoa, né?

Entrevistadora: Certo. Professora Nathalia, muito obrigada, assim que eu concluir eu acredito que resultará, assim, essa tese resultará em coisas muito importantes para que a gente reflita sobre essa violência educacional. Eu agradeço muito você ter aceitado esse convite, ter encontrado lá no spam e se prontificado a estar aqui numa agenda espremidinha aí para que pudesse me atender, muito obrigada, viu, professora?

Nathalia: Eu que agradeço a oportunidade, Ângela, foi ótimo. E aí eu quero ver os resultados da sua pesquisa depois, os frutos dela, aí você compartilha, por gentileza.

Entrevistadora: Compartilho, com muito prazer, obrigada, professora, eu vou encaminhar então, depois, a listinha, e assim que tiver...

00:18:55

PARTICIPANTE 9

Tempo de gravação: 00:31:08

Entrevistada: Termo de consentimento livre e esclarecido [Risos].

Entrevistadora: É, isso mesmo, e a data de hoje.

Entrevistada: Hoje, dia 31/03/2021.

Entrevistadora: Isso, muito obrigada. Eu vou fazer aqui aquelas perguntinhas básicas, porque é qualitativo, mas está no perfil profissiográfico, e aí eu vou pulando alguns dados que eu já sei para a gente otimizar no tempo, mas aí aqueles que não aí se você puder colaborar comigo fica à vontade, tá bem.

Entrevistada: Tá bom.

Entrevistadora: Vamos lá iniciar aqui professora, eu gostaria de saber qual é o seu estado civil?

Entrevistada: Casada.

Entrevistadora: E qual a sua titulação, a sua maior titulação?

Entrevistada: Doutorado, Doutora.

Entrevistadora: Doutora. E há quanto tempo você é professora?

Entrevistada: Espera aí, nós estamos em 2021?

Entrevistadora: É.

Entrevistada: Já tem uns dez anos.

Entrevistadora: Dez anos, certo. Então, para iniciar a nossa conversa eu pergunto para você...

Entrevistada: Eu acho que já são uns dez anos.

Entrevistadora: Certo. Eleonora qual que é o conceito de violência para você?

Entrevistada: Bom, para mim violência é qualquer coisa que atente a saúde de uma... Seja ele mental, seja ela física, seja ela emocional, então ela pode ser feita por qualquer pessoa em qualquer circunstância, e que vai uma pessoa de algum modo, e isso para mim tem repercussão direta na saúde dela, então é uma coisa ligada a [...] tanto na saúde emocional, física, social, né, que atrapalhe ela e a saúde dele em alguma medida.

Entrevistadora: Certo. E para você...

Entrevistada: Eu acho que parte desse princípio aí, e todos estão expostos a isso, todos estão expostos a sofrerem violência e a serem agressores, né, é bem importante ressaltar isso, porque a gente quando trata da violência ou conversa sobre ela a gente tem muito aquilo de cinismo, né, mas em muitas ocasiões a gente tem a mesma chance de ser vítima e de ser agressor, então basta a gente saber como agir, né.

Entrevistadora: Verdade. E você acredita quais seriam alguns fatores se é que existem para desencadear a violência, fatores que podem desencadear a violência?

Entrevistada: Você pode só repetir Angela, que deu uma travadinha aqui. Então fatores que podem beneficiar a violência?

Entrevistadora: É, se você visualiza, pode exemplificar ou se tem fatores que desencadeiam a violência?

Entrevistada: Bom, eu acho que os fatores que desencadeiam a violência eles são de cunho cultural, [...] então, eu acho que baseado na cultura, né, na sociedade que você vive, no tempo que você está vivendo existem alguns conceitos ou normativas sociais, morais, culturais que podem desencadear a violência, então vamos dar um exemplo, a gente está num momento em que infelizmente ainda a mulher é submissa em vários aspectos em relação ao homem, né, devido à nossa [...] é bem machista, patriarcal,

masculina, então isso torna a mulher suscetível situações de violência, de desencadear a violência, isso vem mudando, mas também vejo na criança, né, a criança ela é desvalorizada, né, então na nossa [...] quem ver é aquele que é ativo financeiramente, enfim, então essas suscetibilidades, né, então eu acho que os fatores de risco para a violência eles têm uma ligação muito forte com a cultura, com o meio social, moral, independente, eu dei alguns exemplos, mas todos eles, né, então se a gente pensar numa briga de trânsito, se a gente pensar numa violência doméstica, numa violência social, baixo salário, de vida, então tudo isso vem por fatores que estão assim, eles não estão perceptíveis, a gente tem que fazer uma forcinha assim para querer enxergar, é tão rotineiros que se tornam naturais na nossa vida, mas [...] a nossa sociedade, a nossa cultura, o nosso jeito de viver.

Entrevistadora: Ok. E você já sofreu algum tipo de violência na sua prática pedagógica?

Entrevistada: Ah, já, vários.

Entrevistadora: E que tipo?

Entrevistada: Oh, eu já tive do tipo ameaça mesmo, de ser ameaçada, do tipo assim, cuidado com que pode acontecer com o seu carro, cuidado que eu estou sempre por aqui, de aluno, já tive pais de alunos me esperando no corredor da faculdade, e isso ser percebido pelos demais colegas que tiveram que sair da faculdade me acompanhando para ser a minha proteção física mesmo, não só pelos alunos, mas pelos pais dos alunos que estavam lá, e esse fato especificamente aconteceu dentro da universidade federal, né, e eu já trabalhei [...] federais ao longo desse tempo, né, então não foi aqui em Goiânia, mas foi nesse outro que eu estive, mas era uma universidade federal [...] para o curso de enfermagem e o motivo era que eu tinha reprovado, a minha orientanda de TCC porque ela comprou o trabalhou [Risos], então, assim, não foi nem o motivo assim, sabe.

Entrevistadora: Entendi.

Entrevistada: Aquilo para se formar, então já tiveram várias circunstâncias desse tipo, além disso, essa eu acho que eu digo assim que foi uma coisa assim mais assim, mais fácil de perceber a violência, mas em sala de aula apresento agressão verbal, né, e a gente considera isso desrespeito mesmo, não é agressão verbal, é a pessoa ficar aqui limitando o seu fazer profissional ali, né, por ameaça, principalmente de agir dessa forma assim agressiva, com palavras, as reuniões, né, as aulas são via remoto, a gente utiliza muito WhatsApp para otimizar atividades prática, atividade teórica, eu acredito que essa geração como não está ali no frente a frente, na cara ali da pessoa eles ficam mais agressivos, então eles utilizam [...] do meio virtual de uma forma muito agressiva, [...] presencialmente ali cara a cara, né, fisicamente juntos, né, digamos assim, então eu percebo que isso vem crescendo muito nos últimos... Eu não sei é um pouco a geração, né, eu não sei se é um pouco a característica dessa geração x, y, z, sei lá qual que é que tá, mas eu percebo que essa forma virtual veio impulsionar isso, principalmente na sala de aula, no relacionamento com o professor.

Entrevistadora: Certo. E qual foi...

Entrevistada: E tem uma outra violência também, eu não sei se vale a pena citar, que é uma violência da gestão, né, então, assim, faculdade federal a gente tem do tipo assim, olha aquele aluno ele [...], aquele aluno ali é um aluno problema, quanto menos tempo a gente tiver ele com a gente melhor, então vamos mandar esse menino formar, vamos [...], então isso acontece muito forte ainda, então é uma pressão você fica pensando poxa, mas se você for pegar assim o que o aluno teve de crescimento intelectual, de habilidades, de conhecimento práticos, muitas vezes não corresponde aquilo, mas você tem aquela pressão para formar o aluno, então pressão para falar não, desconsidera isso, não e uma frequência, não vamos reprovar por essa frequência, enfim, então isso ainda é muito forte.

Entrevistadora: E diante dessa violência que você relata qual foi a sua atitude vivenciada?

Entrevistada: Olha, a minha atitude ao longo desse tempo que eu venho desenvolvendo, porque são coisas que a gente vai aprendendo [...].

Entrevistadora: Verdade.

Entrevistada: O máximo que eu conseguir tornar aquilo formal, então, tipo, se ele está queixoso, que não tem frequência, que não tem isso, qualquer coisa que a pessoa queira de forma agressiva ou não aceito por e-mail, entendeu, eu não aceita conversa de corredor, sempre a gente... nós nunca trabalhamos sós, né, então dentro de uma disciplina são vários, a gente tem um coordenador de disciplina, tem um coordenador de graduação, até a direção, então sempre eu requisito essa hierarquia para tratar com o aluno ou tratar com o [...], eu tenho um problema com o professor, por exemplo, que pode acontecer, então eu vou para o coordenador da graduação, vou para direção, e com o aluno da mesma forma, e formalizar o máximo possível, então [...] por escrito, vamos formalizar da mesma maneira que minha resposta vai ser por ali, evito [...] coisa que eu venho fazendo, e assim às vezes eu fico triste de ter que fazer isso, né, nessa realidade, mas é o que me respalda, apresento o plano de disciplina para aluno com o cronograma, com horário, com prova, com dia de prova, dia de [...], como que vai ser a conduta em sala de aula, tem abaixo, eu Li e conheço e estou de acordo com essas normas, e tem que ter assinatura de aluno, então o que eu faço é me respaldar legalmente e formalmente o máximo que eu poder, porque na hora de um processo, né, na hora de qualquer contratempo ou reação assim violenta de aluno em relação a um combinado então eu tenho como comprovar esse combinado, como comprovar essa agressão, como comprovar tudo, para não ficar aquela coisa perdida, aquela violência que a gente sofre que são perdidas, que não são comprovadas, e às vezes não tem nem condições de você ter um respaldo no sentido de você se sentir em situação de... naquela situação de se respaldar legalmente tanto no sentido que o aluno te coloca como agressor e você não ter esse respaldo, então da minha carreira docente eu venho cada vez mais respaldando com comprovações para que isso se resolva da melhor forma possível caso venha a ter, né, porque isso foi aprendido a longo do tempo, claro que foram várias experiências horríveis para eu chegar no ponto de ter tudo que eu faço enquanto docente ter esse respaldo, legal, normativo, enfim.

Entrevistadora: Certo. E no seu entendimento professora, durante a formação profissional assim da saúde essa temática da violência contra professor ela está contemplada nos conteúdos e nos projetos pedagógicos, você consegue visualizar?

Entrevistada: Não, não, não tem, a gente está com um esforço grande para tratar a violência assim, socialmente, então, assim, lá dentro da disciplina ginecologia obstetrícia a gente fala um pouquinho de violência obstétrica, fala de violência contra a criança lá disciplina de pediatria e neonatologia, contra o idoso, mas essa parte pedagógica é institucional é muito pouco falada, o que está melhorando em relação esses contatos que estão sendo estabelecidos no início das aulas mediante esse novo formato e remoto, então lá a gente coloca questões éticas, questões de plágio, de fotografia, de aula, mas muito sutil, não é um tema assim, não vamos tratar sobre isso, então eu considero que não tem.

Entrevistadora: E na sua opinião, o que você, não é assim, na sua experiência de prática o que você tem desenvolvido para visar o respeito da comunidade acadêmica, no projeto pedagógico não está sistematizado, assim, então e durante a sua prática o que você tem trabalhado assim, desenvolvido na sua experiência?

Entrevistada: Olha é mais uma coisa assim muito individual, né, eu nem digo se é coletiva, com grupo, e muito [...] de gestão assim da faculdade, eu acho que é uma coisa bem [...], então, eu acredito assim que a sua maneira, o seu modo de agir ele acaba sendo modelo para os alunos, isso é muito perigoso, né, porque pode ser um modelo positivo, com modelo negativo, então essa responsabilidade que o professor tem de ao mesmo tempo de estar ensinando, de estar ali como modelo para [...], eu cuido muito disso, então, assim, eu trato os alunos como [...], os assuntos assim formais eu trato de maneira formal, então eu acho que [...], mas tenho uma conversa por WhatsApp ali no corredor com aluno ele vai ter a liberdade de fazer o mesmo, né, então eu sempre trabalho com respeito, querendo respeito de volta, eu sempre trabalho de uma maneira formal, com e-mail, ao máximo tem que ser organizada ao máximo para o aluno ter segurança de como vai ser desenvolvida aquela disciplina comigo, então isso causa uma certa segurança e tranquilidade para o aluno, então eu acho que evita contratempo, que o aluno pode ficar com raiva por essas mudanças inesperadas, e vem com o tom agressivo para a gente, né, então eu tento ao máximo fazer um planejamento bem fechadinho, e respeitar ele ao longo do semestre todinho, então eu percebo que isso gera muita tranquilidade, segurança no aluno, nos passos que você vai dar, na disciplina, e isso evita muito contratempo, transtorno, e o aluno acaba às vezes não trabalhando um pouco assim a raiva dele, aquele sentimento negativo que ele está tendo, e ele acaba colocando isso de uma maneira [...] na condução do dia mesmo, sabe, não tenho uma coisa assim, ah, vou fazer isso para evitar, então eu acho que a sua maneira, a sua organização enquanto [...] que vai fazendo com que isso fique atenuado ou que [...] todo o semestre.

Entrevistadora: Sei. E durante essa prática você... na sua prática pedagógica você acha que existem fatores ali no momento da sua didática, do seu processo de ministrar a sua aula que possa levar o aluno a cometer a violência, você acha que existe um gargalo ali, uma forma dele fazer isso?

Entrevistada: Eu acho que o gargalo ele está ali se você for muito... Eu acho que é muito isso, sabe, uma violência, uma agressão leva a outra, então se você for muito rígida, se você for muito assim é daquele jeito e pronto, acabou, se você não aceita assim uma crítica construtiva, eles são muito assim, eles são muito para frente, então eles trazem, você pode passar um mês planejando a aula, vai ter uma novidade ali, então nem tudo que você fala para ele é o por último, então se você [...] receber outras referências de aluno, outros questionamentos, e si, né, eu acho que isso daí vai criando aquela panela de pressão, aquela construção de tensão ali na relação que vai culminar numa violência, então eu acho que isso é o gargalo, eu acho que se você for muito rígido, muito inacessível, muito Deus assim na sua aula, sabe, o que você disse é certo e pronto e acabou, se você não tem essa abertura para aprender, para refazer, para reformular ali, então [...], e isso é o que desencadeia essas agressões, né.

Entrevistadora: Então Leonora, a gente já está caminhando para o finalzinho da nossa entrevista, mas, assim, nós temos só duas perguntas, uma delas você inclusive já foi respondendo assim, a sua fala já vai contemplando a próxima pergunta. Mas, assim, o que mais te...

Entrevistada: Não sei se você está me ouvindo, para mim deu uma travada aqui agora.

Entrevistadora: É, deu, eu repito, nós já estamos finalizando, temos só duas perguntas, e aí eu queria perguntar para você, o que mais te chamou atenção, te impressionou de violência contra professor? Não precisa necessariamente ser na sua prática, pode ser com os seus pares, algo, ou com você mesmo, que você fala isso foi impressionante, isso me chocou.

Entrevistada: Angela.

Entrevistadora: Oi, você está me ouvindo?

Entrevistada: Para mim deu uma travada, se você puder refazer, agora sim, se você puder refazer a pergunta, que deu uma travada, ficou picando a fala.

Entrevistadora: Tá bom, eu refaço. Durante a sua prática pedagógica ou com os seus pares o que mais te impressionou contra a violência contra professor? Pode ser na sua experiência ou pode ser alguma coisa que você ouviu dos seus pares, tem algum fato?

Entrevistada: Eu acho que assim, o que causa mais espanto infelizmente é quando chega a vias de fato, digamos assim, né, há uma agressão física, quebrar algum bem seu, carro, quadro, computador, enfim, eu acho que isso é uma coisa que precisa... que chama mais atenção, e assim, e isso eu não tenho visto [...] não presenciei isso, nem comigo e nem com pessoas próximas, mas o que eu vejo é esse... o que é mais perceptível é esse adoecimento do professor para dar aula, eu nem sei dizer se é uma Síndrome de Burnout, mas é aquele assim, aquela descrença, aquele desânimo quando, quando você imagina que tem que dar aula para uma turma que é muito agressiva, que tem alunos muito agressivo, impedem de dar prazer em trabalhar, entendeu, você já começou com aquela turma, nossa, chegou a quinta-feira que é o dia da aula, isso é muito recorrente nas nossas reuniões, de ficar assim, [...] e buscando novidade, bibliografia recente, e coisas interessantes para dar para os alunos, a gente gasta muito tempo pensando e como se proteger legalmente, o mínimo que a gente

pode ofertar para evitar complicações com os alunos, desrespeito, então, assim ao invés da gente buscar prática, o semestre passado mesmo, ficamos muito tempo buscando práticas inovadoras no período, e a gente viu que a gente gastou tempo à toa, né, então [...] tem os slides passando, menos tarefa possível, então eu percebo a gente dedicando mais tempo para um respaldo legal do que mais tempo em tudo, em inovações pedagógicas, metodológicas, então nisso eu [...] isso desgasta, eu acho que é uma sinergia, sabe, isso é muito recorrente, isso é diário, toda a reunião tem isso [Risos].

Entrevistadora: E você visualiza alguma estratégia para esse enfrentamento contra a violência? Conseguiu me ouvir professora?

Entrevistada: Estou ouvindo.

Entrevistadora: Você consegue visualizar alguma estratégia para esse enfrentamento contra a violência?

Entrevistada: As estratégias contra violência elas funcionam, mas são muito lentas, são a passinho de formiga, então eu acho que a gente tem que começar a plantar essa sementinha, a dar esses primeiros passinhos de formiga, e eu vou dar um exemplo assim bem legal, por exemplo, a violência contra a mulher ela é muito forte ainda, mas já está na mídia, não é, uma coisa que a gente trabalha com mais facilidade, então isso vem assim rompendo ao longo do tempo, vem melhorando devagarinho ao longo, nessa questão da violência institucional, pedagógica, isso nem começou a ser discutido, né...

Entrevistadora: Perfeito.

Entrevistada: Das agressões, pode ser de aluno, professor, ou o do professor, quem está no cargo de gestão ele nem percebe ainda, é muito pouco discutido, então eu acho que tem que começar... talvez na semana de planejamento pedagógico, que a gente tem as reuniões com os professores, e levar isso para os alunos [...] estudantis que tem, então eu acho que ainda falta essa percepção de que todos ali estão suscetíveis a ser agressores, e que é um ambiente que a gente passa quase que a maior parte da nossa vida, né, assim, os professores, os alunos também naquele período da faculdade, então eu acho que é um momento que precisa ser discutido para a gente começar a ter, aí depois que teve que a gente começa a mudar, mas por enquanto eu não vejo ainda nada sendo feito nesse sentido, eu acho que começaria por ali, a tratar isso de uma forma de gestão onde os professores estão reunidos, onde os alunos estão reunidos, para que isso seja mudado nem que lentamente.

Entrevistadora: Entendi. Consegue me ouvir ainda professora, deu uma travadinha aqui.

Entrevistada: Consigo.

Entrevistadora: Sim. Então, agora finalizamos, mas eu sempre deixo uma pergunta assim em aberto com mais tranquilidade, que não está assim dentro daquele script que a gente faz da entrevista. Mas, assim, tem algo que você acha que ficou faltando, que pudesse ser pontuado nessa entrevista, gostaria de complementar?

Entrevistada: Eu acho assim, eu não sei se a sua pesquisa ela é só na parte de graduação educacional assim ou se ela envolve pesquisas, ela envolve as outras atividades de extensão, nesse ínterim, mas o que eu percebo hoje também que eu acho que poderia ser abordado por questões de orientação, né, essa relação de orientando/orientador nas pesquisas, nos trabalhos de mestrado, doutorado, PIBIC, eu acho que também acontece bastante para os dois lados, né, para os dois lados, nas atividades de extensão também, então eu acho que em toda a relação tem chance disso acontecer e vem acontecendo, então saiu um pouco do cenário de sala de aula, ir para dentro do laboratório dos núcleos de pesquisa, ir para dentro assim, ir para questão das extensão também, e eu acho que também à parte administrativa gerencial, eu percebo assim que tem muitos professores sobrecarregados com demandas de cobranças que vem da parte gerencial, administrativa da relação de afazeres, então não nem digo só de parte de coordenações ou outras não, mas eu digo assim, até na cobrança de ter publicação, de ter publicação em revista boa [Risos], isso daí acaba de certa forma a pessoa, é uma chantagem, é uma violência psicológica que está todo o tempo ali, você tem que produzir, você tem que ser bom, você tem que ser bom no ensino, na pesquisa, na extensão, tem que ter número, tem que ter quantidade, tem que ter qualidade, ela é muito silenciosa essa violência, então, assim, se você não publica, se você não tem xis alunos, se você fica assim nossa, mas fulano não faz nada, sabe, é uma... você fica ali entre os colegas que querem essa cobrança silenciosa, os seus superiores ali, entre os números que você tem que mandar no fim de ano, nos relatórios, então eu acho que isso também é muito forte na carreira profissional, então talvez assim, o que eu acrescentaria seria sair um pouco da sala de aula, o que eu digo que onde a gente tem o menor trabalho atualmente, e ir para esses outros campos que é o que assim, entope a vida da gente, desgasta a nossa saúde emocional, e é a nossa maior dedicação, então é buscar pesquisa de fomento, é buscar dinheiro, é buscar verba, é ter aluno, e ter o plano de trabalho que tem que entregar, saiu o edital de bolsa, saiu o edital de pesquisa, e isso daí é um fator que eu digo, né, pro minha experiência que acaba com o nosso psicológico, com o nosso emocional, e vai assim destruindo a nossa saúde aos pouquinhos.

Entrevistadora: Certíssimo [Risos]. Professora, eu quero agradecer imensamente, dizer que provavelmente vocês vão ver essa devolutiva dessa pesquisa a qual eu tenho me dedicado, já estou no terceiro período, então muito obrigada por você ter encontrado um horário na sua agenda para esses trinta minutos comigo, muitas contribuições importantes, então eu te agradeço muito, muito, muito, sucesso aí para você ao longo da carreira, e que essa bebezinha linda aí, tá bem, obrigada.

Entrevistada: Obrigada, sucesso também no seu doutorado e qualquer coisa eu estou à disposição.

Entrevistadora: Obrigada, Leonora, tchau viu, tudo de bom.

Entrevistada: Tá, até mais.

00:31:08

PARTICIPANTE 10

Tempo de gravação: 1:07:39

Entrevistadora: Assinar os termos.

Entrevistada: Sim, iniciais ALLS, hoje é dia 06 de abril de 2021, são dez horas e quinze minutos, e eu estou permitindo que essa entrevista remota seja gravada.

Entrevistadora: Obrigada, professora. Então, a gente começa aqui agora, já vou começar com aquelas perguntas, como é uma abordagem qualitativa eu início com algumas perguntas do percurso profissiográfico que eu vou precisar para completar, e aí nós vamos nessa conversa eu conduzindo aqui a entrevista, né. Professora quantos anos você tem?

Entrevistada: Sessenta, feitos na última sexta-feira.

Entrevistadora: Parabéns professora, receba os meus cumprimentos. E qual que é o seu estado civil?

Entrevistada: Solteira.

Entrevistadora: E qual a sua maior titularidade?

Entrevistada: Doutorado.

Entrevistadora: Quantos anos você é professora?

Entrevistada: Eu iniciei em 01 de novembro de 1989 na Universidade Federal de Goiás, e eu havia tido uma experiência anterior de um ano na Universidade Católica de Goiás na época, então são desde de 1988.

Entrevistadora: Então, professora... 88, né?

Entrevistada: Hum, hum.

Entrevistadora: Professora agora eu começo já com essa conversa partindo do que você, como que você conceitua violência no sentido amplo da palavra violência?

Entrevistada: Bom, sempre fazer conceituação não é uma coisa fácil, então vou trocar o seu pedido de fazer um conceito para te passar a minha percepção da Colab, né. Eu entendo violência como qualquer ato seja ele manifestado em palavras, em atitudes, em ações para com outras pessoas, para com outros seres vivos, seja pessoas ou então, ou mesmo animais em que o outro possa ser agredido, então você pode ser violento com a pessoa com palavras, com atitudes, com ações, você pode agredir fisicamente, você pode agredir emocionalmente, e violência para mim é sinônimo de agressão.

Entrevistadora: E você considera ou então nessa percepção como você coloca que existem gatilhos que podem desencadear a violência?

Entrevistada: Para que a pessoa seja violenta?

Entrevistadora: Isso.

Entrevistada: Olha, eu acho essa pergunta perigosa, né [Risos], porque eu lembro de ter participado há muitos anos atrás de um grupo lá na USP que estudava violência, violência urbana, e eu me esqueci do nome do professor agora que era o líder de grupo, mas eu lembro muito das conversas com ele que era um filósofo de como às vezes a gente justifica atos violentos, tá, a partir de o que você usou de gatilho agora ele usava outra expressão, mas é você ficar justificando atos violentos, ah, não, a pessoa agrediu para tomar do outro porque ela estava com fome ou ela agrediu porque ela não estava num estado psíquico de normalidade, então, assim, quando a gente procura definir esses gatilhos eu acho perigoso para que, porque eles podem-se transformar em justificativas para o agressor, né, eu acho que eu não sei, eu teria muito receio de te responder isso diretamente, por isso que eu estou aqui tangenciando, porque se eu disser que qualquer pessoa pode ser uma agressora, ou qualquer pessoa pode ser violenta na dependência de gatilhos. Então a solução para a violência passa por você anular os gatilhos, parece simples, né, parece uma linha reta isso, e eu sei que não é, tá. Eu acho que para a pessoa que ela é violenta, tá, que ela tem motivos, então existem psicopatas que não tem emoções, né, que eles não lidam com emoções, não é que eles têm problemas com emoções, eles simplesmente não lidam com emoções nenhuma, né, tem outras questões de pessoas que não chegam a ser psicopatas, não é questão de psicopatia, mas questão de caráter, de não experimentarem altruísmo, de serem pessoas exatamente egoístas e que justificam serem violentas com o outro desde que elas estejam bem. Agora eu penso que fome, estar... questão de uso de psicoativos, a pessoa não estar no seu estado normal ou alcoolizada, e por isso justificar, eu não creio que isso, seria no caso, por exemplo, de marido e mulher em que o marido agride a mulher, seja fisicamente ou não, porque o feijão ficou mal cozido, então não cozinhar bem seria o estressor, e passa a ser uma justificativa para o agressor, né, ela também faz por onde, certo. Então, assim, eu acho que eu meio que eu recusei responder à sua pergunta me justificando [Risos].

Entrevistadora: Muito bom. Professora, e durante a sua prática pedagógica você já sofreu violência?

Entrevistada: Já, eu sofri violência nos meus primeiros anos de exercício como professora, eu entrei na Universidade Federal como professora auxiliar, né, eu não tinha, aliás, eu tinha duas especializações, mas eu não tinha mestrado, doutorado, nada disso, aliás, quase ninguém tinha naquela época, né, mas as outras professoras tinham muita experiência, tinham professoras que estavam lá há bastante tempo, e eu cheguei como a professora mais nova do grupo, tinha muito tempo que não havia contratação, e eu tinha um grupo bastante consolidado e chegou, e eu era cria da casa também, ainda tinha esse outro fator, e eu me senti agredida vários momentos, especificamente por uma colega que tinha sido a minha professora, e essa colega ela gritava comigo no meio do corredor, ela dizia que eu estava pensando que eu era alguém, ela dizia que eu era esquisita [Risos], que eu era arrogante, mal sabia ela que eu de arrogância nunca tive nada, sempre tive muito de introspecção até os dias de hoje, que é interpretado de formas erradas pelas pessoas, de forma errada pelas pessoas, de acharem que a minha introspecção é arrogância, e não é, eu só estou quieta, calada, mas isso não significa que eu estou julgando ninguém, eu estou só observando o mundo, e foi um período muito difícil, muito difícil mesmo, porque eu não contava com... assim, agressão era uma pessoa que fazia, mas as demais não me protegiam, tá, então

acabava que eu não tinha refúgio, né, eu ia para o trabalho muito triste, eu me sentia muito excluída, tá, então além da agressão verbal tinha atitudes de não acolhimento que me agrediam muito, e com o passar do tempo eu fui aprendendo forma de me proteger disso, claro, mas, assim, nos últimos anos eu tenho até brincado com isso que eu já poderia estar aposentada há quatro anos atrás, cinco anos atrás, e eu optei por não aposentar, né, eu vou me aposentar, tenho a data, mas eu não... isso é segredo por enquanto [Risos], e eu comecei a perceber uma certa que eu chamaria de violência assim, sabe, e eu até como... conversei disso em casa dizendo que eu ia fechar a minha carreira profissional mais ou menos como eu comecei, sozinha, excluída e ouvindo coisas que eu preferia não ouvir, né, de ser uma pessoa arrogante, de uma pessoa distante, e achando isso muito ruim porque são pessoas que ficam julgando o outro sem conhecer o outro, no caso o outro sou eu [Risos], então, assim, interessante que as suas perguntas mexem com a gente, né, eu estou aqui tentando responder sem envolver a emoção, e acaba está sendo meio difícil, mas eu acho que você já passou por isso em outras entrevistas que você fez, né. Então, assim, agora já planejando o eu pouso como profissional docente, eu tenho maturidade, eu tenho ferramentas para lidar com essas violências, né, na vida acadêmica, e eu tenho procurado amenizá-las certo, até usando, e eu vou usar uma expressão bíblica aqui, que é o escudo da fé, eu acho que tem muito da palavra de Deus que nos ensina sobre comportamento, sobre como lidar com as outras pessoas, e uma coisa, escudo a gente usa na frente do coração, né, os guerreiros nas guerras para proteger o órgão vital, então é bem claro isso quando Paulo escreveu lá na epistola dele, que a gente deveria usar o capacete da salvação, o escudo da fé, e é isso que eu tenho procurado fazer nos últimos tempos em relação aos atos de violência, as atitudes de violência que às vezes eu percebo, e às vezes que não é comigo propriamente, sabe, mas é com algum colega, com alguém próximo de mim e que tomo as dores [Risos], sabe, às vezes dá gana assim de ir, de sair na defesa, até porque eu me sinto responsável, né, como decana na unidade, eu me sinto sim responsável por quem está chegando, para que isso não tenha, quem está chegando não tem necessidade de sofrer, né, pode muito bem ser acolhido e ter uma belíssima de uma carreira sem precisar de ficar gastando energia com esse tipo de defesa.

Entrevistadora: Professora, nessa nossa conversa aqui, uma entrevista, né, que ela é para ser leve, né, realmente assim uma troca, e aí eu fiquei aqui refletindo sobre as suas palavras aí do escudo, né, e pensando, nesse momento quando eu perguntei professora você já sofreu algum tipo de violência na sua prática pedagógica, você reportou a outras vertentes ou a outros cenário da sua prática profissional, ou seja, de um colega, não é, porque eu entendi correto, foi o colega que fez, né, e aí você responde a minha próxima pergunta já, entende, porque... E de aluno, de aluno teve algum aluno?

Entrevistada: Não, de aluno eu não me recordo, eu estou tentando puxar aqui, porque quando você fez a outra pergunta eu pensei assim aluno, colega, e eu já fui direto para colega porquê de aluno eu não me recordo, talvez até porque eu sempre, sempre meio arrogante, né, mas, assim, no início eu tinha, quando o aluno falava professora eu tremia, por que professora, outro dia eu era aluna, como é que agora eu sou professora, mas com o tempo eu fui adquirindo segurança e estudando, e tive o meu mestrado, então eu fui adquirindo ferramentas para lidar com o aluno, né, e se tem uma

coisa que eu gosto muito é de sala de aula, sabe, na graduação, na pós-graduação, e eu não tive filhos, e eu penso assim que tem muito das atitudes de alunos que são parecidas com atitudes de filhos, porque eu não tive filhos, mas eu sou filha, né, então [Risos], eu acho que tem coisas que você não precisa experimentar para saber como que é.

Entrevistadora: Com certeza.

Entrevistada: Então às vezes eu tomava alguma coisa de aluno, não, como agressão, mas como desobediência, como aluno que testa o professor, né, e com o tempo você vai adquirindo traquejo nisso, certo, hoje eu entro na sala de aula, tanto graduação como pós-graduação com muita tranquilidade, e no início mesmo que não fosse com tranquilidade eu tenho uma pessoa na minha vida que é a minha orientadora de vida, que é a minha mãe, e a minha mãe é enfermeira, hoje ela está com oitenta e sete anos, e uma pessoa extremamente inteligente, e eu sempre conversei muito com ela, então quando eu entrei na carreira docente e na federal eu falava com ela e contava não é mãe, quando me chama de professora eu tremo nas bases, ela filha, toda pessoa insegura passa a ser arrogante, então cuidado, cuidado com a sua insegurança, é melhor você assumir perante os seus alunos que você está insegura, que você não tem necessidade de saber tudo, você está aprendendo junto com eles, do que você tentar esconder a sua insegurança sendo arrogante com eles, então eu lembro que a primeira turma que eu peguei os alunos me chamavam de tia [Risos], ah, já que você, eu dizia, não é gente, vocês me chamam de professora eu fico pensando será que sou mesmo, então a gente vai te chamar de tia [Risos], enfim, eu não me recordo nenhum episódio, nem do passado remoto mais próximo, nem imediato eu tenha tido qualquer episódio de violência ou de agressão por parte de aluno, se teve eu devo ter perdoado e esquecido, porque incorporei como parte da minha formação docente.

Entrevistadora: Certo, muito bom. Professora você tem, assim no seu entendimento durante a formação dos profissionais da área da saúde essa temática violência contra professor você consegue enxergar ou ela está contemplada nos conteúdos mínimos dos projetos pedagógicos?

Entrevistada: Não, primeiro que eu penso que nós os professores... eu vou falar uma coisa que talvez não seja mais a realidade hoje, mas era algum tempo atrás, talvez uns dez anos atrás isso era bem real, nós não temos formação pedagógica para sermos professores, por exemplo, uma faculdade enfermagem, nutrição, medicina, a pessoa forma hoje e amanhã ela é professora, tá, sem nenhum preparo para isso, nenhum, nem didático, nem pedagógico, eu acho que não sabe nem a diferença entre pedagogia e didática, eu não sabia, certo, a gente fica replicando modelos, então, assim, ah, eu gostava de tal professor, então eu vou ser igual a ele com os meus alunos, nós temos tese, tem a tese da Iara Barreto, a tese não, a dissertação da Iara Barreto, que ela fez na faculdade de educação da federal de Goiás, isso ali na década de 1990 por ali. Recentemente teve uma aluna nossa Carla Lockemann que fez isso com professores tanto da federal quanto da PUC, professores de nutrição para essa questão da formação docente, discutindo essa questão de formação docente, mas mesmo esses poucos que lidam, né, hoje a federal tem, já tem alguns anos, né, aquele curso, quem entra como professor tem um curso didático docente, ou docente didático, algum coisa assim, que durante o período probatório o professor faz que é muito interessante, eu

tenho visto que os professores gostam de fazer, os que estão chegando, e que acabam instrumentalizando esses professores, mas pelo pouco que eu conheço mesmo desse curso, tá, não existe nada de abordagem sobre violência contra o docente, tanto é que assim, eu penso que o seu tema ele é mais do que relevante, ele é relevante, ele é útil, e ele é necessário, tá, porque quando a gente começa a falar sobre as coisas e que a gente começa a se instrumentalizar para resolver os problemas que essas coisas provocam.

Entrevistadora: E na sua opinião professora Ana, o eu você considera violência contra professor?

Entrevistada: Partindo de qualquer lado?

Entrevistadora: Sim.

Entrevistada: Bom, violência contra o professor uma das formas de você, e aí eu vou partir primeiro dos pais, tá, ou dos líderes ou dos chefes, então chefe de departamento, né, enfim.

Entrevistadora: Sim.

Entrevistada: Eu já ocupei alguns cargos na universidade federal, eu não ocupei cargo de reitora, mas eu já ocupei alguns dos outros cargos, né, já participei, às vezes as pessoas falam assim, aí comissão disso, já participei, isso é coisa de gente velha, né, que já fez muita coisa [Risos].

Entrevistadora: É experiência, não é professora [Risos].

Entrevistada: É. Então, assim, o que eu considero como agressão de chefes, por exemplo, é a falta de reconhecimento, essa é a primeira, para mim é a primeira grande agressão, sabe, de você vai isolando a pessoa, isolando, isolando, aquela pessoa ela não é lembrada para nada, ela não é solicitada para nada, certo, ela não é convidada para nada, certo, ela se faz presente por atos institucionais, então uma reunião de conselho que ela é conselheira, então ela tem que estar presente, né, mas mesmo em reuniões ela pode ter a palavra dela cassada, ela pode tentar falar e ser interrompida, isso é agressão, não é, isso não é só por ser mulher, eu estou falando, pesando como docente, tá, ou quando é falada é como se, sabe, você me falasse algo aqui agora e eu simplesmente desconsiderasse e continuasse falando, falando, e vice acabou de falar aquilo que é exatamente a resposta para o problema, mas foi você que falou, então é desconsiderado, então, eu considero, desconsideração, isolamento, desrespeito, não legitimação, sabe, porque existem espaço que são legítimos, né, e isso eu estou falando da posição de decana, mas tentando lembrar também de qualquer posição, por exemplo, posição de professor que está chegando, então ele não pode ser desconsiderado pela inexperiência dele, às vezes ele é inexperiente como docente, mas eles está vindo com uma bagagem, com uma experiência, uma experiência de vida, não é, às vezes uma participação dele como auxiliar de pesquisa, mas foi auxiliar de pesquisa do bambam, do creme de lacreme, e seria extremamente importante ouvi-lo, ele é violentado porque ele tem medo de falar, porque toda vez que ele pede a palavra não é dada, ou ele fala e ninguém ouve, todo mundo fica conversando, então isso é uma forma de ser violento. Então eu acho que entre pares

não existe essa coisa, e hoje com a tecnologia, né, isso dá gente está conversando aqui, por exemplo, tem as redes sociais, eu vejo muita violência, muita forma de você agredir as pessoas em rede social, sabe, você não pode manifestar o que você pensa porque senão alguém do seu grupo, né, da universidade, onde você trabalha fala nossa olha a cor daquela ali, né, ela é isso, ela é aquilo, ela pensa assim, assado, cozido, frito, e ela tem o direito de pensar como quiser, então as pessoas estão muito violentas em termos de rede social, por não aceitarem a contra-argumentação, não aceitarem conversar, porque conversa é quando eu escuto e eu falo, não só quando eu falo, não é, quando eu falo é monólogo, não é conversa, isso que a gente está fazendo aqui agora é uma conversa, você está me perguntando, mas você está me ouvindo, então eu vejo manifestações de violência explicita mesmo, sabe, em rede sociais, sejam elas quais forem, grupos de WhatsApp, grupo de WhatsApp então é uma coisa terrível, sabe, terrível, sabe, de ver colegas destratando o outro, ao ponto de dizer se... e tenho dito, estou saindo desse grupo [Risos], pô, mas espera aí [Risos], falou o que quis agora fica para ouvir o que não quer, não é assim, bom, então não fala bobagem, não é fica quietinho. Só um parênteses, outro dia eu sai de um grupo, mas eu sai foi só por cansaço de grupo mesmo, sabe, e sai tranquila, consciência tranquila, nem avisei que estava saindo, simplesmente sai e pronto, e aí uma colega foi no particular me perguntar o que tinha acontecido [Risos], eu falei não e porquê assim, eu participo de três grupos que eles mandam exatamente a mesma mensagem para os três grupos, então eu achei que eu não precisava ler a mesma coisa três vezes, só por isso, mas você não podia, e falei posso, posso sim, eu tenho autonomia, né, eu estou pensando até de sair de mais outros [Risos]. Então tem muito disso, sabe, eu percebi isso esse dia que eu sai desse grupo e logo, nossa, mas eu falei não gente, não aconteceu nada, estou eu aqui, oh, tá, eu só estou cansada de grupo, só isso. Agora violência da parte dos alunos, eu tenho visto, olha que eu não sei se é, porque eu acho que não é o seu tema, mas eu vou falar assim mesmo e como é uma entrevista que você vai, está gravando a vai editar se for o caso.

Entrevistadora: Sim.

Entrevistada: Eu já vi na minha carreira profissional agressão de professora contra aluno, tá, e aí houve revide, agressão verbal, agressão de comportamento, certo, e aí houve revide, revide brabo, tá revide assim de a coisa ir para a justiça, dos pais, dos alunos se envolverem porque era necessário, tá, mas quando, e na época eu ocupava uma posição que exigia que eu tomasse uma atitude, quando eu fui procurar saber o que tinha acontecido, não é, ouvir as partes, enfim, eu identifiquei que o que os alunos estavam fazendo era uma reação, não era uma ação tinha acontecido por parte do professor, então as violências, os atos violentos que eu já vi por parte de alunos até onde eu me recordo foram reacionários, certo, eles não foram atos gratuitos, ou direcionados, tá, e aluno é aluno, não é gente, aluno é aluno, então eu acho que nós professores nós temos que nos envolver com eles, nós temos que direcioná-los, uma das últimas turmas da graduação que eu estava dando aula presencial uma aluna um dia me parou na porta da sala e... professora, eu queria convidá-la para você fazer parte, um aluno que participa muito de sociedade organizadas, ONGs, eu queria convidá-la para participar conosco de um projeto de pesquisa que nós vamos fazer e tal, e tal, e eu olha eu fico honrada, não é, tinha sindicato envolvidos, coisas de âmbito nacional, eu fico muito honrada, mas o que é mesmo que vocês querem estudar, e aí quando ela foi

discorrendo ela queria estudar uma coisa que ela queria que a resposta fosse uma, sabe, se a resposta fosse diferente daquela, e porque a pesquisa estava errada, tá [Risos], coisa de criança, né, e daí eu falei olha, o que vocês querem estudar é fantástico, eu não tenho bagagem para isso, porque depende de estudos qualitativos, o pessoal da área de política pública deve ser muito bom nisso, eu te indico algumas pessoas, mas eu já te falo, que vocês têm que ir para pesquisa nem sempre a gente tem um resultado na pesquisa, o resultado que está buscando, né, e pode ser que você vai encontrar um resultado que vai ser um resultado que vai legitimar uma coisa que você está atacando hoje, uma coisa que você está dizendo hoje que não presta, que é ruim, que não é assim, que devia ser do outro jeito, aí você vai faz uma pesquisa com todo o método, com todo o rigor do método e chega numa resposta que, isso que estão fazendo é o melhor que podia ser feito, e aí? Né. Então, assim, eu topo participar, mas com esse compromisso, e se o resultado for esse nós vamos publicar no Jornal Nacional da Globo, tá, aí nunca mais ela falou no assunto [Risos], então, assim, eu não sei se ela estava me testando, né, se ela estava procurando um jeito de dizer para mim que as coisas que eu estava falando em sala que ela não concordava, tá, mas eu não senti como agressão de forma nenhuma, eu acho que depende muito disso, é o nosso comportamento docente com relação aos alunos nós estamos ali para trabalhar com a formação dos alunos, você está agora no doutorado, você está se formando como cientista, você já é mestre, então você se não tem, não conheço a sua trajetória, né, mas se não tem a experiência com aluno ou se tem já sabe disso, né, que é nossa responsabilidade, não e responsabilidade do aluno, essa comunicação docente e aluno, eu falo demais, não é Angela desculpa.

Entrevistadora: Não, não muito... olha eu estou aqui me deliciando, está muito bom professora, porque eu fiquei aqui, né, você falando e eu fiz a pergunta dizendo da temática se está contemplado nos conteúdos, e falei sobre essas... na sua opinião sobre o que você considera violência contra professor, você me trouxe o cenário acadêmico e trouxe o cenário dos alunos, né, não é assim propriamente na sua prática. E aí eu pergunto, e você tem desenvolvida professora alguma assim estratégia, experiência que vise o respeito a toda comunidade acadêmica durante a sua prática pedagógica?

Entrevistada: Com os alunos eu nunca pensei nisso, com os alunos a minha preocupação sempre foi de ser a melhor professora possível, então de nunca ir para aula sem me preparar, sem fazer um acordo com eles no primeiro dia, olha, eu vim trazer o que eu tenho para vocês é isso, e o que eu espero de vocês é isso também, né, e eu não... Para ser bem direta em relação a minha prática com os alunos eu nunca pensei nisso. Com relação a outro tipo de violência que é entre os meus pares e o que eu já comentei, né, eu tenho desenvolvido alguma coisa, mas é muito pessoal, eu acho que eu nunca parei para pensar que isso era assim intencional, sabe, eu acho que eu fui desenvolvendo formas de proteção, tá, uma das formas que eu fui... uma das coisas que eu percebo é que eu fui ficando, eu que já não era, eu gosto de falar, você está percebendo, mas eu gosto de falar quando eu percebo que eu estou sendo ouvida, tá, e aquela, e que numa condição de respeito, né, que mesmo que possa estar dizendo algo contrário ao pensamento da outra pessoa, a pessoa está ouvindo, que ela vai dizer que eu vou argumentar, que eu posso inclusive ser convencida do contrário, e se for esse o objeto da discussão, tá. Mas quando não eu fui desenvolvendo algumas coisas sim, como por exemplo, ficar mais calada, tá, então em reuniões, em reuniões

de conselho, mesmo agora nessa vida que a tecnologia que nos aproxima, eu entro na reunião, eu escuto, tá, eu escuto todo o mundo, eu presto atenção, eu tomo nota, mas eu fico calada, tá, agora não cita o meu nome, porque se citar o meu nome vai ser um pretexto para eu pedir a palavra [Risos], essa foi uma estratégia que eu desenvolvi, então, assim, se o meu nome ele é citado a seja para o bem ou para o mal, né, aí eu peço a palavra porque o meu nome foi citado, eu vejo isso como uma estratégia para mostrar, né, e tem acontecido em vários cenários Angela, de ao usar essa estratégia eu depois receber mensagens individuais dizendo, assim de professores do grupo mais jovem dizendo assim, professora a senhora precisa falar mais, porque toda a vez que a senhora fala a senhora nos protege, interessante que eu estou te respondendo isso aqui e estou pensando, essa é uma estratégia, e eu realmente eu preciso, não que eu precise sair falando, não é igual louca, mas eu preciso me posicionar mais, não por mim, porque eu já estou, né, não é arrogância, mas, assim, não tem como eu ser atingida, não é, eu desenvolvi formas para isso, mas eu preciso de me posicionar mais para defender os outros de violências que eu vejo acontecer, tá, então uma estratégia foi essa de me calar e que você está me fazendo repensar, a outra estratégia foi de me isolar mais do que ficar calada essa, né, porque eu fui me distanciando de todos, tá, e passando a ter um convívio com os colegas meramente profissional e necessário, então acabou que por conta do que eu contei logo no início da minha carreira e tudo, o que eu respondia quando gritavam comigo no corredor, que eu era esquisita, que era estranha, que era isso, que eu era isso, que eu era aquilo, eu respondia dizendo, aqui eu preciso colegas, amigos eu tenho lá fora, tá, isso acabava era provocando mais discórdia [Risos], e eu acabei fazendo amigos em outras unidades acadêmicas fora da minha unidade, porque na minha unidade era tudo muito espinhoso, sabe, muito espinhoso, e eu acabei conduzindo toda a minha carreira dessa forma, buscando ser acolhida em outros locais, já que onde eu estava não era acolhida eu ficava de fora, tá, então eu fui , eu vinha de uma prática multiprofissional, eu trabalhava na secretaria estadual de saúde, e eu trabalhava num setor que era multiprofissional, enfermeira e os médicos nutricionistas, fisioterapeutas, todo o mundo no mesmo lugar, na mesma sala, discutindo tratamentos, políticas públicas, e eu entrei como professora achando que dentro da academia também era assim, e não era, tá, então eu fui... uma das, além de me calar eu fui me distanciar, e eu me distanciei procurando lugares onde eu podia ser acolhida, um lugar foi a faculdade de medicina, outro lugar foi o hospital das clinicas, aliás, o hospital das clínicas me acolhei tanto que tinha gente que pensava que eu era enfermeira do HC, né, eu era mais chamada para projetos do HC do que dentro da minha unidade, tá, a faculdade de nutrição também me acolhei muito, a faculdade de educação que foi onde eu acabei fazendo o meu mestrado, né, também fui muito acolhida na faculdade de educação, e aí eu fui criando essas estratégias, ao ponto de hoje o meu núcleo de pesquisa, o meu núcleo de trabalho fisicamente ele fica fora da minha unidade de trabalho, eu sou vinculada a uma unidade acadêmica, tem, né, na gestora atual tenho por ela o maior apreço, o maior respeito e tudo, ela me dá muito apoio, né, mas também é uma pessoa que me conheceu pouco, porque o tempo que ela estava na unidade eu não estava, eu estava ou no doutorado ou depois eu fui trabalhar na administração, e ela me conheceu de ouvir falar, tá, então quando ela iniciou o trabalho dela eu já era essa pessoa calada e distante, e foi assim que ela me viu [Risos], e isso, essas foram as ferramentas que eu usei, tá, nunca precisei desenvolver nenhuma com relação aos alunos, mas com relação aos meus pares eu tive que desenvolver ferramentas de proteção.

Entrevistadora: Ok. E professora, se nós ampliarmos essa violência contra o professor para no cenário assim maior, não sei se maior talvez eu não coloquei bem, mas, assim, um cenário amplo, o que mais te impressionou de violência contra professor, não precisa ser na sua unidade, não, assim, né, vamos fazer, a palavra não sei nem se cabe holística, mas, assim, na totalidade teve algo que você falou nossa essa violência contra o professor me chocou ou me chamou a atenção?

Entrevistada: Eu vou falar de forma bem generalista, tá, você estava formulando a pergunta eu estava aqui pensando, eu acho que o que mais me impressiona na violência contra o professor é quando o professor é calado, sabe, quando eu digo para o professor ou eu calo ele numa rede social ou mando ele calar a boca, certo, ou ele está falando e outra pessoa toma a palavra dele, toma mesmo, sabe, você não ouvir o que um professor tem para dizer eu considero uma violência também, né, mas é uma agressão menor, agora você nem deixar ele falar então isso de forma bem ampla, sabe, de nós não sermos ouvidos a nível municipal, estadual, federal, em tempos de pandemia, por exemplo, a gente tem tanto para oferecer, né, a gente tem tanto para... não é com arrogância que eu digo isso não, mas, assim, gente olha a força de trabalho que nós somos, né, e ode a gente vai a gente vai com o nosso bando, a gente vai com os alunos junto, e aluno vai e vai feliz, sabe, e aluno trabalha, eu fui trabalhar outro dia na campanha de vacinação, e é a coisa mais bonita de ver, sabe, o aluno ali ele não estava ali por agradecimento de ninguém, e aí uma aluna chegou para mim e disse assim professora será que eu posso sair até tal hora, porque eu não queria chegar de noite em casa, quer dizer, ela já podia ter ido embora há mais de horas [Risos], sabe, ela estava ali como voluntária, entendeu?

Entrevistadora: Entendo.

Entrevistada: E eu falei, oh, minha linda, né, a gente não vai ficar aqui até seis horas da tarde, se você quiser ir agora já pode ir, aí ela falou não, não, eu não vou agora, ainda tem, olha o tamanho da fila, eu não vou agora, então, assim... e nesse local lá mesmo em uma das vantagens, né, de ser decana e que ou é aluno ou é filho de aluno, ou é mãe de aluno, né.

Entrevistadora: Com certeza.

Entrevistada: E eu cheguei e me apresentei para a equipe que estava trabalhando lá, para equipe da secretaria, e eu não sei, alguém devia me conhecer, que na hora que eu falei meu nome eles falavam assim, ah, professora [Risos], e estava o pessoal do Ministério Público lá, sabe, e eu vi que o pessoal da secretaria estava muito tenso, tinha um outro grupo também que eu não sei bem de onde era, estava muito tenso, eu fiquei pensando aquele dia, sabe, assim, o quanto, a gente podia estar com mais uns dez, vinte postos de vacinação na cidade, bastava o que, bastava que a academia estivesse sendo ouvido, né, e quando eu estou falando em academia eu estou falando de nós professores, certo, então eu acho que uma das grandes violências, e uma violência atual é essa, e essa de... essa violência de nos emudecer, de colocar uma... não é uma máscara para proteção não, é uma máscara nos emudecer, sabe, de não nos deixar falar, nós que estamos precisando de gritar muitas coisas, e aí passa por violência contra a mulher, que nós temos muitas mulheres na carreira docente, né, de uma mulher estar falando, como é mulher aí o outro que é homem te faz calar, nem

percebem, às vezes fazem isso sem perceber, e é um colega seu, sabe, então eu acho que essa é uma grande violência hoje, Angela, tentando pensar agora para responder [Risos].

Entrevistadora: Então, e dentre essas... é assim, toda essa sua colocação tão rica que muito vai contribuir aqui com a minha pesquisa, você ver estratégias de enfrentamento a violência dos alunos contra o professor?

Entrevistada: Eu não entendi, dos alunos?

Entrevistadora: É, por exemplo, existem estratégias, você consegue visualizar estratégias que nós poderíamos trabalhar para minimizar, a palavra seria assim, de enfrentamento.

Entrevistada: Você está falando assim, e violência dos alunos contra o professor?

Entrevistadora: Contra o professor.

Entrevistada: Angela, eu consigo ver, primeiro porque eu não vejo violência dos alunos igual eu te disse, para mim a violência que por ventura aconteça com um ou outro professor, e deve acontecer, mas comigo não aconteceu, ela é reativa, tá, então se ela é reativa no meu ponto de vista a estratégia que eu poderia ver e que os professores fossem mais professores e menos autoridades, entendeu, que é aquela história do professor entrar na sala de aula como soberano e rei, eu acho que já foi, né, já deu, então o professor tem que entrar como professor, e se ele entra como professor com comportamento de professor ela vai entender quando um aluno for desrespeitoso, por exemplo, né, e não tomar aquilo como agressão, mas ele pode tomar aquilo como um desafio do aluno para com ele, certo, e fazer o enfrentamento daquilo não com autoridade, certo, mas fazer o enfretamento daquilo com diálogo, fazer o enfrentamento daquilo tentando entender. Eu vou contar um fato bem rápido, uns anos atrás eu comecei a ter uma dificuldade dentro de sala de aula porque o mundo mudou muito, e essa questão de gênero é uma coisa que eu tenho muita dificuldade de lidar, né, eu tenho dificuldade de lidar, não passa por questão de aceitação, não é isso, tá, e porque eu não sei como agir, eu fico toda atrapalhada, tá, antes para mim era fácil, né, home m e mulher, homossexual, gay e pronto, tá, mas agora não é, o mundo não é assim, né, tem os trans e tal, e eu estava na sala de aula um dia, e aí durante toda a aula, que eram duas aulas seguidas, teve uma aluna que ficou com a cabeça abaixada, dormiu a minha aula inteira, tá, bom, eu podia tomar aquilo por uma agressão do aluno está dizendo assim, oh, não estou nem aí para a sua aula, tá, ou podia tentar descobrir o que estava acontecendo, bom, não está nem aí para a minha aula isso não me fere mais, tá, o acordo que eu tenho com os alunos é o seguinte, você precisa estar presente nem que seja de corpo, tá, é a sua obrigação estar aqui, afinal de contas eu devo ter alguma coisa importante para fazer, e estou aqui, e porque isso aqui deve ser importante também, então fica quieta aí, tá, se quiser dormir pode dormir, mas não pode me atrapalhar. Aí eu esperei a aula terminar, aí quando a pessoa estava aprontando para sair eu falei olha, você espera que eu quero conversar com você, quando eu falei isso os colegas que estavam do lado ouviram e fizeram um cerco ao redor, ficou a pessoa, ficou mais duas de cá e duas de cá, eu falei não eu quero falar só com essa pessoa, aí os colegas responderam não para falar com ele vai ter que ser junto com a gente, tá, eu já achei aquilo, né, falei o que está acontecendo aqui, aí eu fui lá e fechei

a porta, né, falei tá bom, a minha pergunta é bem simples, eu quero saber, oh, criatura por que você dormiu a minha aula inteira? Porque tá bom você pode até recuperar depois estudando e tudo, mas puxa vida a minha aula estava ruim desse tanto, eu estudei para dedéu para vir para sala, não estava bom, então eu queria saber, aí ele levantou com os olhos assim muito sono, falou não professora e porque eu estou vindo do plantão, tá, falei tá, e como é que você está fazendo no estágio? Porque se você for para o estágio depois de um plantão acabada do jeito que está indo, né, você vai errar demais, sabe, isso é muito sério, ah, professora, mas eu preciso trabalhar, porque... aí começou a contar a história de vida, tá, era uma situação de uma jovem trans que estava iniciando a transição, sabe, fez toda a transição durante o curso, formou inclusive, iniciou menina e terminou rapaz, e eu não sabia lidar com isso, tá, aliás eu nem sabia o que estava acontecendo, né, nem sabia que existia isso, e aí foi ela que me contou o que era e tal, as colegas que estavam juntas contaram, né, oh, professora na casa dela o pai está batendo, tal, tal, tal, e aí a gente foi conversar para ver a possibilidade de conseguir uma bolsa de trabalho, a universidade estava no tempo das vacas gordas, para conseguir uma bolsa de trabalho para ela poder sair daquele trabalho que ela estava fazendo noturno, uma colega já ofereceu que tinha um quarto vago que ela podia ir morar com ela, né, enfim, começou haver uma movimentação, na próxima aula ela já chegou, ela já assistiu a aula inteira, tá, as colegas também ficaram bem comigo, tá, mas o dia que eu perguntei para ela, porque já estava de barba e tudo, e quando eu ia fazer a chamada ainda não tinha a coisa do nome social, quando eu ia fazer a chamada quando chegava no nome dela eu pulava ou via que ela estava na sala, citava, botava frequência e pulava, né.

Entrevistadora: Muito bom [Risos].

Entrevistada: Aí um dia eu peguei e perguntei, sabe, eu falei, chamei lá bem distante, né, falei escuta, como é que eu te chamo? Eu preciso da sua ajuda, porque está escrito lá um nome, só que quando eu olho para você eu não vejo esse nome, aí chorou na minha frente e disse para mim que eu era a única professora que tinha tido essa preocupação, tá, eu falei tá, mas eu continuo sem saber como é que eu te chamo [Risos], e aí falou qual que era o nome social que... e foi interessante que naquela semana o conselho superior aprovou o nome social dentro da universidade, e já trocou os documentos e tudo, então quando você fala em termos de estratégia para evitar agressão de lá para cá, sabe, eu acho que, de novo, eu acho que a estratégia é a mesma, e que a gente precisa de aprender a ouvir mais, eu estou contando uma história aqui não para me engrandecer, de forma nenhuma, que não tem nada de grandioso nisso, eu estou dizendo que eu não sei lidar com algumas situações, tá, e exatamente por não saber lidar eu acho que uma ferramenta que todos nós podemos ter que ela não custa muito, tá, é ouvir, saber ouvir as pessoas, saber ouvir os alunos, às vezes ele tá agressivo, mas ele tem um motivo para aquilo, o motivo às vezes não está nem dentro da sala de aula, é isso.

Entrevistadora: Professora, essa é a minha última pergunta agora, e eu deixo sempre aberto porque, assim, enquanto pesquisadora, né, aí dessa formação do doutoramento, quando eu elaboro a minha entrevista às vezes a gente nunca consegue contemplar tudo, você acha que, se você gostaria de discutir, falar alguma coisa que você pensasse que na temática poderia estar sendo contemplada e que eu não a fiz?

Entrevistada: E olha eu não sei se é bem um acréscimo, uma lacuna, não sei, eu vou dizer e você decidi, tá [Risos]. Eu acho que você contemplou a questão da violência docente que é o tema do seu estudo, e com relação à violência entre... que o professor sofre de outros docentes, acho que foi esse uns dos caminhos, com relação aos alunos, certo, mas não ficou muito claro, eu tentei responder isso lá na entrevista, agora eu estou lembrando de algumas questões. Não ficou para mim, eu acho que é uma coisa que precisa de carregar um pouquinho mais nas tintas, a violência contra o professor em termos institucionais, sabe, assim, porque dentro das universidades, eu posso falar das universidades públicas, eu sou cria de uma e trabalho em uma, não sei se é diferente universidades particulares ou confessionais, comunitárias, mas a questão da hierarquia, sabe, eu tentei abordar isso um pouco em uma das suas perguntas aqui, porque eu acho que tem muito disso no nosso meio, sabe, de pessoas que ao assumirem, e às vezes assim a coordenação de uma comissão, acha que aquele ali colocou ele num degrau, que ele pode olhar assim de cima para baixo, e ele já começa a tratar o outro diferente, entendeu?

Entrevistadora: Entendo.

Entrevistada: E esse tratar o outro diferente com agressões mesmo de não chamar o outro para participar, de não dar voz ao outro, sabe, de quando o outro... Eu vou dar um exemplo tosco aqui, mas, por exemplo, eu tenho que falar de mim, né, por isso que você está entrevistando, não tem que ficar falando nos outros, eu sou professora há tanto tempo, e eu sou pesquisadora, eu tenho diretório de pesquisa no CNPq, sou líder de grupo, e trabalho, o meu grupo de pesquisa o grupo onde eu trabalho, não sou dona dele não, é o grupo onde eu trabalho é na liga de hipertensão arterial, e nós temos muitas, muitas, muitas pesquisas em desenvolvimento, tem pesquisa começando, pesquisa terminando, pesquisa no meio, né, é uma ebulição isso, de 2019 para cá houve muita mudança dentro da liga para melhor, inclusive, já era bom ficou mais bom [Risos], e aí há uns tempos atrás eu só estou dando o exemplo para fortalecer isso que eu estou acrescentando tá, se é que eu estou acrescentando alguma coisa, eu conversei lá no meu grupo, que a gente estava recebendo um projeto de fora, um financiamento pequenininho, mas, assim, com a possibilidade de alunos nossos, e universidade italiana, de aluno de lá vindo para cá, daqui indo para lá, né, e inclusive eu já estava engatando uma aluna minha de doutorado para ir fazer um pós-doc lá e tal, e quando o projeto chegou eu conversei no nosso grupo, falei olha, todos os projetos, e a parte administrativa quem cuida sou eu, né, falei olha todos os projetos eu tenho passado pela faculdade de medicina por conta do vínculo, né, do vínculo da liga ainda hoje, tá, mas eu queria passar esse projeto especificamente pela faculdade de enfermagem, tá, porque vai dar a força, enfim, por conta disso que eu acabei de dizer, aí ninguém se opôs de forma nenhuma, claro Ana manda bala, né, e eu fiz do jeito que eu fazia, que eu faço na outra unidade acadêmica, né, mandei o protocolo e tal, tal, tal, veio tanto questionamento, mas tanto questionamento em cima do protocolo, a gente não podia mudar o protocolo, era um protocolo que vinha da universidade italiana para cá, a gente podia fazer uma ou outra sugestão, mas nós éramos convidados, você não entra na casa do outro ditando normas, né, aí eu peguei aquilo e falei gente, mas se a gente for fazer todas essas alterações aqui o projeto é outro, não é, questionando coisas assim. Bom, enfim, resumindo, eu voltei para o meu grupo, né, falei assim, esquece o que eu falei, não vai dar certo, vamos pelo caminho que a gente sempre fez, tá, porque

aqui a gente não vai conseguir andar com isso, né, e a gente tem presa senão a gente vai perder a oportunidade, o projeto está andando agora, e não pode passar por lá, e por que, tá, eu sinto que toda a vez que alguma coisa entra com o meu nome ela é revisada com maior rigor, tá, seja numa reunião, seja num... é agressão que eu ainda sinto, certo, só que quando isso só me diz respeito para mim eu só olho e sigo em frente, mas quando isso envolve, por exemplo, envolvia a unidade, era ganho para a unidade, sabe, aquilo me machucou, me doeu, e isso foi recente, aquilo assim, eu fiquei, eu demorei muito tempo para descer, sabe. Então é esse lado aí da violência que eu não sei de que forma, não sei nem se cabe no seu estudo, mas e que muitas vezes a agressão não é do meu par, e quando esse meu par acha que tem poder, tá, e aí ele pensa assim, ah, você está se achando, então espera aí que eu vou te pegar, e aí o que ele está querendo pegar não está nem pensando nisso, está só querendo trabalhar, é, não tem tempo ruim não, tá, vai cuidar da sua vida que dá mais resultado, né, e tem muito disso, às vezes é um presidente de comissão, há alguém porque passou a ser representante não sei aonde, aí se empodera, e porque está empoderado acha que o outro é menor, e começa a ter atitudes grosseiras com o colega, sabe, é de grossura mesmo, e grosseria para mim é agressão. Eu acho que eu falei muito [Risos].

Entrevistadora: Professora Ana Luisa, olha eu ficaria aqui muito mais tempo com você porque as suas contribuições são fantásticas assim, a cada momento eu consegui ir categorizando, sabe, na sua fala, eu consegui categorizar, e isso para um pesquisador da área qualitativa é maravilhoso, né, a gente fica em ecstasy, eu sei que falar para você, você sabe imensamente interpretar o que eu estou dizendo aqui. Então eu te agradeço do fundo do meu coração, te dizer assim que eu aprendi uma palavra muito importante, eu sou docente também, que esse escudo eu estou precisando trabalhar viu professora, um escudo na frente do coração [Risos], muito boa fala.

Entrevistada: Protege o seu coração.

Entrevistadora: Fenomenal, eu espero estar devolvendo isso depois, né, assim todo o meu produto, acredito que vá contribuir para toda academia sim, e olha muito obrigada.

Entrevistada: Eu fico feliz de poder contribuir, e se você precisar de qualquer outra coisa que eu puder ser útil você sabe que eu sou acessível.

Entrevistadora: Sei sim, um grande beijo professora, muito obrigada, viu.

Entrevistada: Um beijo, tchau, tchau.

1:07:39

PARTICIPANTE 11

Tempo de gravação: 01:11:29

Entrevistadora: Pode ser assim?

Entrevistado: Pode. Ângela, eu falo muito e sou prolixo, você pode me cortar.

Entrevistadora: [Risos] Pois é, essa é uma das razões maravilhosas que eu quero muito a sua entrevista, porque eu sei que muito contribuirá com o meu trabalho, tenho certeza absoluta, professor. Vamos lá então. Eu quero que você...

Entrevistado: Desculpa. MAN, tenho quarenta anos, eu permito a utilização das minhas falas para o projeto de doutorado da Ângela Gilda Alves e vamos trabalhar.

Entrevistadora: Muito bom. Professor, nesse primeiro momento naquele perfil psicográfico eu fiz algumas perguntas que são, assim, já dentro da pesquisa também, qual que é a sua idade em anos, professor?

Entrevistado: Eu tenho quarenta anos.

Entrevistadora: Você é brasileiro?

Entrevistado: Brasileiro, natural de Minas Gerais, tenho... Sexo masculino...

Entrevistadora: Sim, casado?

Entrevistado: Casado.

Entrevistadora: Estado civil, né, aquela história, para ficar menos informal, aquelas perguntinhas, fica mais tranquilo. Qual é a sua maior titularidade?

Entrevistado: Doutorado.

Entrevistadora: Doutorado, tudo bem, certinho. Professor, quanto tempo que você dá aula?

Entrevistado: Eu iniciei em dois mil e dois para o nível médio, porque na época permitia, né, que o graduando ele ministrasse aula para o ensino médio, era na época do PROPAAE, então acho que foi em dois mil e dois, em dois mil e dezesseis eu fui professor substituto e logo depois, em dois mil e oito eu passei no concurso para professor efetivo.

Entrevistadora: Certo, então, agora nós iniciamos, né? A minha temática é a violência contra o professor no ensino superior em saúde, é uma temática inicial, não posso dizer que seja o título, mas já tem alinhavada essas questões, Marcos, para você qual é o conceito de violência?

Entrevistado: Ângela, acho que o conceito de violência ele é muito amplo e ele passa vários aspectos que eu acho que ele vai desde a violência física, violência emocional, violência de gênero, a falta de respeito, a questão da hierarquia, eu acho que violência é aquilo que você expõe a vítima e geralmente ocorre entre duas pessoas que um é oprimido e o outro opressor, e eu acho que como nós vivemos numa sociedade na qual a violência ela até então, que eu acho que a gente tem pouco estudo, até por ser algo considerado normal eu acho que ela é um conceito ainda muito complexo.

Entrevistadora: Eu estou com um sonzinho aqui vou aguardar uns minutos para ver se sai, que você sabe que essa coisa de home office você tem que intervir aqui com esse barulho da propaganda do cliente, então só um minutinho que eu já faço a segunda pergunta, está ok? Fechei a janela ali, fui ali frechar a janela para ver se minimizava um

pouquinho, mas não adiantou muito. Bom, então esse é o seu conceito de violência nessa amplitude, e agora, assim, você acredita, Marcos, que existem fatores que desencadeiam, que são desencadeantes para a violência?

Entrevistado: Sim, acho que [...] acho que é a questão da formação do indivíduo, mas quando eu falo formação é formação pessoal mesmo, né, acho que desde as experiências que o indivíduo tem na infância, na adolescência, eu acredito muito que como é um ditado popular que tudo vem de Deus, eu acredito muito nisso, então eu acho que o indivíduo cometer violência eu acho que vem muito das experiências dele e também perpassa pela falta de conhecimento, muitas vezes as pessoas cometem alguns atos de violência por desconhecimento mesmo de que aquilo é violência, principalmente que a gente está numa sociedade que cada vez mais a pressão, cada vez mais a competitividade, cada vez mais a vaidade são aspectos que eu acho que aflora esse lado mais agressivo do ser humano, eu acho que todos nós temos momentos que a gente tem maior ira, mas tem pessoas que conseguem trabalhar, exteriorizar esses sentimentos, já a maioria, por questões sociais, econômicas, de educação mesmo elas não tem essa habilidade de controlar ou de buscar algum tratamento, alguma terapêutica, e acaba que isso pode se tornar algo que seja normal no cotidiano dele, e também acho que falta muito a divulgação do conhecimento, acho que é responsabilidade nossa de educadores, de pessoas que temos conhecimento, a gente mais, nós estamos com muita informação, mas pouco conhecimento, né, e isso principalmente na nossa sociedade que a gente fala que trabalha com Paulo Freire, metodologias ativas, mas eu acho que a gente está muito distante, a gente aponta os erros, dá informação, olha, a violência existe, ela tem esse e esse conceito, mas acho que ela não transforma em conhecimento, e se ela não transforma em conhecimento não vai se conscientizar sobre aquelas temáticas, principalmente quando fala de violência que é algo que na nossa sociedade a gente não pode falar sobre isso, é como se fosse um tabu, ah, violência a gente não pode falar porque a opressão ela é muito grande, porque violência é você estar cometendo um crime muito sério, não entende os vários tipos de violência.

Entrevistadora: Você já sofreu algum tipo de violência durante a sua prática pedagógica?

Entrevistado: Várias, Ângela, primeiro que existe dentro da minha profissão, na minha percepção existe uma violência enorme em relação ao gênero, é como se todo homem heterossexual não pudesse ser enfermeiro, como é uma profissão que ela partiu das mulheres nem como as mulheres cada vez mais elas estão ponderadas e isso é ótimo, a gente quer isso mesmo, mas eu acho que as mulheres elas têm cometido muita violência e aí de tidas as formas mesmo, com o homem hetero, dentro da nossa profissão, eu sinto que eu incomodo muito as pessoas, que eu sofro violência todo tempo, violência quando questionam minha sexualidade, como se eu, hetero, não pudesse ser enfermeiro, eu sou homossexual ou deveria ser mulher. Outra questão de violência é que as pessoas acreditam que isso é inerente ao genótipo, o homem ele tem uma certa forma de falar, uma certa forma de lidar com as questões totalmente diferente da mulher, então da mesma forma que a gente fala que a mulher tem TPM, a mulher ela tem tripla jornada e aí ameniza um pouco a violência que a mulher comete dentro da nossa profissão, e o homem não, o homem tem que ser super homem, ele não pode mostrar as suas fragilidades, existe também a questão da masculinidade, o homem da

enfermagem ele tem que o tempo inteiro mostrar que ele é gentil, mostrar que ele não é preconceituoso, mostrar que ele tem que estar análogo a conversa das colegas, mesmo se for uma temática que ele não concorda, então um exemplo muito simples, as colegas estão conversando sobre Tupperware, que é uma coisa que a gente vê muito, é algo que naquele momento eu não tenho interesse, então, assim, se eu não interessas naquele momento é como se eu não fizesse parte do grupo, já se eu for conversar sobre algo que faz parte do meu mundo eu não tenho essa troca, então é muito difícil, na minha percepção, ser hetero na enfermagem, me envolver com outros colegas é muito difícil mesmo, e atualmente eu acho que a violência maior, Ângela, que eu tenho sofrido é que é como se a vitória do homem, nós somos uma sociedade machista e aí é lógico, a gente não pode negar, a mulher, eu admiro demais a mulher independente da profissão, eu tenho compaixão porque eu acho que é muito desigual mesmo a questão da visão da mulher na nossa sociedade, a violência que a mulher sofre é muito grande, mas a mulher ela eu acho que tem que entender que, primeiro, o homem ele foi ensinado, ele foi adestrado, na verdade, desde criança a ser o que não transmite sentimento, a ser o indivíduo que talvez fala de uma forma grossa, de uma forma ríspida e que isso é natural, então algumas vezes eui falo algo que para mim é muito normal, mas para a mulher pode ser que ela ache que isso é uma violência, e eu acho que é preciso você informar, Marcos, eu acho que isso da forma que você está falando você está mais ríspido, que se eu for numa partida de futebol nós vamos, a gente xinga a mulher, a sogra, e aquilo ali a gente trava como violência eu para nós, aquilo faz parte da nossa história social, assim como para você isso é direito de fala, eu acho que isso é muito importante, as mulheres entenderem que é lugar de fala, desculpa, lugar de fala, não tem como a mulher falar sobre ser homem na enfermagem sendo que ela não tem lugar de fala, demais, nossa, eu sofro demais por ser hetero, é como se os alunos achassem que eu não sou merecedor, e agora, infelizmente, eu acho que tem aumentado muito hoje porque a gente tem percebido que o homem na enfermagem, ele tem, por questão da força, por questão da objetividade, ene fatores, eu percebi que o homem ele tem tido, não concordo, mas eu tenho visto que ele tem sido privilegiado em questão de trabalho, as empresas, principalmente as empresas particulares elas estão preferindo mais os homens por ene fatores, então acho isso, as mulheres veem isso como uma ameaça e aí eu acho que acaba cometendo vários tipos de violência.

Entrevistadora: Professor, então, assim, ouvindo aí a sua fala, quando você fala que já sofreu em vários tipos você classificaria essas que você elencou como psicológica, emocional ou sexual, verbal?

Entrevistado: Assim, eu acho, assim, a psicológica é no sentido, com certeza isso é muito frequente, o tempo todo eu tenho que demonstrar que eu faço parte do grupo, como eu sou minoria então o tempo todo eu tenho que fazer parte do grupo, e para eu fazer parte do grupo eu tenho que... como adolescente, eu tenho que tentar me inserir no grupo de alguma forma. Outro tipo de violência que eu já sofro demais é a questão das próprias alunas, o movimento feminista está muito presente na enfermagem, então o homem, eu, mas vários colegas já relataram, eles estão na enfermagem, tudo é, ah, ele cometeu machismo, mas, assim, as mulheres elas têm que entender que o homem também sofre violência, a partir do momento que você fala, nossa, será que ele é gay ou não, então todas às vezes que eu entro em sala de aula é essa frase que eu digo, será que ele é gay? Se ele tem esposa ele não deve ser gay, então acho que isso é

uma violência muito grande, eu tenho, não como provar, isso não precisa, mas, assim, eu tenho que lidar com esse tipo de violência, com essa pressão. Outra pressão que eu acho é que, como é homem, como ele é enfermeiro e é homem ele tem que ter um carro bom, ele tem que impor mais com a equipe de saúde, as mulheres esperam, as alunas esperam que eu chegue para o médico quando acontece lá, um exemplo, o médico prescreveu, realizar cuidados de enfermagem, as alunas, as colegas acham que por eu ser homem hetero eu tenho que ir lá e brigar com o profissional médico, como se isso fosse inerente ao ser homem, eu não tenho essa obrigação por somente ser... eu vou lá, vou falar com educação, vou virar amigo da pessoa, outro tipo de violência é a questão da vestimenta, então, assim, é óbvio que se eu sou homem e eu tenho vontade de ir ao banheiro é óbvio que... e eu estou na sala de aula é óbvio que, não só, mas é óbvio que... como eu posso te dizer, Ângela, eu não sei se eu vou me expressar bem, mas pela anatomia do homem quando ele está com vontade de ir ao banheiro e fica segurando muito pode acontecer que ele tenha esse tipo de situação, então isso eu já sofro demais, as mulheres falam que eu estou sentido atração por elas, o tempo todo, Marcos, você é homem, não fique no seu gabinete só você e uma mulher, Marcos, não abrace as alunas, Marcos, você não pode chegar perto das alunas e ficar com as alunas, então eu fico o tempo inteiro com medo, eu vou te falar a verdade, eu fico o tempo todo com medo porque tudo é como se, tudo que eu falo, tudo que eu faço é como se eu estivesse cometendo algum abuso de todas as razões, infelizmente não falam em violência masculina, se você quiser ver, agora, na matéria de ginecologia, por exemplo, fala, violência obstétrica, mas e a violência com homem, eu sofro violência, as enfermeiras não queriam que eu assistisse o parto, mas o médico ele vai estar aqui, então o médico ele entendeu o meu sentimento, então o homem ele sofre violência, quantas vezes, Ângela, agora sim, estou falando de uma forma geral, mas quantas vezes eu tive que ter relação sexual com mulher sem a minha vontade, mas para provar que eu sou macho, que eu sou viril, isso quando eu era solteiro, ou atoe casado mesmo, ah, eu tenho que fazer senão a minha mulher acha que eu estou traindo, aconteceu um fato muito interessante na academia porque eu fui homenageado várias e várias vezes, então na nossa unidade nós somos três homens, um eu acho que é homossexual e eu acho que ele verbaliza isso, e para mim independe se é homossexual ou não, mas eu sou o mais jovem, os outros dois eles são já com cinquenta e poucos anos, então é como se eu representasse algo que incomodasse mesmo as alunas, principalmente as colegas de trabalho, nossa, mas ele é homem, por que as alunas preferem ele homem para ser o paraninfo e não eu, mas ele é tão bravo, tão rígido, eu acho que isso é uma violência muito grande, e está achando sempre, isso eu vejo muito com os homens, eu queria muito, Ângela, talvez seja até seu pósdoc, você trabalhasse com violência contra o homem, e aí é muito engraçado que eu trabalho com privados de liberdade, e lá no presídio tem três mulheres que eles foram presas porque elas cometeram violência contra os parceiros, e aí as pessoas, por exemplo, fala de Maria da Penha, mas não fala que Maria da Penha também é para o homem, então é uma violência muito grande, eu não posso chegar para alguém e falar, olha, eu fui vítima de violência, a mulher ela quis que eu tivesse relação sexual com ela, mas eu não estava à vontade, me obrigaram a beijar aquela mulher, mas eu não queria naquele momento, mas eu fui cobrado pelas colegas, e no trabalho é muito pesado, Ângela, muito pesado, porque, assim, se uma aluna me abraça significa que eu estou dando atenção para ela e posso estar pensando alguma outra coisa, e você sabe que na enfermagem as alunas dela são muito [...], então, assim, as alunas precisam demais de atenção, então como eu trabalho com

infecção sexualmente transmissível então várias, e sexualidade, ene alunas me procurar para falar, professor, a minha camisinha estourou, o que eu faço, professor, eu transei sem camisinha, por favor, passa o teste rápido para doenças sexualmente transmissíveis, infecções sexualmente transmissíveis, então isso incomoda muito, ele é homem, então por que as alunas o procuram? Por que elas o procuram, se eu sou mulher? Eu sou prolixo, pode me cortar, viu?

Entrevistadora: Não, é porque eu quero fazer um link que está bem próximo da minha próxima pergunta que é com toda essa sua fala eu fico pensando aqui, assim, qual é a sua... não é um pensamento Ângela, é de dentro da pesquisa. Quais são as suas atitudes, você já falou para mim de medo, eu fico com medo, literalmente eu fico com medo, nessas situações qual que é a sua reação?

Entrevistado: Na docência a reação que é o isolamento, eu me sinto isolado do grupo, eu não me sinto pertencente àquele grupo, e agora está muito na moda lugar de fala, na docência, como assim, não ter lugar de fala, tudo bem, a enfermagem é uma profissão majoritariamente feminina, mas eu tenho lugar de fala sim, eu sou enfermeiro, seu sie dar um banho, eu sou pai, eu sei cuidar de uma criança, ah, ele não tem jeito de dar banho numa criança, tenho sim, é lógico que eu tenho, não é porque eu sou homem que eu tenho que ser aquela pessoa rude, então eu acho que o isolamento é por esse fator, outra por questão de ajuda psicológica, que os homens na enfermagem eles buscam apoio psicológico porque a pressão acho que é muito grande, a pressão da sociedade também porque acho eu a sociedade pensa assim, nossa, mas ele é homem, enfermeiro e ganha tão pouco, talvez até a mulher dele ganha mais, e isso também é uma violência muito grande, mas por que eu posso ser enfermeiro e talvez minha esposa ganha mais do que eu, não tem problema nenhum, eu não vejo problema nenhum. Outra questão é a questão das parcerias, eu acho que as parcerias elas ficam mais difíceis, eu tenho dificuldade de fazer parcerias porque eu não tenho lugar de fala, está falando sobre enfermagem, sobre cuidado, então eu acho que não tenho mais lugar de fala, eu percebo que as colegas incomodam muito quando, por exemplo, eu recebo um edital, um fomento, eu percebo, eu acho que não é mania de perseguição porque é muito nítido, eu percebo que acho que incomoda um pouco, porque, nossa, eu cuido eu sou mulher, eu estou numa profissão que é mais nitidamente mulher, nossa precursora é uma mulher, a sociedade toda acha que é mulher, no nosso local de trabalho nós somos 85% mulheres, mas por que entre todos ele conseguiu esse fomento? Recuo de cargos de gestão porque é muito difícil um homem ser gestor de um grupo grande de mulheres, que as mulheres também elas já têm ideia preconcebida, aí o homem tem que estar o tempo inteiro provando, gente, eu não sou machista, gente, eu não sou homofóbico, gente, eu não sou, então tenho que estar o tempo todo, eu não sou, eu não sou, então retrai, eu não sei se você percebe, os homens, os héteros eles se calam, então é outra forma de enfrentamento e também acho que o principal eu diria que eu vejo é a angústia de saber que não existe estudo sobre violência sobre o que é ser homem na enfermagem, sabe, é muito triste, porque, assim, as mulheres tem que entender que é difícil ser hetero nessa profissão, e quando fala, e eu já pensei trabalhar sobre isso, fazer pesquisa, mas eu tenho certeza absoluta se eu fizer essa pesquisa aí eu vou ser mais rotulado ainda, então tem até [...] que eu tenho dificuldade, medo e angústia de falar sobre isso. Já sofri, eu sou um professor, não sou o melhor dos professores, lógico, mas várias, já fui várias vezes, assim, pela minha... eu estou desde

dois mil e oito, mas que eu ando afastado do [...] de saúde, mas eu acho que já fui homenageado umas quatro vezes, como paraninfo duas, vezes, então eu acho que isso é um termômetro, que eu não sou tão... então eu acho que... e aí eu sofri já dois processos por questão de ser machista, por questão de ser homofóbico, por questão de ser rígido, mal educado, mas não, é um erro muito simples, Ângela, eu sou o tipo de professor que finalizo estagio e eu não faço uma confraternização com bolo, com, sabe, eu não consigo ficar no final de um estudo de caso comendo bolo, sabe?

Entrevistadora: [Risos].

Entrevistado: Isso é uma violência muito grande, é como se eu não fosse humano, como se eu não fosse professor que tivesse humanização, é como se eu não participasse do processo de ensino e aprendizado e eu não colocasse o aluno como centro do ensino e aprendizado, quando eu chego no hospital eu fico preocupado muito com a postura das mulheres, então eu vejo muito isso principalmente para a professora Marialves, na minha época eu tive até aula de etiqueta com ela, eu acho que a mulher tem que... e o homem, tem que se portar, porque a gente já vem de um histórico tão difícil, então quando eu vou conversar com as alunas elas acham que eu estou sendo violento, não, eu só falo, meninas, vamos ficar mais caladas, vamos ficar mais centradas, meninas, eu não acho certo você sentar no colo da colega, mesmo que vocês estão só esperando o estágio começar, meninas, vamos parar de ficar vendendo revista da Avon, que aqui não é o momento para isso, quando eu falo isso soa como violência para elas, já quando uma mulher fala isso não é uma violência, sabe, então eu acho que ser professor, seu sei como docente para transmitir conhecimento, eu não estou para fazer amizade, a primeira coisa que eu falo para as alunas é eu estou aqui não para ser amigo de vocês, eu estou aqui para ser o orientador, se caso, no transcorrer da nossa história a gente for amigos ótimo, eu vou levar para a vida toda, isso assusta muito, sabem, então eu vejo as minhas colegas que... aí é minha percepção, tá, as alunas elas fazem o que for necessário, carrega no carro, leva para sua casa para fazer confraternização, pega os alunos no final de estágio e vão tomar um sorvete, então, assim, é uma relação que eu acho que não é uma relação de professor, eu acho que esse respeito existe, e eu também não aceito não me chamar de professor, eu não sou o Marcos, eu não sou o Marquito, eu não sou o jacaré, eu não sou igual muita gente chama, eu sou o professor Marcos, quando a gente estiver lá fora pode me chamar de outra forma, mas dentro do ambiente da escola eu sou o professor, é senhor, é o professor, então acho que isso elas veem como uma forma de violência, que estou sendo machista, que eu estou sendo opressor, acho que é mais nesse sentido.

Entrevistadora: Então, e aí Marcos, olha só, durante essa sua passagem, prestou processo seletivo, está inserido como professor algum tempo já dentro da UFG, eu fiquei aqui analisando e queria perguntar, essa temática, violência contra professor ela está contemplada nos conteúdos mínimos dos projetos pedagógicos lá da faculdade de enfermagem?

Entrevistado: De forma alguma, de forma algum, na enfermagem é como se o homem não existisse, tanto é que é as meninas, colegas, minhas pupilas, professor Amílcar, por exemplo, é claro que você não vai colocar nomes, tá, professor Amílcar, minhas pupilas, as meninas, então, assim, o homem não é inserido nesse projeto pedagógico os homens não são inseridos, para você ter uma ideia dentro dos projetos políticos pedagógicos

existe saúde da mulher, mas não existe saúde do homem, por quê? Em nenhum momento se discute saúde do homem, e aí quando eu falo saúde do homem também entra saúde do homem professor porque ele vai escolher a carreira, então de forma [...] muito a ente tem lei Maria da Penha, eu nunca vi nenhum projeto político pedagógico alguém que fale que a lei Maria da Penha vale para o homem também, e quando eu falo isso todo mundo fica assim, ele é machista mesmo, ele está falando sobre isso, então eu acho que a gente tem que evoluir mesmo, a profissão de enfermagem tem que saber que existem homens que ele é uma minoria e como minoria ele sofre violência de todas as naturezas. No nosso banheiro não tem espelho, por que no das mulheres tem espelho, tem sabonete, tem aqueles frascos e tal, no meu banheiro não tem espelho, e não é porque eu sou metrossexual, por que o espelho é básico, eu estou com uma lesão na pele eu tenho que ver, eu tenho que me olhar no espelho, então isso é um reflexo tanto para o homem que sofre violência no meio acadêmico, então enfermagem, assim, não incomoda mais porque a gente já acostumou, na minha época de formação de professor era muito difícil porque, meninas, vocês entenderam, colegas, então eu acho que isso é bem difícil, esses projetos pedagógicos não tem nada que abordem o homem, e aí como que nós, enfermeiros, falamos dentro do nosso projeto político pedagógico formamos indivíduos que devem cuidar do indivíduo como um todo sendo que no projeto pedagógico do curso de enfermagem tem saúde neonatal, saúde da criança, saúde do adolescente, saúde do idoso, saúde da mulher e não tem saúde do homem, isso mostra como a mulher é violenta como o homem, ela acha que o homem não merece entrar no círculo de vida para nós enfermeiros sabermos lidar com os homens.

Entrevistadora: E aí, professor Marcos, você tem desenvolvido experiências docentes, com toda essa sua fala, fala, assim, pontual, essa fragilidade das mulheres, né, em não reconhecer e cometer a violência, você tem desenvolvido ações para contemplar isso na comunidade acadêmica? No projeto pedagógico não tem, porque a maioria certamente são construídos por mulheres, homem é minoria, né, essa conversa que a gente está tendo então não contempla, muito bem colocado aqui por você, e aí na sua prática você desenvolve alguma ação? Assim, pontuando essa condição da saúde do homem, a lei Maria da Penha e tudo isso que você disse.

Entrevistado: Sim, por isso, primeiro eu ofereço um livro que chama saúde do homem, sexualidade humana, então esse é um livro procurado por toda... na última foram cento e vinte alunos de todas as áreas do conhecimento, então, e, assim, quando eu falo que o homem ele tem direito a assistir o parto da mulher, quase nenhum aluno sabe, quando falo o que é masculinidade, orientação sexual não sabem, quando eu falo de violência, que o homem sofre violência a maioria não sabe, quando eu falo que lá no presídio tem mulheres que foram presas por cometer violência contra os homens todo mundo acha um absurdo, então tem essa disciplina que eu criei justamente para isso, para tentar aos poucos inserir, e quando, eu sou professor de enfermagem clínica, quando estou no hospital para exemplificar as teorias de enfermagem eu sempre pago o homem como protagonista, então se estamos trabalhando com teoria (00:33:47) aí as alunas elas falam e eu sempre falo e o homem aí, onde que está o homem aí, então a gente faz essa reflexão, a gente trabalhando com (00:34:01) puder fazer com o mapa e o genograma aí sempre tem laços fortes, laços fracos, frágeis, aí eu sempre faço esse questionamento, mas por que, será que o homem ele está num relacionamento frágil, será que um determinado... passou muito isso, ah, mas o homem, parte do pressuposto

de que o homem ele não participa do parto, eu pergunto, meninas, as mulheres quando vai escolher alguém para assistir o parto elas pedem para os maridos para estarem, não, elas pedem que a mãe participa assistir o parto, eles pedem a melhor amiga ou a madrinha assiste o parto, ela esquece o homem, e o homem a gente vivencia a gravidez, então é óbvio que se eu não vivencio eu não vou saber lidar com aquilo. No pré-natal, existe o pré-natal masculino, existe o pré-nupcial masculino, existe política de saúde do homem, de 2009 ela traz tudo isso, mas isso não é abordado, para você ter uma ideia não existe nenhum projeto pedagógico que traz a política nacional de atenção a saúde do homem, sendo que o homem ele vive oito anos a menos do que a mulher, a cada cinco óbitos de indivíduos, quatro mulheres são... quatro são homens, no último censo existia quatro milhões de mulheres a mais do que homens, então, assim, criou-se como o homem ele é o super homem, e a mulher não, isso não é trazido na prática, no cuidado, então eu acho muito claro, eu falo com os alunos, mas será que cuida integralmente, por que, cadê o homem aqui... ah, mas homem não gosta de cuidar, homem nem tem que cuidar mesmo, será por que ele não vem, ou será por que ele foi convidado, ah, o homem não vai para atenção básica, por que você acha que não, porque se ele vai fazer procedimento estético talvez ele fosse chamado para atenção básica, mas como que nós vamos fazer isso, então é uma violência enorme contra o homem, eu acho, assim, eu brinco muito com os alunos, os alunos preparam as atividades de brincadeiras na atenção básica e eles fazem aquela brincadeira do balão e eu falo, meninas, vocês acham que um homem vai pegar um balão, colocar na barriga de outro homem e sentar no colo dele?

Entrevistadora: [Risos].

Entrevistado: É do imaginário do homem, ou então nas políticas pedagógicas ou nas reuniões pedagógicas, Ângela, para decidir algo fica duas horas discutindo, coisa que naquele momento não são necessárias, e o homem ele não tem esse perfil, isso é genética, é questão de genética que genotípica também, então homem não tem paciência de ficar lá discutindo algo e isso ocorre demais, não existe objetividade, os conselhos diretores tem quatro, cinco horas e chega no final não decidiu, nem, eu acho que falta, assim, a objetividade e talvez por isso o homem eu acho que ele é mais... sofre mais violência nesse sentido.

Entrevistadora: Entendi. Agora, assim, eu vou puxar aqui assim, durante a sua prática pedagógica por que você acha que o aluno usa de violência contra o professor. Vamos trazer para a prática, na sua aula, assim.

Entrevistado: Primeiro, Ângela, que dentro da enfermagem existe uma cultura de superproteção do aluno e existe uma cultura que a enfermagem ela quer tratar o aluno como se nós fôssemos mães, ainda existe muito figura materna, eu acho que desde o início do curso a gente, eu acho que a gente não prepara os nossos alunos para as dificuldades, na verdade a gente tem que empoderá-lo a questão que eles têm todos os direitos que eles têm que reclamar ali mesmo, mas eu acho que a gente não fala muito dos deveres, na enfermagem existe muito questão de ser enfermagem mesmo de a gente cuidar das pessoas, a gente, no projeto pedagógico eu vejo que a enfermagem a gente tem uma visão muito materna, sabe, e aí nessa visão materna eu tenho certeza absoluta de que a gente faz com que o aluno ele nem saiba respeitar muito essa diferença entre o professor e o aluno, não que o professor seja superior ao aluno, mas

há de ter um respeito com o professor, então, Ângela, é muito engraçado que dentro do hospital os próprios alunos quando é da medicina chamam de doutor, os nossos alunos da enfermagem não chamam o professor de doutor, e aí eu pergunto, como vocês querem nos valorizar sendo que vocês não me chamam de doutor, eu dou doutor, e lá no hospital quando a gente está na prática pedagógica os internos eles chamam de doutor, então não valorizam a nós. Outra coisa que eu sempre falo para os alunos, na minha prática pedagógica, alunos, tudo bem, nós sabemos que vocês têm esse e esse direitos, mas e os deveres, quais são os deveres que vocês têm para com o professor? Então é como se na enfermagem é porta aberta, sabe? O aluno ele tem qualquer coisa com o professor ele já está na direção dele ouvindo, isso não acontece com os outros cursos, eu fui professor da medicina, Ângela, infelizmente, como eu amo demais, é muito dolorido falar isso, mas os alunos da medicina me chamavam de doutor, os alunos da medicina em momento algum eles tinham desrespeito, eles sempre queriam aprender, nunca me interromperam, então você está na aula, aí levanta a mão, você está naquele gás ensinando, aí levanta a mão um aluno da enfermagem, professor, está na hora do intervalo, ou seja, você está no meio de ensino e aprendizagem e isso é muito importante, eles não respeitam isso, já um aluno de medicina, também já dei aula para farmácia, eles têm essa cultura, e aí, Ângela, eu não queria que você colocasse aí especificamente, mas pode falar, pode até colocar meu nome, eu já estou acostumado como professor doido de enfermagem [Risos]. A minha esposa ela é enfermeira, e ela é professora da área básica, então, Ângela, ela é enterologista, então todos os alunos passam por ela, e lá não existe essa questão na área básica de qualquer aluno ir lá na direção reclamar, não, tem que ter hora, tem que ter agenda, tem que ver qual que é a disponibilidade, conversa com o professor, na enfermagem não, na enfermagem o aluno bate na porta o coordenador de curso já atende o aluno, e ouve o aluno sem ouvir o professor, isso é a maior violência dentro da sala de aula, ou então a direção, estou ministrando aula, ao aluno vai lá, reclama, a direção vai lá na sala de aula me procurando, sem nem saber o que aconteceu.

Entrevistadora: Entendi. Então você estás me dizendo aí também que existe a violência contra o professor ao aluno, mas existe uma violência também de gestão, de pares, dos colegas, né, você citou vários outros grupos. O que te chamou a atenção, não precisa ser especificamente na sua pratica ou também se tiver acontecido, mas o que foi, assim, que durante, você olhou... assim, nossa, esse fato de violência me chamou a atenção, me impactou, você já presenciou, assim, isso foi impactante, dentro da temática violência.

Entrevistado: Uma certa vez eu fui fazer uma atividade fora da sala de aula e aí, Ângela, teve um momento que aí não tinha lugar para todo mundo sentar e tinha um banco de madeira longe, aí eu falei, na época era Pedro e Álvaro, Pedro e Álvaro, sejam cavalheiros, vão lá e peguem o banco para as meninas, eu fui, na verdade, linchado, que absurdo, você é machista, porque nós mulheres não podemos ir lá buscar esse banco, nós temos os mesmos direitos dos homens, nós podemos fazer tudo aquilo que os homens fazem, me acuou de uma forma que eu não soube lidar com aquilo, nem consegui ministrar a aula que, assim, foi uma violência tão grande porque, assim, eu simplesmente quis ser gentil, da mesma forma que tivesse uma mulher mais frágil eu ia pedir isso também. Outra coisa que chamou muito, assim, me marcou demais, eu fui chamado na direção, para eu não abraçar alunas, mas as alunas que vem até mim,

assim, eu tenho um bom relacionamento com os alunos e então, assim, tem muitos alunos que gostam de mim e que me entendem, como eles falam, professor, a gente tem você como exemplo de uma pessoa que nos ouve, e aí me falaram, você não pode abraçar, e eu sem falar nada, [...] você não pode abraçar, você não pode ficar sozinho com alunas no gabinete, e aí eu falei, eu não vou deixar de abraçar as alunas quando elas querem, porque um abraço, eu aprendi na academia que um abraço é terapêutico, quando eu abraço uma aluna não é a mesma coisa que quando eu abraço minha esposa, que para abraçar eu não preciso encostar os quartos, assim, eu vou encostar somente o meu tronco, então isso foi algo que também me chocou, assim, demais, e acho que outra maior violência foi quando eu sofri o processo, as alunas elas foram até a direção, eu não fui chamado, eu acho que no mínimo teriam que ter me ouvido, e aí quando foi na sindicância chamou uma colega enfermeira, Regiane, do hospital das clínicas, que era coordenadora, à época, do curso de graduação, aí primeiro quando eu ouvi foi um grupo de professores que eles fazem primeiro essa apuração, e depois vai para as outras instâncias, e aí na verdade eu fui punido não pela fala dos alunos, por ela, aí sim, as falas dela, posso até te encaminhar o depoimento dela, primeiro que ela não permitiu que eu ficasse na sala para ouvir o depoimento dela, isso foi minha ex-professora, inclusive, ela não permitiu, não queria que meu advogado ficasse na sala ouvindo o depoimento dela, mas era meu advogado, ele falou que era direito meu, ficou, e aí no final eu...foi para a frente o processo e não pela fala das alunas, pela fala dela, então, assim, foi algo, também, que foi uma violência extrema, extrema, extrema que eu nunca pensava, não tive apoio dos meus pares, não tive nenhum apoio dos meus pares, e sempre... e é diferente quando é com homem, Ângela, o homem faz alguma coisa toda a faculdade, nossa, olha o que ele fez, ele faz isso, aquilo, quando a mulher comete algum erro, que nós somos professores, a gente está cometendo erro o tempo todo, como eu te disse, algumas formas de violência emocional, até, talvez, psicológica, a gente faz sem perceber, por isso que eu acho que existe semana pedagógica, por isso que existe formação, por isso que existe estudos como o seu para a gente identificar e para a gente resolver e cortar essas arestas, mas não, e, assim, por exemplo, estágio em docência, ontem mesmo foi muito interessante, ontem a aluna falando que a disciplina de uma professora muito renomada, professora Ana Clara, a disciplina dela são só os alunos de mestrado e doutorado que estão ministrando, como estágio em docência, só que isso é ilegal, o aluno não pode fazer isso, mas não reclamam, mas se eu fizer isso, Ângela, e não é uma questão de perseguição, pode depois fazer outras entrevistas com os alunos, não é perseguição, se eu fizer isso eu mesmo vou ter que ficar lá na direção, então isso é algo também que marcou e outra coisa, assim, foi, assim, extremamente violento é questão de lugar de fala, quando eu ouço isso dentro da enfermagem eu fico em pânico porque nunca eu posso falar nada porque eu não tenho lugar de fala, que eu sou homem e aqui a maioria é mulher, então eu acho que isso com meus pares é o pior, mas você não pode falar que isso não é lugar de fala, mas se não é lugar de fala e eu faço parte de um grupo não tem como, gente, agora vamos ter só homens na enfermagem, eu vou dar aula só para os homens, e outra coisa que a maior violência que eu sofri foi a questão de sempre quando tem homem na sala fala que eu sou o puxa saco ou que eu estou dando mais atenção para os homens, não é isso, é porque, primeiro, são homens, eu me identifico mais, segundo, lá fora são as pessoas que eu tenho para conversar, então, assim, eu converso sempre com os alunos, independente até os heterossexuais, bissexuais, com porteiro, geralmente é home, que eu converso bastante, minhas alunas acham que eu sempre, os alunos homens vão

sempre passar porque eles são privilegiados, é outra violência também porque eu trato os meus alunos da mesma forma.

Entrevistadora: E você, você vê diante de toda a sua fala possíveis estratégias de enfrentamento à violência do aluno contra o professor?

Entrevistado: Eu acho que primeiro, principalmente na enfermagem eu acho que primeiro, na primeira aula eu já acho que deveria falar sobre os direitos e sobre os deveres, e aí dentro dos direitos e os deveres enaltecer a nossa profissão, eu acho que falta muito isso porque a partir do momento que o aluno sabe o que é ser enfermeiro, o aluno ele respeita o professor eu acho que ele vai ter mais sucesso, é no sentido de que eu vejo muito as outras profissões no início, no primeiro encontro eu falo, gente, vocês são bons, a enfermagem é boa, a enfermagem, homens e mulheres cuidam, não, já no primeiro momento já tem essa questão da violência, qualquer coisa que vocês precisarem vocês podem me procurar, a formalidade, sabe, Ângela, eu acho que a enfermagem precisa de formalidade, eu falo para todo mundo, você não precisa andar com uma Louis Viton, não precisa andar com uma roupa de marca, gente, hoje qualquer lugar tem uma roupa, então, assim, sempre busca estratégias para os alunos, ah, não, ele não tem condições, vamos deixar ele ir com esse sapato rasgado mesmo, não, mas a gente sabe que tem uma possibilidade, então, primeiro, acho que a questão de no primeiro dia de aula já falar para os alunos, enfermagem não é fácil, enfermagem é um curso difícil, um curso que merece ser respeitado, acho que a primeira coisa é isso. Segundo é que é a questão do posicionamento dos professores da enfermagem, eu acho que os professores da enfermagem eles têm que entender o que é o papel do professor, o professor ele orientador, não é pai e mãe, nós não conseguimos, Ângela, nem resolver os nossos problemas emocionais, nós não conseguimos resolver os nossos problemas familiares e a gente quer resolver os problemas do aluno, e a gente acaba sendo psicólogo do aluno, sendo terapeuta do aluno, isso não é nosso papel, a gente faz com que o aluno ele nos veja como salvador da pátria, e quando a gente não consegue atingir a expectativa do aluno [...] professores, a questão da informalidade, é um estudo de caso, não é uma festa, sabe, então tem que mostrar ao aluno o que é formalidade, então tem estudos de casos que o aluno chega para mim no quinto período, ou seja, a gente já fez metade do curso e eles não sabem essa forma didática de estudo de caso, e aí eu pergunto, mas como que foi, a gente fez uma rodinha e aí enquanto a gente discutia a gente comia, mas, Ângela, eu acho que isso não é [...] ativa, existe um momento para refeição e existe um momento para discussão científica, eu, marcos, não consigo comer um, bolo de chocolate que eu amo e prestar atenção numa fisiopatologia de uma doença, porque o bolo que eu comi naquele momento é mais importante e mais saboroso do que eu ver uma fisiopatologia, mas eu acho que tem que mostrar sim, sala de aula é algo importante, algo que o professor vai lá para transmitir o conhecimento e você tem que falar assim, eu sou professor, outra, a informalidade em relação a chamar professor por apelido, sabem, eu acho que você está fora, tudo bem, ô propizinho, ô jacaré, ô princesa não sei o que, sabe, isso é muito feio, eu não vejo isso como normal, não, o professor ele tem que manter a sua postura de professor. Outra questão eu acho que se nós sabemos o quanto o aluno... se o aluno sabe que nós o avaliamos por que a gente não pode saber quem foi o aluno que me avaliou? Para você ter ideia o nosso sistema de avaliação o aluno vai, de forma espontânea, então eu já tive vários e vários zeros em tópicos de pontualidade, assiduidade, entrega de plano de disciplina, agora,

pode mudar meu relacionamento com o aluno que não te domínio do conteúdo, tudo bem, eu concordo, agora, eu não concordo aceitarem a avaliação para depois citar no meu (00:54:33) onde fala que no meu critério de avaliação eu ganhei zero em tudo, sendo que lá tem assiduidade, entrega do plano, entrega das provas, isso é algo que... eu estive presente nas aulas todos os dias, como que eu não tenho assiduidade, como que eu não tenho controle da aula sendo que eu estive o tempo todo lá. Outra questão que eu acho que poderia ser muito importante é ter disciplinas voltadas para a docência para os alunos, os alunos não têm isso referente a licenciatura, então eu acho que seria importante, hoje em dia não, hoje em dia os alunos eles não têm essa percepção do que é docência, eu acho que mostrar as diretrizes curriculares nacionais e isso não é feito, os alunos não sabem, discutir, dentro dos grandes nomes da enfermagem, como existe (00:55:27) existem homens que são teóricos na enfermagem, isso não é falado, então na parte pedagógica é como se o homem nunca tivesse feito nada, nenhum trabalho importante, só as mulheres, o que fortalece mais uma vez a questão da violência, ele é excluído totalmente, eu acho que eu tenho uma questão importante seria, no momento, Ângela, das aulas práticas falar um pouquinho sobre ter momentos para falar um pouquinho sobre postura, falar um pouquinho sobre como que você conversa, relacionamento pessoal com as pessoas, falar no momento que você tem para reclamar, o momento que você tem para entender, ouvir e saber depois, não, nossos alunos qualquer coisa eles já ficam revoltados e já vão embora, falar que sofreram assédio ou que sofreram algum tipo de violência, acho que para os alunos isso merecia. Outra questão que eu vejo que talvez poderia melhorar a violência contra os professores é a gente... nós acabamos fazendo aquilo que os governantes fazem, não valorizam os professores, e aí, sim, nós replicamos o modelo lá de cima, que vai pelo gestor e que vai, depois, para os alunos, isso acaba passando para os alunos, então eu sempre falo os questionamentos, professor, mas os médicos não nos respeitam, o médico faz isso e eu falo, vocês acham que vocês me respeitam? Nossa, demais, professor, então por que vocês não me chamam de doutor Marcos, porque eu tenho doutorado, então não é ensinado isso para o aluno mais, então você imagina o quanto minimizaria essa violência, dentro desse respeito e aprender esse respeito, humanas, pedagógicas eu acho que fica muito focado assim, como que nós vamos melhorar o ensino, a gente não preocupa em como nós vamos formar um cidadão crítico, um cidadão reflexivo para a vida, para enfermagem está muito além de cuidar, sabe, a gente está muito preocupado com o nosso viés pedagógico, o professor está muito preocupado em vamos formar mão de obra, como se não existisse a parte crítica dentro da enfermagem, e acho que o respeito é acima de tudo, algo que é inerente, esses alunos a gente sabe que eles vem de uma geração diferente da minha e da sua, mas a coisa básica do respeito pode vir geração x, y, x, y, z que eu respeito e ela é inerente, eu sou ainda daquela época, eu acho que professor é algo que eu tenho respeito, eu até hoje chamo todos os meus colegas de trabalho como professor e os alunos não, os alunos estão no meu gabinete, Ângela, acho que isso tem que estar no projeto político pedagógico, eu estou no meu gabinete, o aluno simplesmente abre a porta, eu olho para trás, ah, não é aqui não, e fecha, então eu estou no meu gabinete, normalmente em orientação, os alunos já abrem a porta e começam a falar, sabe, é uma violência muito grande, e isso acho que é no momento da criação do projeto político pedagógico, que aluno é esse que a gente está formando, porque se ele não respeita nem o professor imagina lá fora, como que ele vai, então, assim, ele fica cinco anos com a gente, eu falei da minha esposa, Ângela, lá na área básica não existe isso, é professor o tempo todo,

respeita o horário, chega no horário, ah, professor, eu não consegui chegar por x, y, z, não, tudo bem, você vai entrar, mas você vai entrar no segundo turno da aula, nossa, eu vejo na enfermagem, quando eu falo, aluna, não vamos ao banheiro, vamos fazer isso, só se for um caso extremo e isso não é falado com o aluno nas semanas... não é discutido para o aluno respeitar, porque eu acho que isso é extremamente importante o respeito ao docente que a gente fica perdendo, e acho que principalmente na enfermagem é como se... é muito engraçado que quando eu dou aula para medicina é doutor Marcos, doutorado é algo, sabe, fenomenal, mas enfermagem é como se fosse algo banal, é banalizado, mas eu vejo que nas outras questões não é assim, e eu fico muito triste por isso, principalmente porque eu estou em prática e não me chamam de professor, e aí eu corrijo, como doutor eu não vou corrigir que eu acho que isso é questão... mas chamam os outros colegas, um interno de doutor, mas que respeito é esse, isso é uma violência muito grande para a minha formação, nos dedicamos, você está fazendo agora e sabe da dificuldade que o doutorado é, e o quanto isso é importante para a nossa profissão, a gente tem até que mostrar para os alunos a nossa trajetória de formação, e aí eu queria muito que você frisasse isso aí, deixar os grandes mais, os grandes, as pessoas importantes para a enfermagem, mas cadê o homem, será que não existe nenhum homem? Desde mil oitocentos e bolinha não existiu nenhum homem que até hoje fez um grande marco na enfermagem? Por que não existe, isso é uma violência muito grande com relação ao docente, eu acho que é isso, eu falei que eu sou prolixo demais.

Entrevistadora: Professor, eu tenho aqui nas minhas perguntas, mas eu deixo na última pergunta, algo que você quer pontuar, falar para mim, Ângela, eu senti falta disso, eu gostaria ainda de complementar aqui alguma fala que eu não visualizei durante a sua entrevista, eu queria muito saber se você quer completar, fique à vontade.

Entrevistado: Não, eu acho que eu me senti contemplado, é lógico, assim, eu procuro os pontos negativos, que esse é o objetivo da sua pesquisa, mas tem vários e vários pontos positivos, mas eu acho que dentro do projeto político pedagógico falta participação dos alunos, mas a escolha de um aluno eu acho que nós da parte pedagógica nós temos que pensar na escola de um aluno que te perfil, e quando eu falo é para participar das discussões, não os alunos que querem porque querem, porque tem outras ambições, porque é colega da outra pessoa, não, os alunos tem as mesmas pretensões que nós, é porque falta exemplo, sabe, Ângela, acho que dentro da prática pedagógica e tal, os nossos professores infelizmente não são exemplos para os nossos alunos, então eu acho que, primeiro, tem a questão da formação, essa geração a gente sabe que essa parte é muito discutida, eles também sofrem muita violência, violência de gênero, violência por questão da paternidade, violência até física mesmo, que a gente sabe que essa geração tem sofrido muito e quando ela chega na enfermagem a gente tem que saber pontuar, os alunos querem isso, os alunos, eu acho que a disciplina, Ângela, ela é inerente a tudo que nós formos fazer, que se durante a nossa assistência pedagógica nós não mostrarmos para os alunos a importância da disciplina eles não vão ser bons profissionais e a enfermagem ela vai ser não valorizada como deveria ser como a gente ainda vê hoje, e acho que o professor participar desses momentos de discussão com os alunos e os professores, agora, eu acho, que está muito focado na formação do mestrado e doutorado, e aí na prática pedagógica a situação está ficando política, sabe, se você perceber isso é algo que os alunos reclamam muito,

nossa, professor, a gente quase nem teve aula com o professor xis. Vou te dar só um exemplo, simples, na nossa faculdade nós temos a professora Ana Clara, ela é referência em reprocessamento, por que ela não ministra as aulas para os alunos que vão fazer estágio em docência, não, a principal na instituição pública a graduação que sustenta tudo, ela que é a mola propulsora de tudo, então eu acho que nesse momento está uma discussão, quando a gente fala, professor, você tem interesse pela pós? Sim, porque não é obrigatório, mas não esqueça da graduação, sabe, então esse é o modelo, eu acho que falta, assim, a gente ser visto como modelo pelos nossos alunos, mas não modelo de ser bonzinho, de ser amorosos, isso que eu falo para os alunos, eu quero que vocês me conheçam como professor competente, como um professor que tem conhecimento, não quero que vocês falem para mim, nossa, ele é tão bonzinho, nossa, ele é tão amigo, nossa, ela me leva para tomar café, ela me levou até para a casa dela, e aí na enfermagem é muito engraçado que quando vai falar de professores, Ângela, a maioria dos alunos tem essas qualidade, é difícil, você falar assim, nossa, aquele professor é bom porque ele tem esse e esse conhecimento, primeiro fala dessas questões para depois falar da parte pedagógica, então eu acho que é isso que a gente tem que trabalhar muito, é importante, sim, a parte de prevenção e proteção, sim, mas o professor ele tem que se posicionar como, ele está até como figura de... é um, orientador mesmo, até a definição é isso. Eu também estou com muita expectativa da sua tese, porque eu acho coisas muito importantes, eu queria muito que você divulgasse o máximo que você puder, porque eu acho que é inédito e, assim, que esse seu produto ele chegue às mãos dos alunos e dos nossos gestores, que seja algo oficial, que seja algo que chegue nas mãos dos nossos gestores e dos alunos porque eles estão precisando de literatura sobre isso, sabe, não existe livro, só existem livros sobre questão pedagógica o professor com o aluno, mas não existe nada que norteia o aluno como se portar a frente do processo de ensino e aprendizagem, e se o processo de aprendizagem é duplo, então se nós temos que nos embasar, temos que estudar sobre o processo de ensino e aprendizagem o aluno também porque eu vejo uma dicotomia muito grande, Ângela, se todo os teóricos falam que é troca de conhecimento, não existe hierarquia entre professor e aluno, mas por que a gente tem cursos de formação do processo de ensino e o aluno não, o aluno também tem que ter momentos para que ele saiba o que é ensinar e inclusive, Ângela, na nossa área, porque, assim, o enfermeiro desde o primeiro ano para o aluno ele é um professor, ele está orientando a comunidade, então como que a gente não discute com eles processos pedagógicos sendo que ele é um educador nato, faz parte da enfermagem, eu acho que é isso, falei demais, viu, estou com pena de você, você vê, foi uma hora e dez minutos, mais ou menos, estou com dó de você para transcrever, viu?

Entrevistadora: Uma hora e dez minutos, mas muito proveitosa, foi, assim, professor Marcos, quero muito te agradecer, porque eu sei que diante, foi o início da nossa fala, esse processo que a gente tem vivido aí com tantos problemas de agendas e situações remotas que tem nos desgastado, e esse impacto na nossa saúde emocional, eu chamo de saúde emocional porque não tem como, você tem que arrumar a sua agenda porque está com problemas domésticos, mas que impactam na sua vida, vai impactar mesmo, não tem como, e de saúde e tantos outros como você sabe, então quero te agradecer muito e quero te dizer uma coisa muito importante, você despertou, qua na sua fala emergiram tantas categorias que para mim já estavam saturadas e que, no entanto, elas não saturaram, e isso é muito importante na área qualitativa, você sabe disso, que

maravilha foi que as categorias foram surgindo e eu consegui visualizar todas durante a nossa entrevista. Então, assim, quero sim, acredito que esse trabalho vai ter um produto que possa contribuir para a academia no seu contexto geral, mas também, talvez, quem sabe até condições político pedagógicas porque não existe nada que nos defenda enquanto professores, eu tenho amadurecido isso, então agradeço mesmo de coração, muito obrigada, Marcos, viu, e eu quero fazer um trabalho com você, você pode ter certeza nós vamos escrever um artigo, com a professora Dolores, eu acho que vai ser fantástico, pegando o gancho...

Entrevistado: Fala alguma coisa, Ângela, assim, os desafios de ser heterossexual na enfermagem, eu acho que isso, se eu propor alguma coisa assim você imagina quando isso, mas eu acho que isso é violência de sexo, não de gênero, violência de sexo dentro da enfermagem, sexo homem e aí entra depois o hetero, é um desafio, por isso que a maioria dos homens, Ângela, eles acabam saindo um pouco da enfermagem, porque eles meio que... a gente não se sente confortável, a palavra é essa, e não pelos outros colegas, mas pelos próprios pares.

Entrevistadora: Verdade, e às vezes a gente está tão acostumado a olhar no espelho que não vê nem as rugas aparecendo, né, Marcos, é tipo alguém falar, olha aqui, presta atenção aqui. Mas olha, foi fantástico, muito obrigada, mesmo, viu.

Entrevistado: Eu que agradeço, tá?

Entrevistadora: Bom dia. Tchau, obrigada professor.

Entrevistado: Agradeço pelo trabalho.

Entrevistadora: Obrigada, muito obrigada.

Entrevistado: Obrigado, professora, tchau.

Entrevistadora: Tchau.

01:11:29

**Categoria central**	**Subcategorias dimensionadas**	**Codificação focalizada**
**Tangenciamento e multifatorialidade da violência contra o docente: nuances vivenciadas na [illegible]**	**Ação e reação: a violência não acontece sozinha**	O desrespeito* como expressão de violência e sua relação com a hierarquia (E1, E2, E3, E4, E5, E6, E8, E10, E11).
		TDIC* como a linguagem atual e velada de violências (E4, E5, E7, E8, E9, E10).
		Necessidade de capacitação docente para enfrentamento da violência* no ambiente acadêmico (E1, E2, E3, E4, E5, E6, E7, E8, E9, E10, E11).

		Repressão e intimidação como expressão do medo* e limitações de enfrentamentos (E2; E4; E6; E7; E9; E11).
	**Cultura institucional e gênero**	Violência de gênero* relacionada ao histórico-cultural (homem-mulher). A Enfermagem é uma profissão feminina (E4, E7, E9, E10, E11)?
		A violência institucional*, simbólica ou não, expressa por questões de domínio, regras, regulamentos e valores (E1, E2, E3, E4, E6, E7, E8, E9, E10, E11).
	**Percepção de violência contra o docente**	As nuances da agressão*, física, mental, cultural e social (E9, E10, E11).
		A fragilidade das relações interpessoais* e o perceptível adoecimento* do professor (E2, E3, E4, E5, E7, E9, E11).

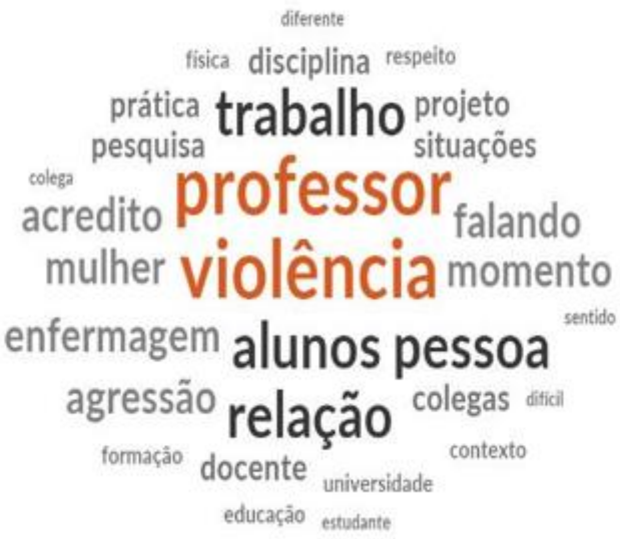

Supplement: 0034-7167-reben-76-01-e20210865-sup01 [file 0034-7167-reben-76-01-e20210865-sup01.pdf]
